# Trifunctional DOPO‐Engineered Polypropylene Separator With Li⁺‐Concentrating Interfaces for High‐Safety Lithium‐Ion Batteries Under Extreme Conditions

**DOI:** 10.1002/advs.202516139

**Published:** 2025-10-14

**Authors:** Wende Yi, Wufei Tang, Weikang Su, Keren Shi, Qiaowei Xiao, Ziyan Wang, Xiaoyu Li, Jingyang Mu, Huiqin Yao, Zhihan Peng

**Affiliations:** ^1^ College of Chemistry and Bioengineering Hunan University of Science and Engineering Yongzhou 425199 China; ^2^ State Key Laboratory of High‐Efficiency Utilization of Coal and Green Chemical Engineering College of Chemistry & Chemical Engineering Ningxia University Yinchuan Ningxia 750021 China; ^3^ General Hospital of Ningxia Medical University College of Basic Medicine Ningxia Medical University Yinchuan 750004 China; ^4^ College of Materials Science and Engineering Donghua University Shanghai 201620 China

**Keywords:** density functional theory (DFT), high‐temperature resistance, lithium‐ion batteries, mechanical strength, thermal stability

## Abstract

This study develops a cross‐linked polymer modified polypropylene (PP) separator for lithium‐ion batteries, using DOPO (9,10‐dihydro‐9‐oxa‐10‐phosphaphenanthrene‐10‐oxide). The separator features surface‐rich polar functional groups (such as ‐NH_2_) and bonded structures (P‐O, P = O), enhancing Li^+^ ion migration via dipole‐dipole interactions. The modified separator demonstrates improved microstructural stability, thermal stability (>90 °C), and mechanical strength (>200 MPa). Batteries using this separator maintain excellent capacity retention (105.2 mAh g^−1^) after high‐temperature cycling (130 °C at 2C) and show good flame retardancy (self‐extinguishing). Density functional theory (DFT) calculations explain the Li^+^ enrichment mechanism and the separator's thermal/flame‐retardant properties. This work pioneers the multifunctional use of DOPO in lithium‐ion battery separators, combining flame retardancy, enhanced Li^+^ conductivity, and improved stability, offering a novel approach to producing safer, high‐performance separators for large‐scale applications.

## Graphical abstract

A DOPO‐based cross‐linked polymer creates a multifunctional separator for lithium‐ion batteries. It concurrently addresses safety (flame retardancy, thermal stability) and performance (enhanced Li^+^ transport, capacity retention), pioneering a novel approach to high‐performance battery design.

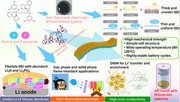

## 1 Introduction

The pressing need to develop advanced renewable energy systems has heightened demands for efficient and secure energy storage technologies.^[^ ^1^, ^2^, ^3^, ^4^ ^]^ As the core energy storage solution, lithium‐ion batteries face safety bottlenecks primarily stemming from thermal runaway risks,^[^ ^5^, ^6^ ^]^ with structural failure of separators and inadequate material thermal stability often serving as key contributing factors.^[^ ^7^, ^8^, ^9^, ^10^, ^11^ ^]^ Commercial polyolefin separators exhibit poor heat resistance, prone to shrinkage and melting, making it difficult to suppress internal short circuits.^[^ ^12^, ^13^, ^14^, ^15^, ^16^ ^]^ Although solutions such as polyimide and ceramic coatings exist to enhance thermal resistance and flame retardancy, their application remains constrained by complex manufacturing processes,^[^ ^17^ ^]^ high costs, and adverse effects on electrochemical compatibility.^[^ ^18^, ^19^ ^]^ Particularly in high‐energy‐density systems, achieving synergistic high flame retardancy,^[^ ^20^ ^]^ superior interfacial stability,^[^ ^21^ ^]^ and effective ion conduction in separators remains a core challenge.^[^ ^22^, ^23^, ^24^ ^]^

**Figure 1:**
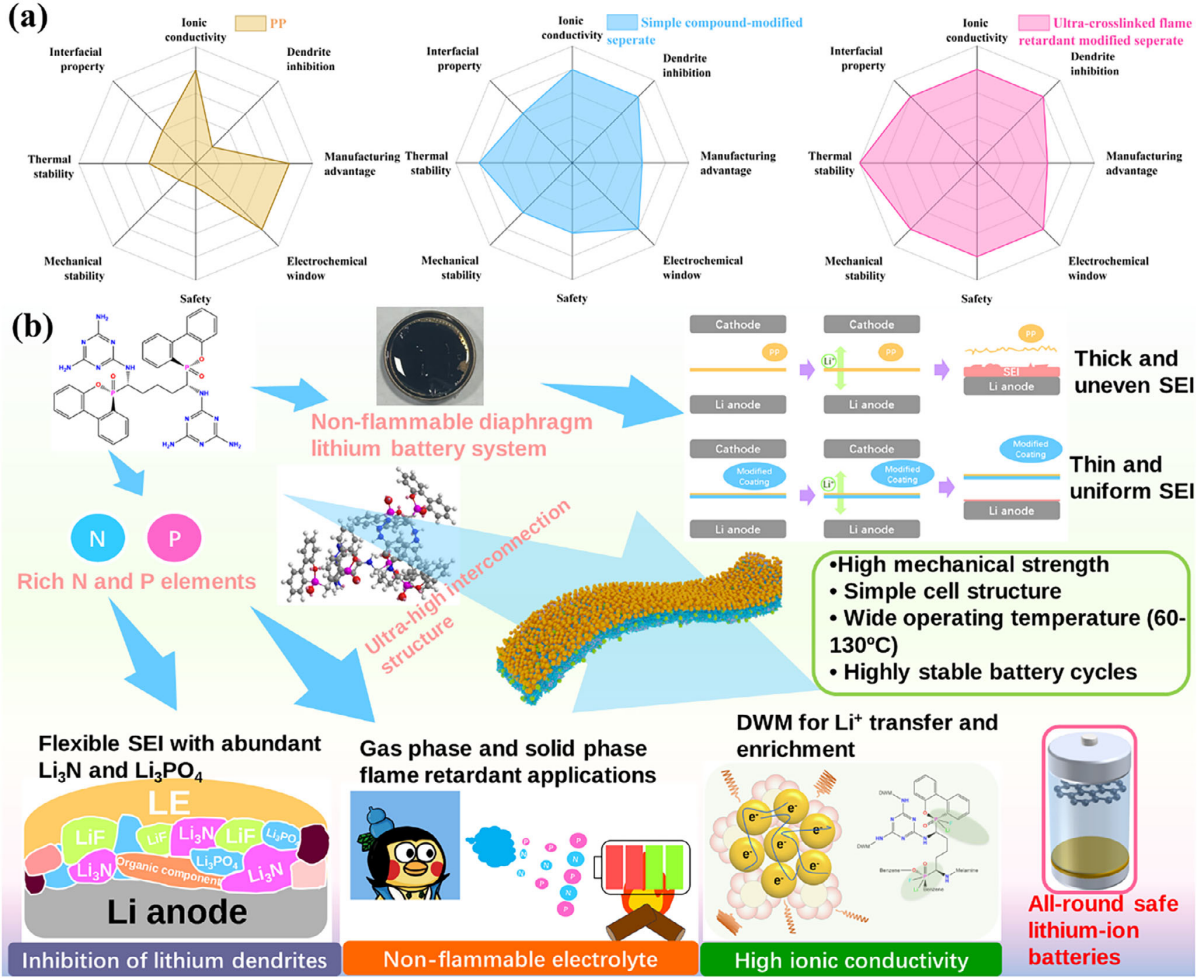
a) Comparison of different types of separators b) Mechanism of action of modified flame‐retardant cross‐linked networks in lithium batteries.

To systematically address these obstacles, this study proposes a multifunctional composite separator based on a multilayer synergistic design concept.^[^ ^25^, ^26^, ^27^, ^28^, ^29^ ^]^ This design achieves synergistic enhancement of thermal safety and electrochemical performance by precisely constructing an ultrathin functional coating on a polypropylene (PP) separator substrate.^[^ ^30^, ^31^, ^32^, ^33^, ^34^ ^]^ The coating comprises a composite of micro‐nano phosphorous‐nitrogen flame retardants (DWM), bio‐based char‐forming agents (CH), and conductive carbon black (SP). At extremely low loading levels, it delivers multi‐layered protection and interfacial regulation:^[^ ^35^, ^36^, ^37^ ^]^ the DWM and CH exhibit synergistic gas‐char flame retardancy, simultaneously releasing non‐flammable gases to dilute oxygen and catalyzing polymer matrix char formation, thereby effectively suppressing flame propagation and delaying thermal runaway.^[^ ^38^, ^39^ ^]^ Crucially, the abundant polar functional groups (‐NH_2_, P–O, P = O) on the DWM surface preferentially adsorb electrolyte decomposition products via strong dipole interactions.^[^ ^40^ ^]^ This guides the formation of a uniform, stable,^[^ ^41^ ^]^ LiF‐rich solid electrolyte interphase (SEI) film at the electrode interface,^[^ ^42^ ^]^ significantly enhancing lithium ion migration and interface stability while suppressing dendrite growth and side reactions.^[^ ^43^ ^]^ To further mitigate electrode polarization and kinetic lag caused by the insulating properties of conventional flame retardants,^[^ ^44^ ^]^ this study innovatively incorporates an SP conductive network.^[^ ^45^ ^]^ This not only provides micro‐conductive pathways for electron transport,^[^ ^46^ ^]^ reducing interfacial impedance,^[^ ^47^ ^]^ but also enhances coating adhesion strength^[^ ^48^ ^]^ and adaptability to electrode volume changes,^[^ ^49^ ^]^ thereby balancing high safety with extended cycle life(**Figure** **1**a).^[^ ^50^ ^]^

This composite separator exhibits high thermal stability (≥250 °C) and mechanical strength (>300 MPa), while effectively enhancing ionic conductivity and lithium ion mobility(Figure 1b). It demonstrates superior electrochemical compatibility and cycling durability. This research not only establishes a novel, scalable architecture for high‐safety separators but also pioneers a multifunctional integrated coating design approach, holding significant promise for advancing high‐energy‐density lithium metal batteries.

## 2 Results and Discussion

### 2.1 Preparation and Characterization of Flame‐Retardant Modified Membranes

**Figure 2:**
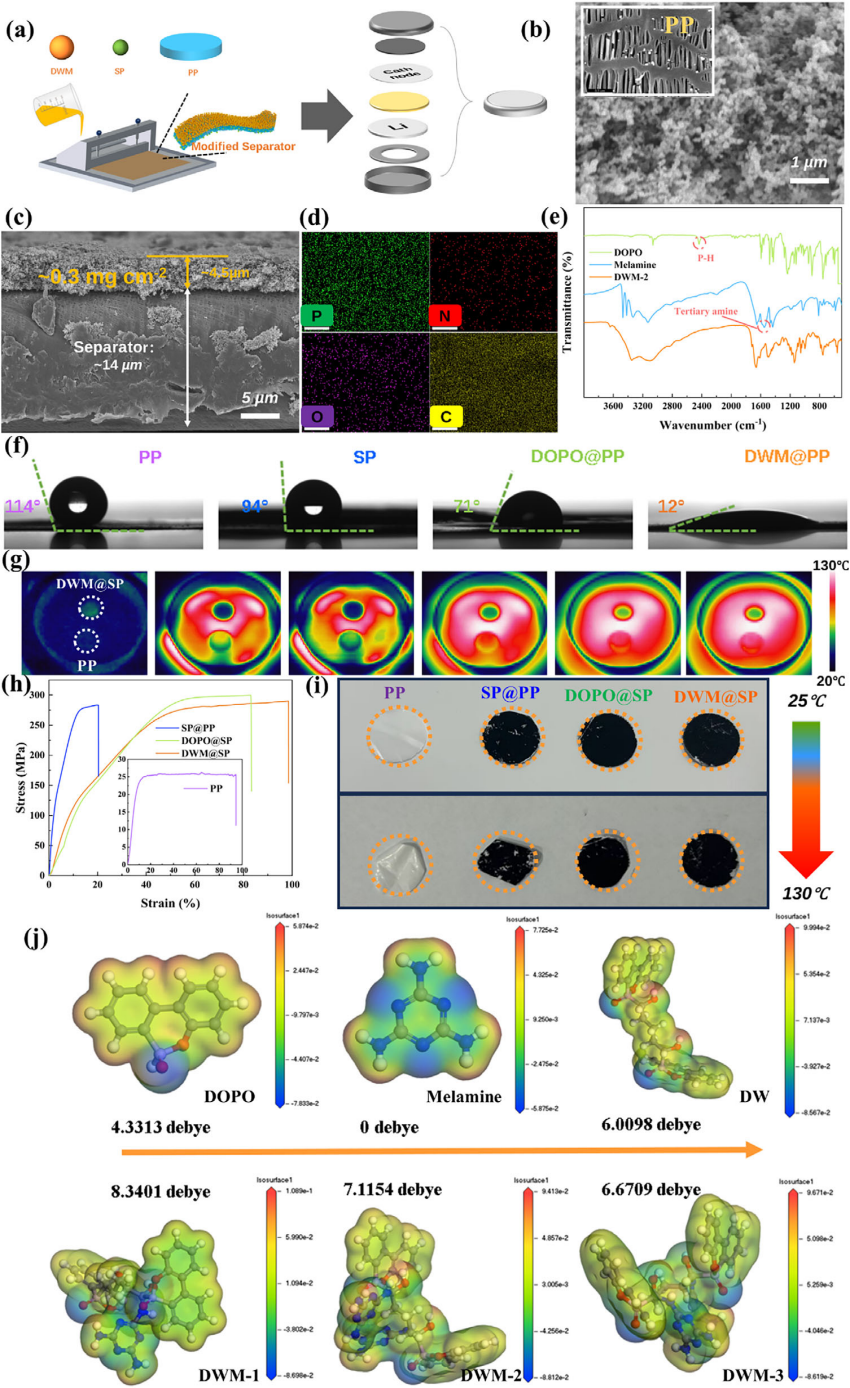
a) Preparation process of modified separator; b) SEM image of flame‐retardant coating (original PP separator in the upper left corner) and c) cross‐sectional SEM of modified separator; d) EDS elemental spectrum of modified flame‐retardant coating. e) FTIR of raw materials and synthesized flame retardants. f) Contact angle of electrolyte on different separators. g) Thermal imaging of PP separator and DWM@sp separator. h) Tensile stress of different separators. i) High‐temperature ageing test of different separators. j) Dipole moment and electrostatic potential of flame retardants with different ratios of raw materials and synthesized materials.

**Figure** **2**a shows a schematic diagram of the slurry coating process, a scalable coating production process widely used in industrial production. The prepared DWM and SP particles are insoluble in solvents, making them suitable for coating processes. The designed DWM flame retardant has a small square structure that can be well combined with SP (Figure 2b). In the DWM@PP coating constructed using the coating method, the particles are uniformly distributed and have a rich pore structure, providing channels for lithium‐ion transport. After drying, DWM and SP are uniformly intertwined in the DWM@PP coating. Notably, the thickness of the DWM@PP coating is only 4.5 µm, with a surface loading of 0.3 mg cm^−2^, which is attributed to the low‐density characteristics of DWM and SP (Figure 2c). Figure 2d shows the EDS mapping image of the DWM@PP coating, with P, N, O, and C elements uniformly distributed in the coating, confirming the uniformity of DWM in the coating. To further investigate the synthesis process of DWM, its chemical structure was characterized using FTIR, with Figure 2e showing the FTIR spectra of the synthetic raw materials DOPO, Melaine, and the final product DWM. In the DWM spectrum, characteristic absorption peaks appear at 879 and 1074 cm^−1^, attributed to P‐O groups; at 1261 cm^−1^, there is a characteristic absorption peak for P = O, with these phosphorus‐containing functional groups originating from the polyphosphate structure in DOPO. Additionally, a broad absorption peak appears between 3000 and 3500 cm^−1^, originating from the N‐H and O‐H vibrations in Melaine, indicating that the electrostatic assembly process did not disrupt the phosphorus‐ and nitrogen‐containing chemical groups in DOPO and Melaine. The abundant P‐O, P = O, and amino groups in DWM facilitate the wetting of carbonate‐based electrolytes.^[^ ^51^, ^52^, ^53^, ^54^, ^55^, ^56^ ^]^ To validate the feasibility of this synthesis method, the energy required for each step of bond breaking (e.g., H atoms) in the DOPO substrate and the binding energy of the two substances after bond breaking were calculated, as shown in the Figures S2 and S3 (Supporting Information),We found that the energy required for DOPO to dissociate H or melamine to dissociate one H is far smaller than their binding energy, proving that the cross‐linked flame retardant at the synthesis site has an extremely stable structure. To demonstrate that the selected ratio of DWM (DWM‐2) is the optimal ratio, FTIR testing (Figure S4, Supporting Information), interfacial binding energy between DWM and PP at different ratios (Figure S5, Supporting Information), density of states (Figure S6, Supporting Information), and Lumo/homo (Figure S7, Supporting Information) were conducted at different ratios. The results show that DWM‐2 stands out from the perspective of synthesis and binding energy. As shown in Figure 2f, the contact angle test results for the separator with carbonate‐based electrolyte indicate that the PP separator has poor wettability with the electrolyte, with a contact angle of 114°. Similarly, the wettability of SP and DOPO coatings did not show significant improvement, with SP coatings exhibiting a contact angle of 94° and DOPO coatings exhibiting a contact angle of 71°. However, when DWM coatings were present, the contact angle between the separator and electrolyte decreased to ≈12°, with the largest wetted area (The PP base film used in this study was prepared via a blown film process, resulting in higher surface crystallinity and greater density, thus exhibiting strong hydrophobicity (contact angle >100°). This clarification is provided to avoid direct comparison with data from commercially available membranes produced by different processes.).This is primarily attributed to the excellent affinity between the DWM layer and electrolyte, as well as the capillary effect resulting from its micro‐nano porous structure. The loose porous structure of the SP layer also promotes electrolyte wetting. The improved electrolyte wettability (Figure S8, Supporting Information) facilitates Li^+^ transport during charging and discharging, reduces polarization, and significantly enhances electrochemical performance. As shown in Figure 2g, when PP and DWM@PP separators are heated to 130 °C, it is observed that the DWM@PP separator maintains a temperature of ≈50 °C even at high temperatures, while the PP separator rapidly heats up to a higher temperature after ≈3 min of heating, indicating that DWM modification significantly enhances the thermal stability and thermal insulation performance of the PP separator. Notably, compared to SP@PP, the thermal stability of DOPO@SP is lower than that of DWM@SP but better than that of SP@PP (Figures S9 and S10, Supporting Information).^[^ ^57^ ^]^

A comparative analysis of tensile properties (Figure 2h) clearly demonstrates that, thanks to significantly enhanced interfacial bonding energy with the PP matrix (Figure S11, Supporting Information), the DWM@PP‐modified separator exhibits superior mechanical performance advantages—it can withstand higher stresses and greater deformations during tensile testing; Although DOPO@PP and SP@PP also exhibit high tensile strength, their durability is significantly inferior to that of DWM@PP(The tensile stress test samples and battery separators were sourced from the same batch.), further confirming the critical role of strong interfacial bonding in enhancing the overall stress‐strain tolerance limit of the separator.^[^ ^58^, ^59^, ^60^, ^61^ ^]^

Additionally, the four separators were subjected to high‐temperature ageing tests in an oven. Figure 2i illustrates the thermal shrinkage behavior of each separator after 30 min at 130 °C. The PP separator experienced severe thermal shrinkage, with an area shrinkage rate of 30.6%, while the SP‐modified separator had an area shrinkage rate of 18.8%, and the DOPO@SP separator exhibited a lower shrinkage rate. In contrast, the DWM@SP modified separator showed almost no shrinkage, with a shrinkage rate of only 2.1%. When the temperature was increased to 160 °C, the edges of the PP separator became transparent, indicating melting, with a thermal shrinkage rate of 47.7%. After DWM modification, the thermal shrinkage rate decreased significantly, demonstrating that the introduction of DWM effectively enhances the thermal stability of the separator, enabling it to resist short circuits during severe thermal runaway in batteries, thereby mitigating the risk of fire or explosion.^[^ ^62^, ^63^ ^]^

To verify the compatibility of DWM flame‐retardant materials with lithium‐ion battery separators, we simulated the dipole moment and electrostatic potential of DWM at different ratios and each synthesis step using Materials Studio software (Figure 2j). A higher dipole moment can improve separator wettability through interaction with the electrolyte, enhancing ionic conductivity. Additionally, polar groups may promote the solvation/desolvation process of Li⁺ through dipole‐ion interactions, thereby reducing interfacial impedance. However, an excessively high dipole moment may exacerbate electrolyte decomposition and corrosion of the lithium anode. The results obtained are consistent with those from FTIR testing (Figure S4, Supporting Information). DWM‐2 (hereinafter referred to as DWM) exhibits better binding compared to the other two ratios and has moderate dipole moment and electrostatic potential.^[^ ^64^ ^]^

**Figure 3:**
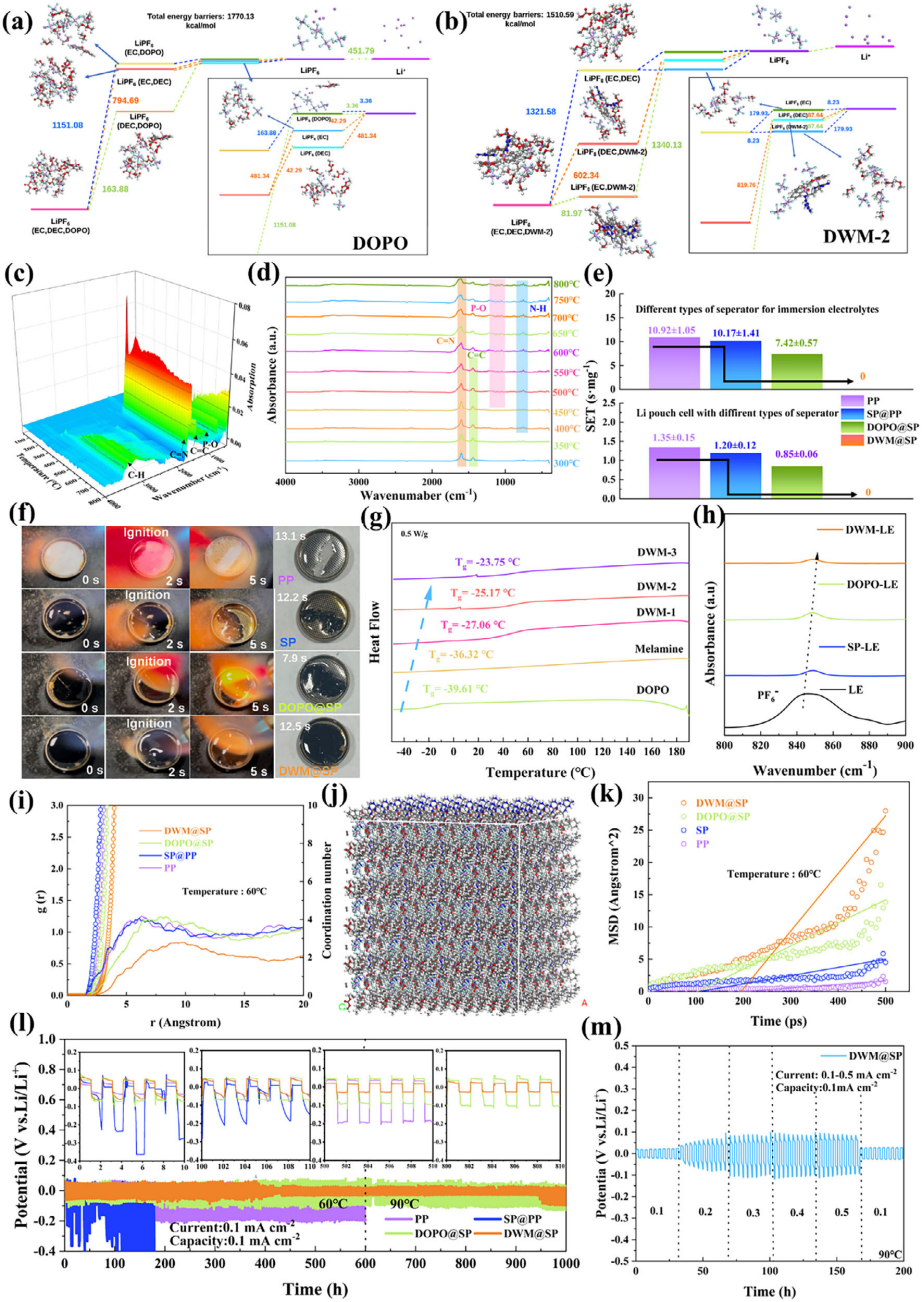
a) Desolvation energy barrier of DOPO b) Desolvation energy barrier of DWM‐2 c) TG‐FTIR 3D diagram of DWM and d) infrared spectrum at representative temperatures. e) SET of soft‐pack batteries with different separators and their assemblies. f) Open combustion experiments of different separators immersed in electrolyte. g) DSC of raw materials and their synthesized flame retardants. h) Infrared spectra of hexafluorophosphate ions in electrolyte‐immersed coating materials. i) Radial distribution function and lithium ion coordination number of different separators in a battery system at 60 °C, and j) corresponding computational models and average displacement of Li ions at the corresponding temperatures. k) The mean displacement of lithium ions in lithium‐ion battery systems with different separators. Charge‐discharge curves of lithium symmetric cells at 60 °C/90 °C for lithium‐ion batteries with different separators l) and rate performance of DWM@SP (m).

To further investigate whether the DWM cross‐linked network provides better dipole‐ion interactions to promote the solvation/desolvation process of Li⁺, the solvent free energy barrier was calculated using the Forcite module of Materials Studio. The results are consistent with the aforementioned findings, with DWM‐2 (**Figure** **3**b) exhibiting a lower energy barrier compared to DOPO (Figure 3a) and other ratios (Figures S12 and S13, Supporting Information) have lower energy barriers, which significantly promotes the solvation/desolvation of Li^+^.

To further investigate the impact of DOPO and DWM modifications on thermal stability, thermal gravimetric analysis (TGA) was conducted on DOPO and DWM. The results showed that the degradation temperature of the small molecule DOPO ranges from 110 to 230 °C, while DWM exhibits significantly enhanced thermal stability, with decomposition occurring between 150 and 800 °C. Additionally, DOPO exhibits almost no char residue, as it completely volatilizes at high temperatures without undergoing internal reactions; in contrast, DWM shows a char residue rate of 48.9% at 800 °C, indicating that cross‐linking reactions enhance thermal stability and effectively form a thermal barrier (Figure S14, Supporting Information). Furthermore, thermal gravimetric analysis (TGA) of the modified coatings applied to PP membranes (Figure S15, Supporting Information) revealed that DWM@SP membranes exhibit superior thermal stability. While the thermal decomposition temperature of DOPO@SP is lower than that of DWM@SP, it is noteworthy that both PP and SP@PP are nearly completely lost at temperatures approaching 500 °C. This demonstrates that the introduction of flame retardants significantly enhances the thermal stability of the membranes.^[^ ^65^, ^66^, ^67^, ^68^ ^]^

Furthermore, TG‐FTIR analysis revealed the gaseous products of DWM at high temperatures. The appearance of C = N and C‐H cleavage fragments indicates the thermal decomposition of the cross‐linking agent Melaine. Additionally, the signal of the P‐O fragment intensifies at 300 °C, indicating that the cleavage reactions of P─O─C bonds and P═O play a crucial role in suppressing the combustion of carbonate‐based electrolytes (Figure 3c,d).

To visually assess the flame‐retardant effect of DWM on the separator and electrolyte, SET(Self‐extinguishing Time, SET: The duration for which the electrolyte continues to burn after removal of the external ignition source, used to evaluate its flame‐retardant properties.) tests were conducted on electrolyte‐impregnated separators and assembled pouch batteries (Figure 3e,f). The results showed that the PP separator immediately ignited upon contact with the flame, with an SET value of 0.60 s mg^−1^. Although the SP separator is more resistant to combustion than the PP separator, the modification effect is limited, with an SET of 0.46 s mg^−1^. After introducing DOPO, the flame‐retardant performance of the DOPO@SP separator was significantly improved, with the SET decreasing to 0.36 s mg^−1^. The DWM@SP modified separator exhibited even higher flame‐retardant efficiency than DOPO, with an SET of only 0.32 s mg^−1^. The high flame‐retardant performance of DWM stems from the release of phosphorus‐related free radicals over a broader temperature range, which effectively suppress the active free radicals generated by the electrolyte and separator. The SET of the two‐cell soft‐pack battery is similar to the results mentioned above.^[^ ^69^ ^]^

The glass transition temperature (*T*_g_) of the original DOPO and its DWM composites with different ratios was estimated by differential scanning calorimetry (DSC) (Figure 3g). DOPO exhibited the lowest *T*_g_ value of −39.61 °C. Although cross‐linking increased the *T*_g_ value of LPE by occupying free volume and coordination effects, its *T*_g_ value remained far below room temperature (RT). The *T*_g_ values of melamine, DWM‐1, DWM‐2, and DWM‐3 were −36.32, −27.06, −25.17, and −23.75 °C, respectively. The melting temperature of the separator determines whether the battery experiences internal short circuits at high temperatures and is a key parameter affecting battery safety. Analysis of the melting behavior of the separator via DSC, as shown in the Figure S16 (Supporting Information), indicates that the melting temperature (T_m_) of the PP separator is ≈165 °C, while the T_m_ of SP@PP, DOPO@SP, and DWM@SP modified separators is similar to that of the PP separator, indicating that the modified coatings have not altered the inherent melting point of PP. However, the melting enthalpy of DOPO@SP and DWM@SP modified separators is significantly reduced by 38% compared to the PP separator, indicating that the DOPO and DWM@SP layers slow down the melting of the PP separator.^[^ ^70^ ^]^

Furthermore, due to the abundant electron‐rich functional groups in DWM, it forms dipole‐dipole interactions with the electron‐deficient lithium atoms in LiF, thereby promoting the chemical adsorption of LiF. The adsorbed solution exhibits a significant reduction in UV–vis spectroscopic intensity, indicating that DWM possesses strong adsorption capacity for LiPF_6_ (Figure S17, Supporting Information). However, when only SP powder is used, the UV–vis spectrum of the LiPF_6_ solution remains almost unchanged, indicating that SP is not an effective LiF adsorption material. To further demonstrate the constraint effect of different coatings on lithium salt ions, FTIR testing was conducted on different coated materials after immersion in lithium salt. As shown in Figure 3h, the characteristic peak of the counterion PF_6_^−^ at 846 cm^−1^ in the lithium salt exhibits a slight blue shift after the introduction of the modified coating, indicating stronger interaction between PF_6_^−^ and DWM, which enhances the constraint on counterions, not only promoting the dissociation of lithium salts to provide freer Li^+^, but also increasing t_Li_^+^. DFT calculations of the bandgap (Figure S18, Supporting Information) and adsorption energy (Figure S19, Supporting Information) for different separators further confirm this. The bandgap of the DWM@SP reaches 4.928 eV, which provides a good transmission environment for the electrolyte and ensures the strong insulation of the separator. The bandgap of the DOPO@SP also reached 4.344 eV, which proves that the addition of flame retardant plays a very important role in the separator. Additionally, the incorporation of the modified flame retardant DWM confers a high adsorption energy (3.28 × 104 eV) to the separator, which is 1.94 times higher than that of the PP separator (1.69 × 104 eV).

Molecular dynamics (MD) simulations were conducted using Material Studio software to further investigate the microphase structure of fragment separation and the interaction between Li^+^ and the coating. The radial distribution function (RDF) and coordination number (CN) were calculated using the Forcite module. Figure 3i shows the radial distribution function of Li ions with different separator coatings. Compared to PP, SP@PP, and DOPO@SP, the distance between Li ions and the DWM@SP coating decreases, but the complexation ability of Li^+^ weakens. The results showed that the average coordination number of Li in the DWM@SP was ≈4.5, which was lower than that of ≈9.0, 7.9 and 16.3 in PP, SP@PP and DOPO@S. This result indicates that the binding interaction force between Li^+^ and the polymer separator in DWM@SP is lower than that in pure PP. The mean square displacement (MSD) was also calculated to study the diffusion coefficient of Li^+^. This is shown in Figure 3k. The diffusion coefficient of lithium ions in the FDWM@SP system is 7.07 × 109 cm^2^ ps^−1^, which is significantly higher than that in the PP system (1.05 × 10^7^ cm^2^ ps^−1^), the SP@PP system (2.96 × 10^7^ cm^2^ ps^−1^), and the DOPO@SP system (1.62 × 10⁸ cm^2^ ps^−1^), indicating that lithium ion transport is faster in DWM@SP. Notably, even at 90 °C (Figures S20 and S21, Supporting Information)or even 130 °C (Figures S22 and S23, Supporting Information), DWM@SP exhibits superior performance.

To quantitatively assess the impact of flame retardant introduction on the intrinsic redox tolerance of the separator, this study employed linear sweep voltammetry (LSV) for systematic analysis (Figure S24, Supporting Information). Key findings indicate that, based on a planar electrode configuration, the DWM@SP‐modified separator exhibits exceptional electrochemical stability under stringent 90 °C conditions, with an oxidation decomposition potential as high as 5.92 V, significantly broadening the electrochemical stability window (ESW). This performance far exceeds that of the DOPO@SP separator (4.97 V), highlighting the advantages of the cross‐linked immobilization strategy in enhancing high‐temperature tolerance. Comparative experiments further revealed that at 60 °C, Conventional PP diaphragm and SP@PP diaphragm are at 2.41 V and 0.67 V, respectively, undergo significant oxidation decomposition, severely limiting their stability. Therefore, the wide ESW characteristics exhibited by the DWM@SP flame‐retardant modified separator lay a critical material foundation for its application in high‐voltage lithium metal battery systems and achieving higher energy storage density. Notably, the DWM@SP‐modified separator demonstrates significant efficacy in suppressing lithium dendrite formation. Constant current cycling tests (Li/DWM@SP/Li symmetric battery) indicate that even under high‐temperature conditions of 60 or 90 °C, the lithium deposition/stripping process exhibits low polarization overpotential. More notably, this battery system achieves ultra‐long stable cycling exceeding 1000 h, accompanied by only extremely minor voltage fluctuations (Figure 3l), demonstrating exceptional cycling robustness. A detailed analysis of the voltage curves at different time points during cycling revealed no micro‐short circuit signals (such as voltage drops) caused by lithium dendrite penetration. To further understand the dendrite suppression mechanism, this study conducted multi‐scale interface characterization. Scanning electron microscope (SEM) images (Figures S25 and S26, Supporting Information) show that the DWM@SP separator forms a uniform, dense interface layer on the surface of the lithium metal anode after cycling, compared to the DOPO@SP separator. Cross‐sectional analysis further confirms that Li⁺ deposition primarily occurs below this interface layer, indicating that it effectively guides the planar deposition of lithium. Energy‐dispersive X‐ray spectroscopy (EDS) elemental distribution maps confirm that elements such as C, O, F, P, and N are highly uniformly distributed within the interface layer. X‐ray photoelectron spectroscopy (XPS) depth profiling (Figure S27, Supporting Information) revealed the chemical composition of this interface layer: it is primarily composed of decomposition products from DWM@SP, organic fragments derived from LiPF_6_, and inorganic lithium compounds (such as high‐ionic‐conductivity LiF, Li_3_N, as well as Li_2_CO_3_, Li_3_PO_4_, etc.). This in situ‐formed, inorganic‐rich, dense mixed interface layer (SEI) not only ensures efficient Li^+^ transport but, more importantly, effectively suppresses local current focusing and the nucleation and growth of lithium dendrites through its uniform mechanical strength and ionic conductivity properties. In stark contrast, symmetric cells using SP@PP separators exhibited a sharp increase in overpotential (>1.0 V) after only 160 h of cycling and failed within 200 h (Figure 3l). In addition, Li/DWM@SP/Li cells are tested in a wide range of tests where the current density is gradually increased from 0.1 to 0.5 mA cm^−2^ (Figure 3m), the system maintained stable operation, with the voltage increase remaining within an acceptable range, further confirming the modified separator's excellent interface stability and ionic transport capability under dynamic conditions.^[^ ^71^ ^]^

### 2.2 Electrochemical Performance of the Li Metal Batteries

**Figure 4:**
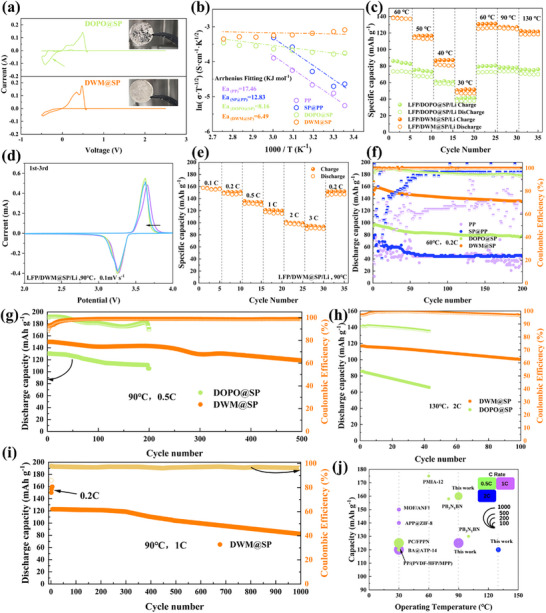
a) CV cycling curves of DOPO‐DWM lithium symmetric batteries and electrode images after cycling b) Ionic conductivity and activation energy of lithium batteries assembled with different separators c) Rate performance of DOPO and DWM batteries under heating and cooling conditions d) CV cycling curves of LFP/Li batteries assembled with DWM at 90 °C and e) rate performance at different current densities. f) Long‐term cycling performance of different batteries at 60 °C and 0.2C. g) Long‐term cycling performance of DOPO and DWM batteries at 90 °C and 0.5C. h) Long‐term cycling performance of DWM batteries at 130 °C and 2C. i) Long‐term cycling performance of DWM batteries at 90 °C and 1C. j) Comparison with other literature.

To validate the feasibility of flame retardant coatings in actual all‐metal lithium batteries (using lithium iron phosphate (LFP) cathodes), this study systematically evaluated their electrochemical behavior. The core challenge lies in the fact that traditional binders such as polyvinylidene fluoride (PVDF) struggle to effectively suppress the continuous leaching of flame retardant components (such as DOPO) into the electrolyte. Particularly concerning is that dissolved DOPO significantly accelerates the degradation process of the lithium metal anode. To confirm this, we conducted a detailed comparison of the cyclic voltammetry (CV) characteristics of Li//Li symmetric cells using DOPO@SP and DWM@SP modified separators. As shown in **Figure** **4**a, the battery assembled with DOPO@SP separators exhibited abnormal redox peaks in the lithium deposition potential range (−1 to 3 V vs. Li^+^/Li), attributed to the nucleophilic attack of the electrophilic phosphorus centers in dissolved DOPO molecules on the lithium anode. This side reaction caused severe corrosion of the lithium metal surface after cycling. In stark contrast, the CV curves of batteries using DWM@SP‐modified separators did not exhibit this abnormal peak, and the lithium anode remained intact and shiny after cycling. This clearly demonstrates that the cross‐linked network achieved through molecular design effectively fixes the functional components, completely eliminating the parasitic side reaction pathways between the DWM@SP coating and the lithium metal. The electrochemical stability of the actual system is influenced by the synergistic effects of the electrode interface and lithium salt chemistry. Therefore, we systematically measured the ionic conductivity (σ) of different separators over a wide temperature range (25–90 °C) using electrochemical impedance spectroscopy (EIS) (Figure S28, Supporting Information). At room temperature (25 °C), the SP@PP separator exhibited the highest σ value (0.252 mS cm^−1^). However, at 60 °C, the DWM@SP membrane demonstrated the most outstanding ionic conductivity (σ ≈ 2.09 mS cm^−1^), significantly outperforming the PP substrate (0.0285 mS cm^−1^), SP@PP (0.053 mS cm^−1^), and DOPO@SP (1.38 mS cm^−1^). Notably, the ionic conductivity of DWM@SP at high temperatures was improved by 1–2 orders of magnitude compared to the original PP separator. The ionic conductivity of a separator is fundamentally a function of the carrier mobility (µ) and the concentration of mobile lithium ions (c) (σ = µ·c).^[^ ^72^, ^73^ ^]^

The transport of ions within the separator strongly depends on the dipole relaxation and segmental motion capability of the polymer matrix. The activation energy (*E*_a_) calculated using the Arrhenius equation provides key insights into temperature dependence: *E*_a_ values of:P P, SP@PP, DOPO@SP, and DWM@SP were 17.46, 12.83, 81.6, and 6.49 kJ mol^−1^, respectively (Figure 4b and Table S2, Supporting Information). The activation energy *E*_a_ quantifies the temperature sensitivity of the ionic hopping energy barrier. The significant differences in *E*_a_ among different membranes stem from the differential effects of lithium salt concentration on polymer chain mobility (i.e., the “salt plasticization” or “salt stiffening” effect). DWM@SP exhibits a high σ value at room temperature, primarily due to its lowest *E*_a_, indicating the smallest internal ion migration energy barrier. As temperature increases, polymer chain segment motion intensifies. At this point, the σ value is jointly regulated by carrier concentration (c) and activation energy (*E*_a_). At 60 °C, the σ values of both DOPO@SP and DWM@SP significantly increase, but the increase in DOPO@SP is limited by its extremely high *E*_a_ (81.6 kJ mol^−1^), suggesting strong ion‐polymer interactions or restricted segment motion within the material. When the temperature is further increased to 90 °C, thermal energy is sufficient to overcome most migration energy barriers, and the influence of *E*_a_ weakens. At this point, the effective lithium ion concentration (c) capable of long‐range migration in the separator becomes the dominant factor influencing σ. DWM@SP exhibits the highest σ value under these conditions, attributed to its optimized crosslinked network structure, which maintains a high effective carrier concentration and fast ionic conduction at high temperatures, while DOPO@SP may be limited by component dissolution loss or restrictions imposed by a high activation energy network.

First, the charge‐discharge capacity of LFP//Li button batteries with different modified polymer separators and PP separators (i.e., LFP/SP@PP/Li, LFP/DOPO@SP/Li, and LFP/DWM@SP/Li) was measured at 60 °C under different current densities (Figures S29–S32, Supporting Information). At a temperature of 60 °C, lithium batteries assembled from PP separator and SP@PP exhibit capacities below 100 mAh g^−1^ at a current density of 0.1C. In contrast, the LFP/DWM@SP/Li battery exhibited the highest capacity at 60 °C, reaching 158 mAh g^−1^ at 60 °C (Figure 4c). Additionally, the LFP/DOPO@SP/Li battery also exhibits satisfactory capacity at 60 °C, reaching 92 mAh g^−1^. To enhance comparison, the impact of different temperatures on the separator's charge–discharge capacity was tested by measuring the capacity retention rate during the temperature increase process from room temperature to high temperature at a current density of 0.5C. The lithium battery assembled with a PP separator and SP@PP exhibited significant fluctuations in capacity retention rate during the temperature increase process (Figure S33, Supporting Information). In contrast, the LFP/DWM@ SP/Li exhibited the highest capacity output in the temperature range from 60 to 130 °C, specifically 116, 125, and 126 mAh g^−1^ at 50, 60, and 90 °C, respectively. Additionally, the LFP/DOPO@SP/Li battery also demonstrated satisfactory capacity at 60 and 90 °C, with values of ≈75 and 78 mAh g^−1^, respectively. In summary, lithium metal batteries based on flame‐retardant‐modified polymer separators perform well across a wide range of temperatures from room temperature to high temperatures. The reversibility and activity of the electrochemical reactions occurring in LFP/Li batteries with different modified coated separators were evaluated using cyclic voltammetry (CV) (Figure 4d; Figure S34, Supporting Information). The oxidation and reduction peak potentials for all batteries were ≈3.7 and 3.25 V, respectively, corresponding to delithiation and lithiation at the cathode. As the coating interface changed, the peak potentials shifted slightly, which may be related to interface structure and polarization. However, the peak potential shifts gradually decreased, indicating the formation of a stable interface. Additionally, the LFP/DWM@SP/Li battery exhibits excellent rate performance at both 60 and 90 °C (Figures S35–S40, Supporting Information), particularly at 90 °C, where the reversible capacities at current densities of 0.1C, 0.2C, 0.5C, 1C, 2C, and 3C, the reversible capacities are 157.6, 148.5, 131.2, 117.6, 98.8, and 89.6 mAh g^−1^, respectively (Figure 4e). LFP//Li batteries based on LPE with different separators were subjected to long‐term cycling at 60 °C and 0.2C. The battery with the DWM@SP‐modified separator exhibited a coulombic efficiency (CE) of ≈100% (Figure 4f); this confirms the broad applicability of the modified separator. However, after a comprehensive comparison of capacity and stability, the LFP/DWM@SP/Li battery with a DWM flame‐retardant coating stood out. As expected, this battery achieved ultra‐long stable cycling time, extremely high initial capacity, and average CE. Specifically, the LFP/DWM@SP/Li battery was cycled 500 times at 0.5C under 90 °C conditions, achieving an initial discharge specific capacity of up to 150 mAh g^−1^, a discharge retention capacity of 130 mAh g^−1^, and an average CE exceeding 98.9% (Figure 4g; Figures S41 and S42, Supporting Information). More surprisingly, the LFP/DWM@SP/Li battery exhibited a high initial capacity of 117 mAh g^−1^ and an excellent capacity retention rate of 96.8% after 100 cycles at 2C and 130 °C, with an average CE value exceeding 98.2%, while the battery containing DOPO@SP separator (i.e., LFP/DOPO@ SP/Li battery) exhibited severe capacity loss (Figure 4h). Additionally, even after prolonged cycling, the battery maintained a capacity of ≈125 mAh g^−1^ and an average capacity retention rate of over 96% after 1000 cycles at a current density of 1C and 90 °C (Figure 4i). Overall, compared to state‐of‐the‐art polymer separator electrolytes, the modified flame‐retardant separator endows lithium metal batteries with unprecedented advantages in terms of capacity, rate capability, operating temperature, and lifespan (Figure 4k).^[^ ^74^ ^]^

Further investigation of the EIS of lithium‐ion batteries with different separators revealed that the charge transfer resistance (*R*_ct_) exhibits a semicircular shape in the mid‐to‐high frequency range (Figure S47, Supporting Information). The *R*_ct_ of lithium‐ion batteries using PP separators was 281.8 Ω, which decreased to 200.9 Ω after the addition of an SP coating. This reduction is attributed to the high conductivity of the SP coating, which effectively shortens the electron transport path. Upon introducing DWM, *R*_ct_ further decreased to 72.7 Ω, indicating that the DWM@SP coating plays a dual role in promoting both electron and ion transport. Notably, DOPO@SP coating‐modified membrane *R*_ct_ 149.9 Ω, which is higher than the SP coating but lower than the DWM@SP coating. This is because DOPO@SP has higher ionic conductivity, but the deposited DOPO reduces the electronic conductivity of SP. By measuring the electronic conductivity of the coating surface using a four‐probe test, the electronic conductivity of the SP coating was found to be 2.5 S cm^−1^, while the introduction of DWM caused only a slight decrease to 2.4 S cm^−1^. In contrast, after introducing DOPO, the electronic conductivity dropped to 2.0 S cm^−1^, indicating that the DOPO@SP coating has a negative impact on electronic transport, resulting in a higher *R*_ct_ value compared to DWM@SP (Tables S3 and S4, Supporting Information).

To further investigate the modification of the separator by the DWM cross‐linked network, the four separators were tested for tensile stress under similar working conditions. Notably, all separators exhibited reduced stress, but the DWM@SP separator still demonstrated a stress of up to 210 MPa even after high‐temperature operation at 130 °C. The DOPO@SP separator performed slightly below DWM but still exhibited a stress of 160 Mpa (Figure S48, Supporting Information). Additionally, the interfacial bonding energy between the coating and PP was calculated at different temperatures, revealing that the interfacial bonding energy between the DWM‐modified coating and PP remained high even after high‐temperature treatment (Figure S49, Supporting Information). This demonstrates that the DWM flame retardant can withstand high‐intensity operation while maintaining good mechanical properties. Additionally, DSC testing was conducted on the membranes after exposure to different temperatures. Notably, while the DWM@SP membrane did not alter the melting temperature of the PP membrane, it exhibited a lower melting temperature area after exposure to 130 °C compared to after exposure to 90 °C (Figure S50, Supporting Information). To verify this phenomenon, we conducted SEM observations and FTIR tests on the coatings after high‐temperature testing. The SEM images showed that the SP coating exhibited larger particles and agglomeration compared to before testing, while the DOPO@SP coating did not exhibit larger particles but showed many DOPO particles adhering to SP particles, forming agglomerates. This result may explain the stagnation observed in the DOPO@SP coating during high‐temperature charge–discharge testing at 90 and 130 °C. Surprisingly, the DWM@SP coating exhibited some adhesion but maintained a good shape, providing assurance for the modified separator to withstand long‐term high‐temperature charge–discharge cycles (Figure S51, Supporting Information).^[^ ^75^ ^]^

To enhance comparison, different separators were treated at temperatures higher than the test temperature, and the resulting FTIR images are shown in Figure S52 (Supporting Information). Since the main structural peaks of DOPO and DWM did not show significant changes, we selected unsaturated olefins in SP as the observation target (3450 cm^−1^). The unsaturated olefins in the SP coating showed a significant decrease, which is likely to result in their release during charge‐discharge testing, which is detrimental to battery performance. The unsaturated olefins in DOPO@SP also decreased, but the change was much smaller compared to SP. Notably, the unsaturated olefins in the DWM@SP coating remained almost unchanged, demonstrating that DWM's strong dipole interaction can firmly lock the unsaturated olefins, preventing internal short circuits or other undesirable behaviors in the battery.^[^ ^76^, ^77^ ^]^

### 2.3 Morphology and Chemistry at the Interphase

**Figure 5:**
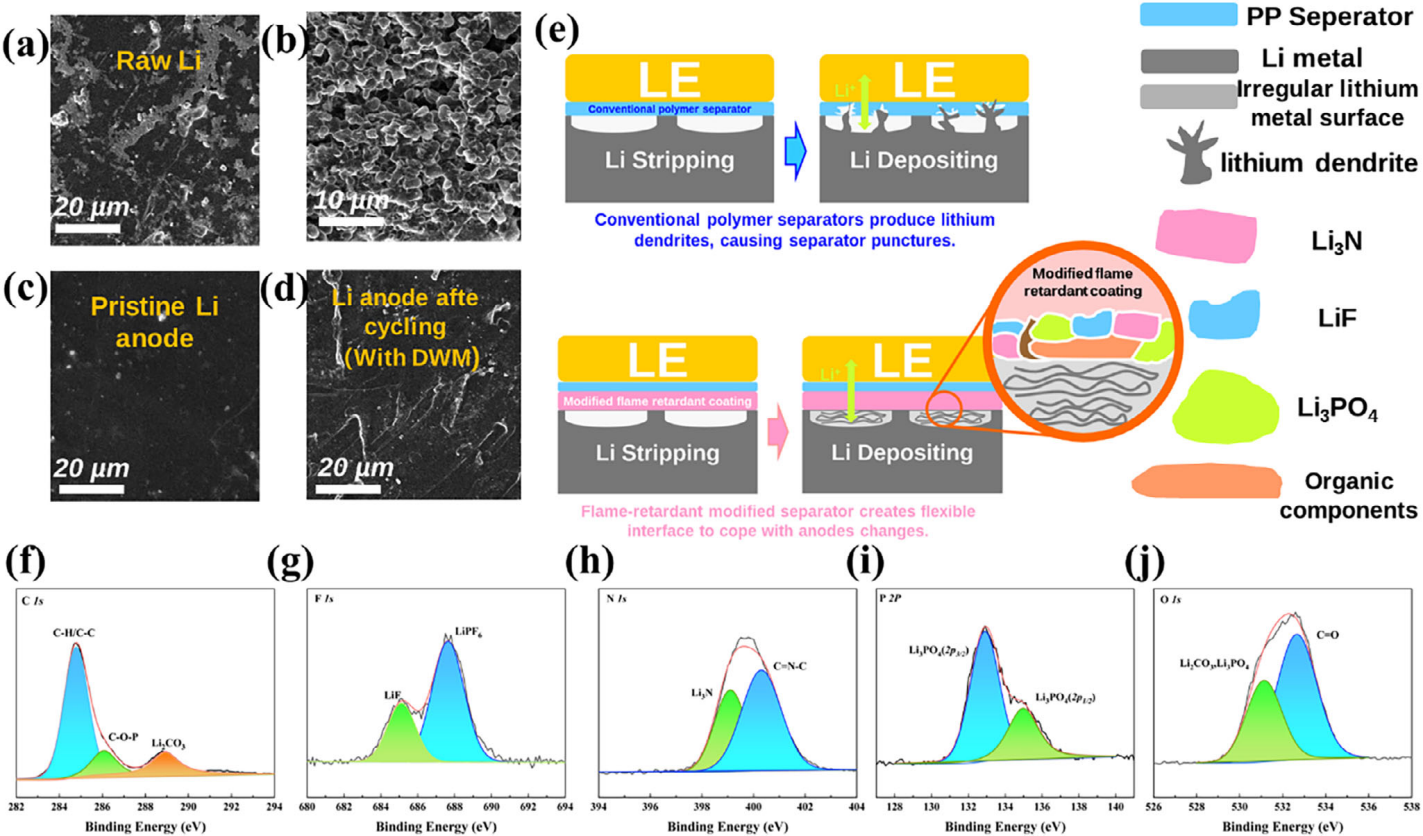
a,b) SEM images of unmodified lithium foil; c,d) SEM images of the lithium anode before and after cycling in the DWM‐assembled Li/Li battery; e) schematic diagram of lithium dendrites on a conventional polymer separator and a separator with a modified flame‐retardant coating; the figure below shows the XPS elemental energy spectrum: f) C 1s, g) F 1s, h) N 1s i) P 2p j) O 1s.

There is no doubt that lithium metal batteries based on LPE must have a stable interface and good interface contact to achieve excellent electrochemical performance. The battery was disassembled after 1000 cycles to observe the interface morphology between the modified separator and the electrode. Figure S28 (Supporting Information) shows that the introduction of the modified flame‐retardant coating improved the contact between the electrolyte and the cathode interface. On the other hand, the abundant O and N atoms in DWM provide excellent affinity for lithium metal. Therefore, a dense and complete interface layer was observed on the lithium metal (**Figure** **5**a,b). The thickness of the lithium deposition layer was ≈45 µm, with no lithium dendrites. In contrast, the unmodified DOPO coating was filled with dense lithium dendrites.

After the polymer components were completely washed off from the anode surface, a smooth and dense surface was exposed (Figure 5c), similar to the original lithium metal anode (Figure 5c; Figures S54 and S55, Supporting Information). Additionally, XPS spectra confirmed the chemical composition of the interface. As shown in the Figure 5h, in the N 1s fine spectrum of DWM after cycling, the N‐C peak and C = N peak appeared at 399.4 and 398.3 eV, respectively. In the O 1s fine spectrum of DWM (Figure 5j), the P = O peak at 534.5 eV, the P‐O‐C peak at 533.7 eV, and the C‐O/C‐OH peak at 532.3 eV of DOPO can be observed. After the adsorption reaction with LiF, the characteristic peaks of the O and N‐related groups in DWM shift significantly toward lower binding energies, which can be attributed to the transfer of lone pair electrons from N and O to the terminal lithium atoms of electron‐deficient LiF. Additionally, new characteristic peaks of P─F bonds appear at 131.6 eV in the P 2p fine spectrum (Figure 5i), which is caused by the nucleophilic substitution reaction between the P‐O groups in DWM and LiF. The above analysis indicates that DWM effectively adsorbs LiF through a dual chemical adsorption mechanism, thereby limiting the concentration diffusion of LiF. To further elucidate the interaction mechanism between DWM and PF_6_, FTIR curves of the electrolyte before and after adding DWM were included for comparative analysis. This is primarily due to the influence of the phosphoramidite groups and oxygen‐containing groups in DWM. The strongly electron‐withdrawing P‐O groups exert an attractive force on the anionic center of PF_6_^−1^, while hydrogen bonding interactions also exist between the oxygen‐containing groups and PF_6_^−1^. Within lithium‐ion batteries, in addition to Li^+^ ion migration, the migration of lithium salt counterions typically accounts for a significant proportion, a phenomenon that exacerbates concentration polarization, leading to uneven distribution of Li^+^ at the electrode‐electrolyte interface. By combining steady‐state polarization methods and electrochemical impedance spectroscopy (EIS), the t_Li_^+^ value of the electrolyte can be measured. As shown in the figure, when using a PP separator, the t_Li_^+^ value of the electrolyte is only 0.16, indicating that the migration of the counterion PF_6_^−1^ accounts for a high proportion. In contrast, in the DWM@SP coating, the interaction between DWM and PF_6_^−1^ restricts the migration of PF_6_^−1^, increasing the t_Li_^+^ value to 0.47, which helps alleviate the issues of uneven Li^+^ distribution and lithium dendrite formation (Figure S53, Supporting Information).

It can be concluded that the abundant LiF, Li_3_N, and Li_3_PO_4_ in lithium batteries assembled with modified flame‐retardant coatings confer excellent thermal stability, mechanical strength, ionic conductivity, and flexibility to the interface, enabling LMBs to maintain long‐term cycling stability even at high temperatures. Lithium metal anodes undergo significant volume changes during charge‐discharge cycles, necessitating a stable barrier structure from the separator to maintain good interface contact and integrity. Poor interface contact and resulting defects can lead to uneven charge distribution, interface layer damage and thickening, and lithium dendrite growth, which are primary causes of reduced battery lifespan. The lower mechanical properties of conventional polymer separators cannot address these issues. In contrast, the addition of modified coatings enables the separator to maintain normal barrier function and good mechanical properties at high temperatures without affecting electrolyte flow, forming a complete and continuous flexible interface layer composed of inorganic and organic composite components (Figure 5e).

### 2.4 All‐Around Safety of Li Metal Batteries

**Figure 6:**
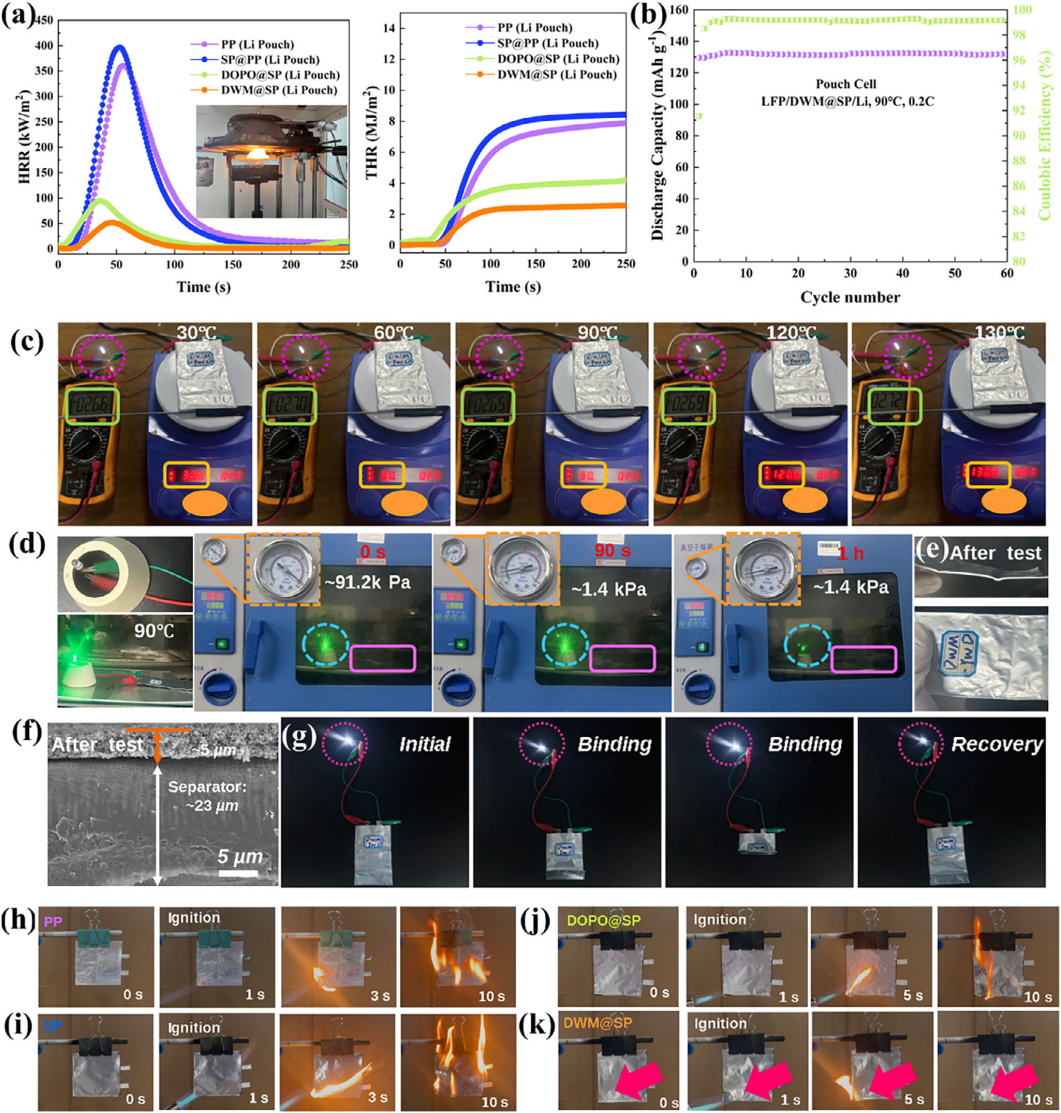
a) Thermal release curves and total thermal release curves of soft‐pack batteries with different separator assemblies measured using a micro‐cone calorimeter b) Long‐cycle performance of soft‐pack batteries with DWM‐modified separator assemblies at 90 °C and 0.2C c) Abuse test of soft‐pack batteries assembled with DWM‐modified separators on a heating platform and d) abuse test in a high‐temperature, low‐pressure environment. e) SEM cross‐sectional image of the separator after abuse and a light bulb illumination test of the abused soft‐pack battery. f,g) Combustion open tests of soft‐pack batteries assembled with four types of separators.

Due to the excellent thermal stability and superior flame retardancy of the modified flame‐retardant coating polymer separator, a cone calorimeter was used to study the thermal runaway behavior of pouch batteries assembled with different separators and LE (**Figure** **6**a). The temperature display of soft‐pack batteries monitored by thermocouples indicates that, when coated with DWM@SP, the battery's heat release rate(HRR) is significantly reduced to 45 kW m^2^, with total heat release(THR) remaining below 2 MJ m^2^. This prevents flame propagation between battery cells, substantially enhancing the fire safety of lithium‐ion batteries. The thermal release rates of soft‐pack batteries assembled with DOPO@SP also showed satisfactory results. However, we found that the thermal release rate of batteries assembled with SP@PP was higher than that of pure PP, possibly due to the decomposition of conductive carbon during combustion, leading to increased thermal release rates. To verify the practical commercial application potential of the modified flame‐retardant polymer separator, we fabricated pouch batteries for testing their cycling performance and safety under harsh conditions.

As shown in Figure 6b, the LFP/DWM@SP/lithium pouch battery exhibits a discharge specific capacity exceeding 140 mAh g^−1^ at 0.2C and 90 °C, with an average coulombic efficiency exceeding 99.2%. It is foreseeable that LMBs used in high‐temperature environments will face more severe safety risks, including sudden changes in external temperature and internal heat accumulation. To verify whether LMBs based on Modified separator are safer, we set up a simple experimental apparatus to test the safety of pouch batteries during thermal abuse processes (Figure 6c; Figure S56, Supporting Information). The green LED remained lit continuously within the temperature range of 30 to 130 °C, with stable operating voltage (Figure S57, Supporting Information), and the battery did not swell or deform during the test. To further validate safety, the pouch battery was placed in a vacuum oven at 90 °C to simulate potential swelling in high‐temperature environments or high‐altitude regions. For Li pouch batteries, the PP unable to withstand this condition caused significant swelling of the battery and failure under vacuum conditions (Figure S58, Supporting Information). In contrast, the DWM@PP‐modified separator with a stable structural coating demonstrated excellent stability, enabling the pouch battery based on the modified separator to operate for over 1 h at 90 °C and 1.4 kPa, with the appearance remaining intact after testing (Figure 6d), and the SEM image of DWM@SP after testing also verified this point (Figure 6e,f). Additionally, pouch batteries using modified flame‐retardant coated polymer separators exhibit excellent flexibility and high resistance to mechanical damage. During folding and cutting, the battery remained capable of illuminating an LED (Figure 6g). To further highlight the fire safety of LMBs based on modified flame‐retardant coatings, combustion tests were also conducted on the pouch batteries. Pouch batteries based on PP separators were rapidly ignited within 0.5 s and continued to burn with intense flames for 15 s (Figure 6h–k). However, the pouch battery based on the modified flame‐retardant coating polymer separator extinguished quickly after ignition, although the battery surface retained burn marks, it did not exhibit severe combustion behavior, indicating that the non‐flammable flame‐retardant coating significantly enhances the fire safety of LMB. These results indicate that under various harsh environments or abusive conditions, the modified flame‐retardant coating will confer outstanding performance and extremely high safety to practical LMBs.

## 3 Discussion

This study has successfully developed a functionalized coating separator that combines high ionic conductivity with inherent flame‐retardant properties, providing an innovative solution for realizing high‐safety, high‐performance lithium metal batteries (LMBs). This coating employs a covalently crosslinked network formed by phosphorus‐based flame retardants and melamine as its matrix, systematically addressing multiple critical challenges currently facing lithium metal batteries. These include inadequate mechanical properties of separators, uncontrolled lithium dendrite growth, limited thermal stability, sluggish lithium deposition/stripping kinetics, severe electrode–electrolyte interface side reactions, and prominent safety risks.

Crucially, the abundant polar functional groups within this flame‐retardant coating (e.g., P = O, C = N) exert potent dipole‐dipole interactions. These enable directed adsorption of electrolyte anions and promote lithium salt dissociation, while simultaneously guiding uniform lithium‐ion transport. This significantly enhances stability at the lithium‐metal interface. This mechanism not only suppresses lithium dendrite formation and growth but also promotes the in situ formation of a robust, LiF‐rich solid electrolyte interphase (SEI). Consequently, highly reversible lithium deposition/stripping behavior is achieved in the full cell, substantially enhancing electrochemical compatibility and long‐cycle stability.

Across a broad temperature range (60–130 °C), this functional separator exhibits outstanding electrochemical performance: lithium symmetric cells achieve over 1000 h of cycle life, while LFP//Li full cells maintain high coulombic efficiency (>99%) and excellent capacity retention (>80%) during long‐term cycling. Concurrently, the separator exhibits outstanding thermal stability and mechanical strength, effectively suppressing thermal runaway propagation while maintaining structural integrity and safe operation under extreme mechanical abuse conditions.

The novel covalently crosslinked flame‐retardant coating architecture proposed in this study not only provides a practical solution to the current safety bottlenecks in lithium metal batteries but also demonstrates a material design strategy achieving multifunctional integration through molecular interface regulation. This achievement lays a robust scientific foundation and charts a new technological pathway for developing next‐generation high‐energy battery systems capable of withstanding extreme operating conditions while possessing intrinsic safety and extended longevity.

## 4 Experimental Section

All reagents were used directly without purification. 9,10‐Dihydro‐9‐oxa‐10‐phosphaphenanthrene‐10‐oxide (DOPO), melamine, and glutaraldehyde (C_5_H_8_O_2_) were purchased from McLean (Shanghai). Lithium hexafluorophosphate (LiPF_6_), conductive carbon (SP), ethylene carbonate (EC) (99.5%), and dimethyl carbonate (DEC) (99.5%) were purchased from Kolude.

### Fabrication of the Separator

The PP separator were manufactured by extruding polypropylene masterbatch sourced from Sinopec Maoming Branch through a twin‐screw extruder, followed by film blowing. The twin‐screw extruder and film blowing machine models are SJZS‐10A and SCM20 respectively (Ruiming, Wuhan). The twin‐screw extruder operates at 25 rpm, while the film thickness is set according to the standard of commercially available Celgard 2500. Other modified membranes are produced by directly coating the PP membrane using a coating machine after mixing.

### Synthesis of DWM

A certain molar mass of DOPO and glutaraldehyde were added to 80 ml of acetone, heated to 80 °C, and reacted for 5 h. Then, a certain molar mass of melamine was added and reacted at 60 °C for 6 h. The three reactants had a specific molar mass ratio.

### Preparation of DWM@PP Modified Separator

DWM@SP coating was prepared using an expandable slurry coating method. The prepared DWM, SP, and polyvinylidene fluoride (PVDF) binder were mixed in a 4:4:2 mass ratio in the solvent N‐methylpyrrolidone for 24 h to obtain a uniform slurry. The slurry was coated onto a PP separator to obtain the DWM@SP modified separator. DOPO@SP modified separators were prepared using the same method and ratio, with DOPO replacing DWM. Using the same method, a slurry composed of 80 wt.% SP and 20 wt.% PVDF was coated to prepare SP modified separators. The mass loading of all coatings was controlled to ≈0.3 mg cm^−2^. All modified membranes were placed in a vacuum oven at 60 °C for 24 h to remove the solvent.

### Battery Electrode Preparation and Battery Assembly

The electrodes were assembled into CR2032 button batteries in a glove box filled with Ar, with oxygen and moisture concentrations of 0.01 ppm. The negative electrode directly uses a metal lithium sheet as the negative electrode. LiFPO_4_ (LFP) is purchased from the market. The preparation method for the positive electrode involves casting the slurry onto an aluminum foil. The slurry consists of active material (LFP), ultra‐P, and poly(vinylidene fluoride) (PVDF) in a weight ratio of 80:10:10. The anode particles have a diameter of 12 mm. After complete drying at 80 °C for 12 h, the active mass loading is ≈3 mg cm^−2^. For the CR2032 coin cell assembly, the battery is assembled using a metal lithium foil and commercial LFP as the anode and cathode, respectively. The electrolyte consists of a solution of 1 M LiPF6 in ethylene carbonate (EC)/diethyl carbonate (DEC) (1:1 v/v).

### Density Functional Theory (DFT) Calculations

All molecular simulation calculations in this study were performed using the Materials Studio (2023) software platform.^[^ ^78^, ^79^ ^]^ Quantum mechanical calculations were conducted using the Dmol^3^ module. In this study, molecular dynamics (MD) methods were systematically employed on the Material Studio 2023 platform to investigate mixtures of polypropylene (PP) or its modified separators (SP@PP, DOPO@SP, DWM@SP) with LiPF_6_/EC:DEC (1:1 v:v) electrolyte at specific ratios. Initially, a periodic amorphous initial structure containing the aforementioned components was constructed. Subsequently, the system underwent energy minimization and molecular dynamics simulations under the NPT ensemble to achieve sufficient structural relaxation and equilibrium. After equilibrium was achieved, the system was subjected to extended‐duration MD simulations under the NVT ensemble at 333, 363, and 403 K (total duration of 2000 ps), with a time step of 1 fs. Temperature control was achieved using the velocity scaling thermostat. Van der Waals interactions were handled using an atom‐based summation method, while electrostatic interactions were addressed using the Ewald summation method with a precision of 1 × 10^−5^ kcal mol^−1^. Both methods employed a cutoff radius of 20.5 Å. Simulation trajectories were sampled every 2000 fs. All calculations were based on the COMPASS III force field. Finally, the Forcite module was used to perform mean square displacement (MSD) analysis on the equilibrium trajectory to obtain ion transport dynamics information. The diffusion coefficient (D) of the lithium ion was calculated using the MSD formula: $D=\lim_{t\to \infty }\frac{1}{6t}\;\left({\left|r(t)-r(0)\right|}^{2}\right)$ The dipole moment was calculated using the DMOL3 module, the adsorption energy was calculated using the Castep module, and the solvation free energy was obtained using the Forcite module.

## Conflict of Interest

The authors declare no conflict of interest.

## Author Contributions

W.Y. and W.T. contributed equally to this work. W.Y. performed data curation, formal analysis, investigation, methodology, resources, software, validation, and wrote the original draft. W.T. performed conceptualization, funding acquisition, resources, software, supervision, wrote, reviewed and edited. W.S. performed investigation and data curation. K.S. wrote, reviewed and edited, and funding acquisition. Q.X. performed data curation. Z.W. performed data curation, and investigation. X.L. performed data curation, and provided resources. J.M. performed conceptualization, investigation, and wrote the original draft. H.Y. performed conceptualization, formal analysis, and wrote the original draft. Z.P. performed conceptualization, formal analysis, and wrote the original draft.

## Supporting information



Supporting Information

## Data Availability

The data that support the findings of this study are available from the corresponding author upon reasonable request.
