# Supplementary material for: Trifunctional DOPO‐Engineered Polypropylene Separator With Li⁺‐Concentrating Interfaces for High‐Safety Lithium‐Ion Batteries Under Extreme Conditions
Source: Adv Sci (Weinh). 2025 Oct 14;13(2):e16139. doi: 10.1002/advs.202516139 (PMC12786285; doi:10.1002/advs.202516139)
Supplement: Supplementary file 1 — Supporting Information [file ADVS-13-e16139-s001.docx]

**Trifunctional DOPO-Engineered Polypropylene Separator with Li⁺-Concentrating Interfaces for High-Safety Lithium-Ion Batteries under Extreme Conditions**

*Wende Yi ^a, c, 1^, Wufei Tang ^a, c, 1, ^[[1]](#footnote-1)^*^, Weikang Su ^a^, Keren Shi ^c^, Qiaowei Xiao ^c^, Ziyan Wang ^c^, Xiaoyu Li ^c^, Jingyang Mu ^b,^[[2]](#footnote-2)^*^, Huiqin Yao ^b,^[[3]](#footnote-3)^*^, Zhihan Peng ^d, ^[[4]](#footnote-4)^*^*

*^a^ College of Chemistry and Bioengineering, Hunan University of Science and Engineering, Yongzhou 425199, China.*

*^b^ General Hospital of Ningxia Medical University, College of Basic Medicine, Ningxia Medical University, Yinchuan 750004, China.*

*^c^ State Key Laboratory of High-efficiency Utilization of Coal and Green Chemical Engineering, College of Chemistry & Chemical Engineering, Ningxia University, Yinchuan 750021, Ningxia, China.*

*^d^ College of Materials Science and Engineering, Donghua University, Shanghai 201620, China*


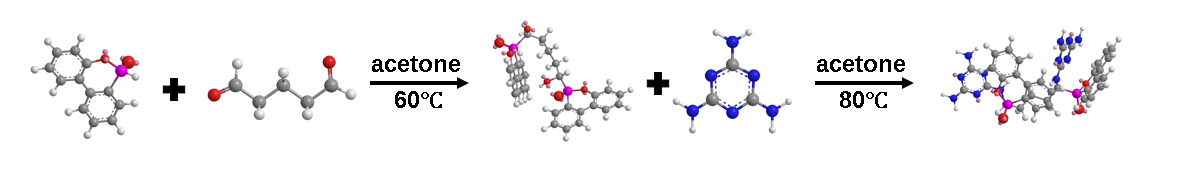


**Fig. S1.** DWM synthesis path map.

**
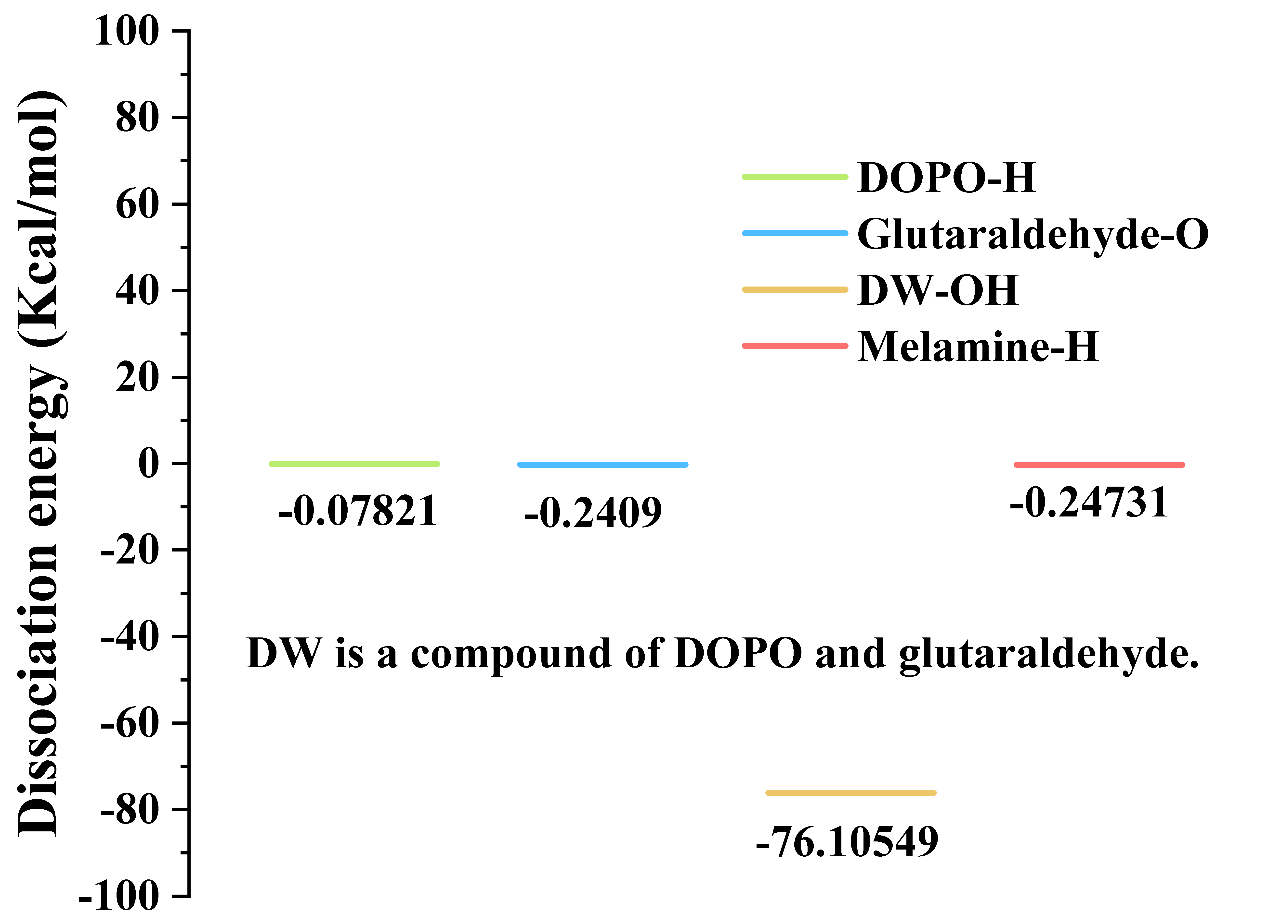
**

**Fig. S2.** DWM synthesis of dissociation energy at each step.

**
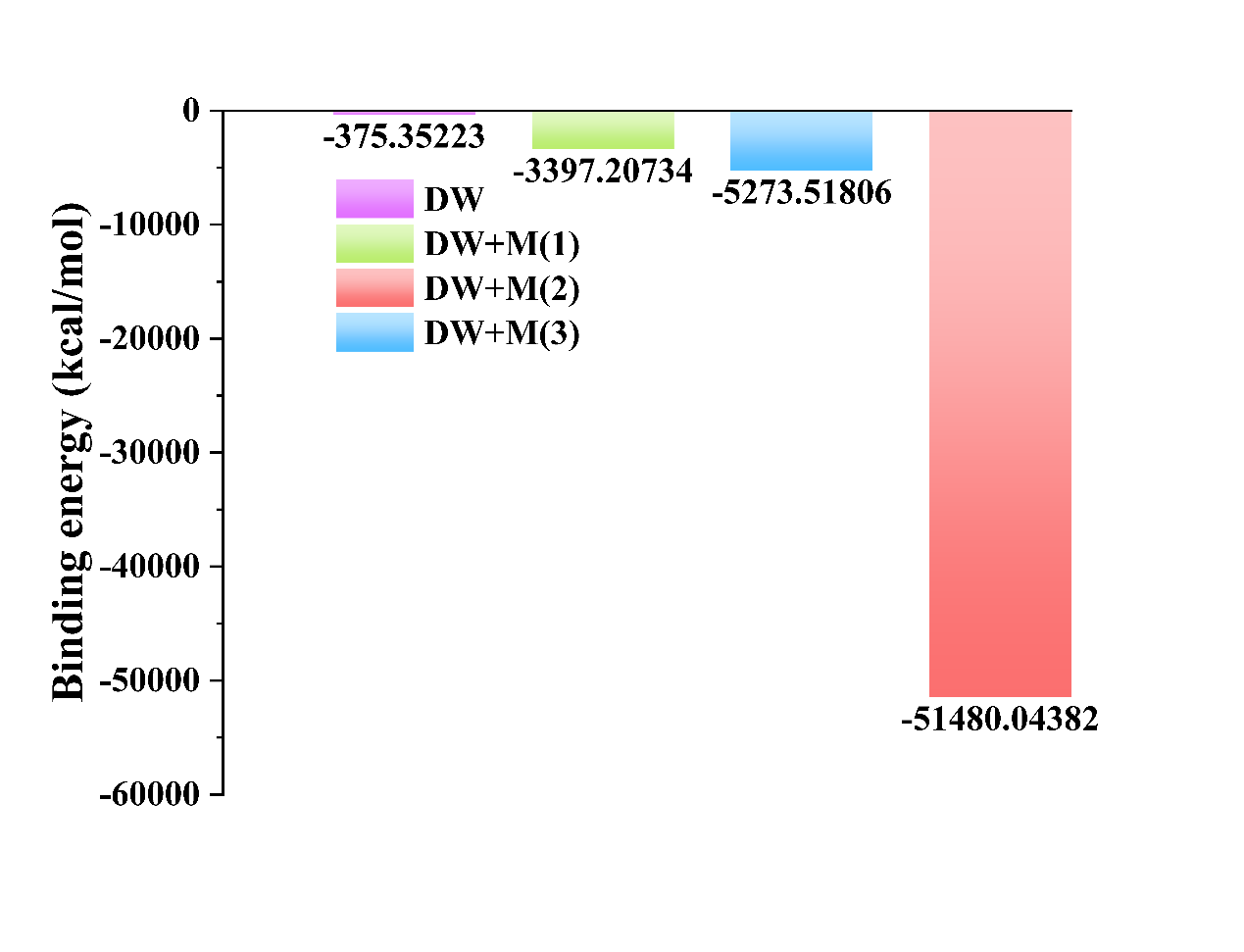
**

**Fig. S3.** DWM synthesis of the binding energy at each step.

**
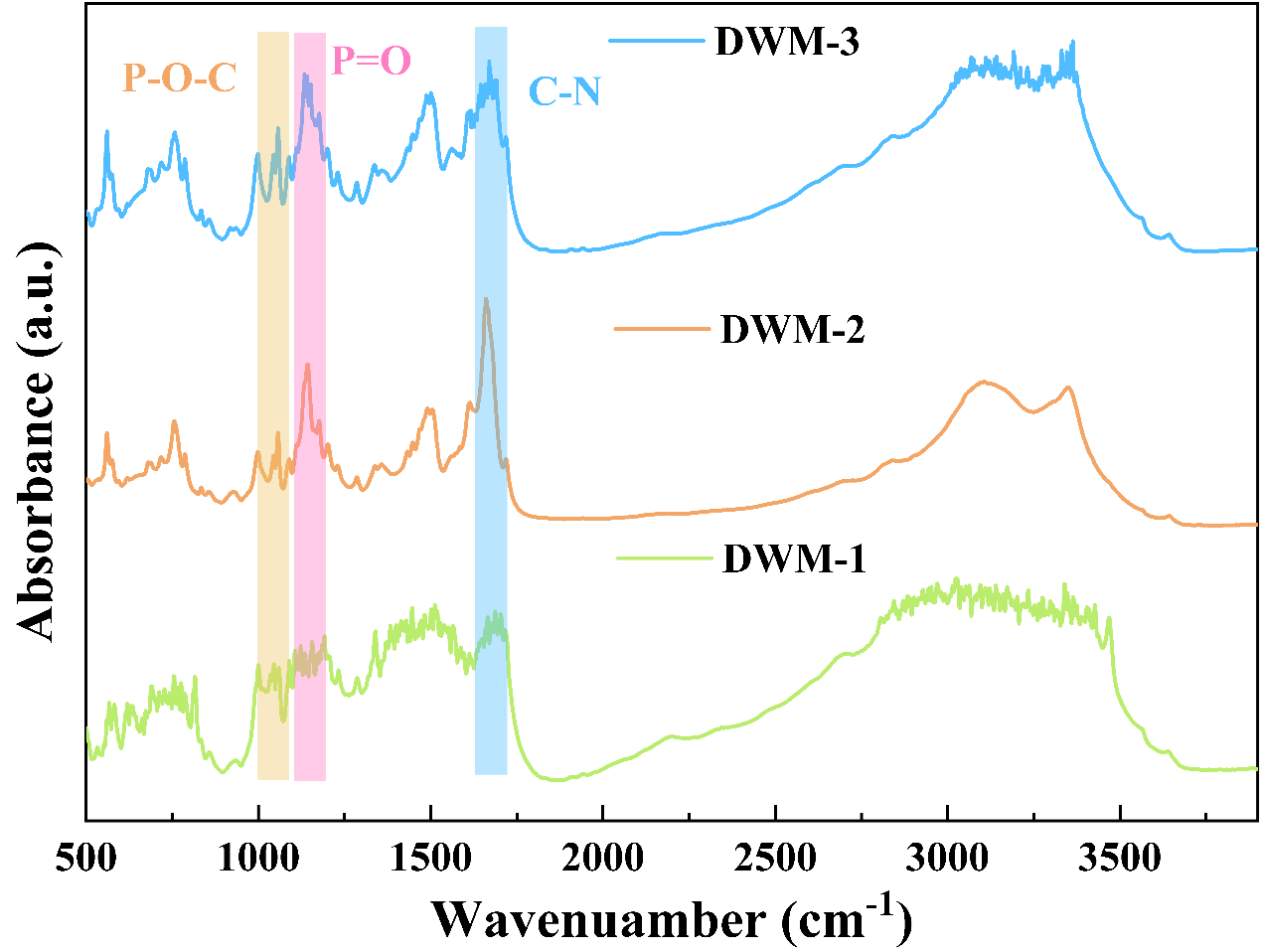
**

**Fig. S4.** FTIR Spectrum of DWM at different ratios.

**
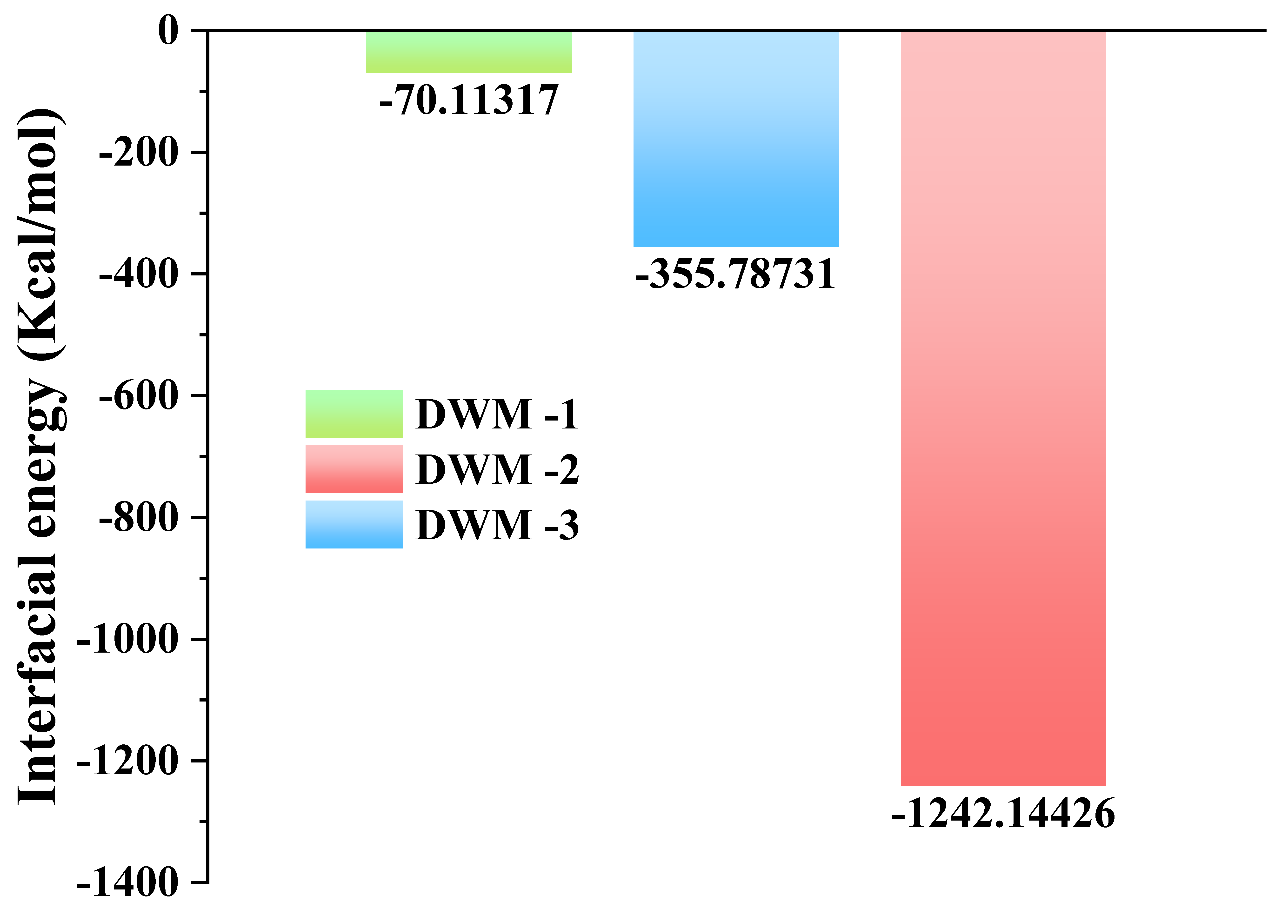
**

**Fig. S5.** Binding energy at the interface between DWM and PP membranes at different ratios.

**
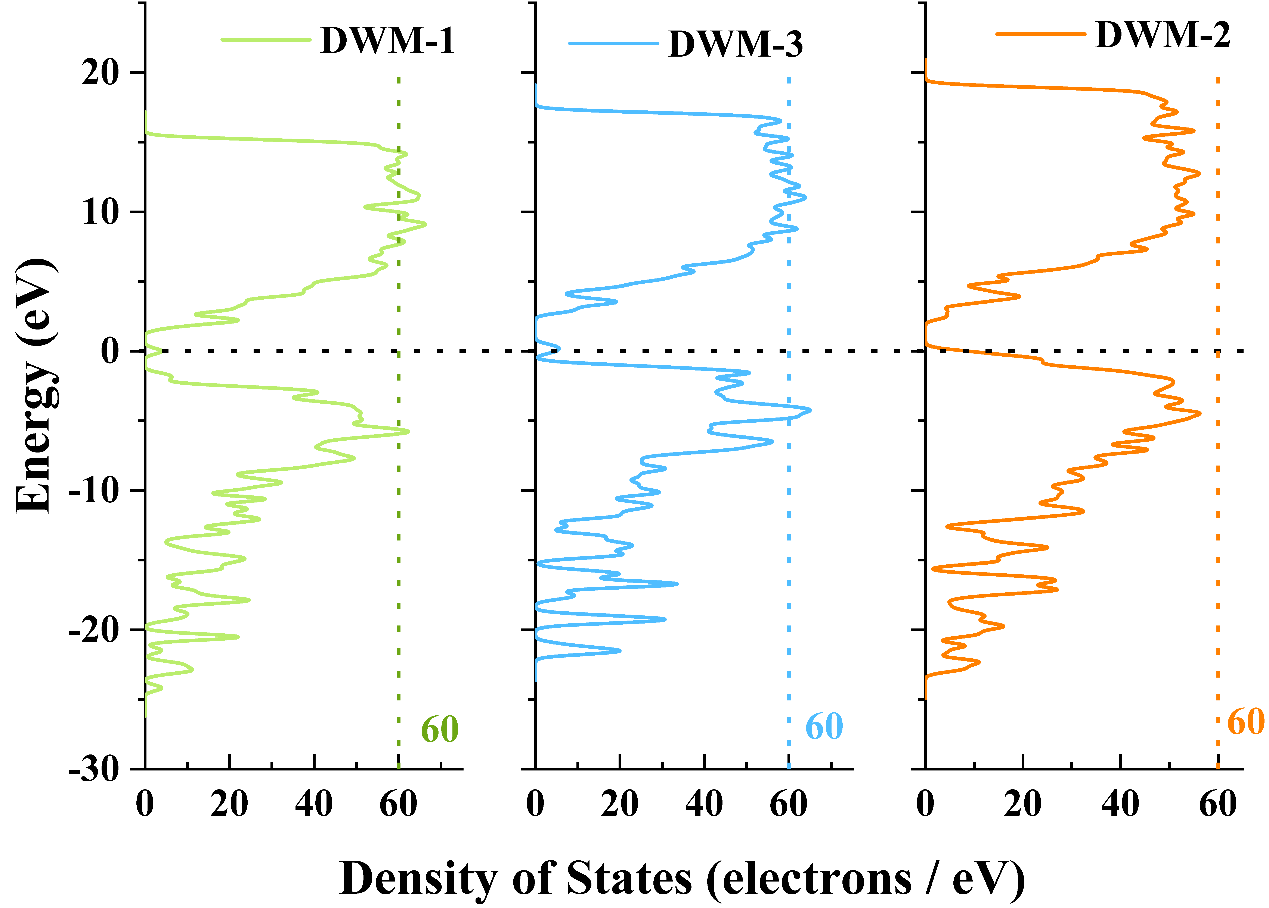
**

**Fig. S6.** State density of DWM at different ratios.


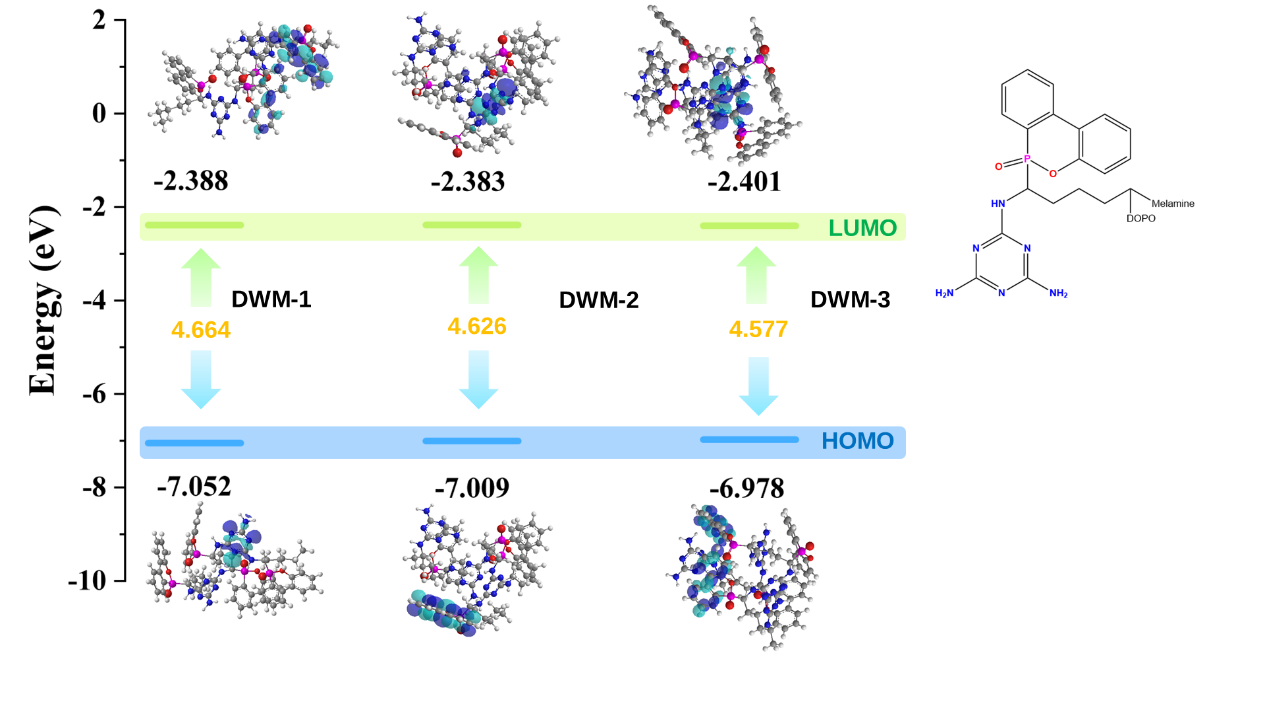


**Fig. S7.** The figure shows the energy levels of the highest occupied molecular orbital (HOMO) and lowest unoccupied molecular orbital (LUMO) of DWM at different ratios.

**
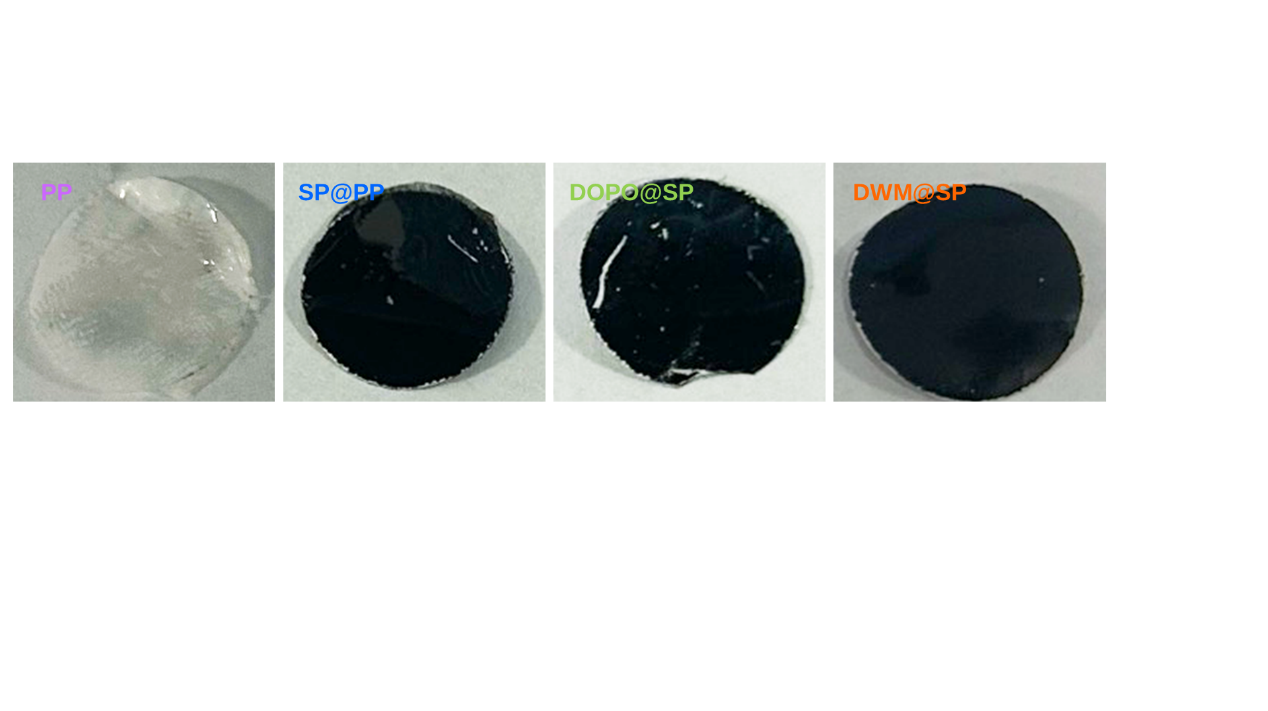
**

**Fig. S8.** Electrolyte wetting of different seperator.

**
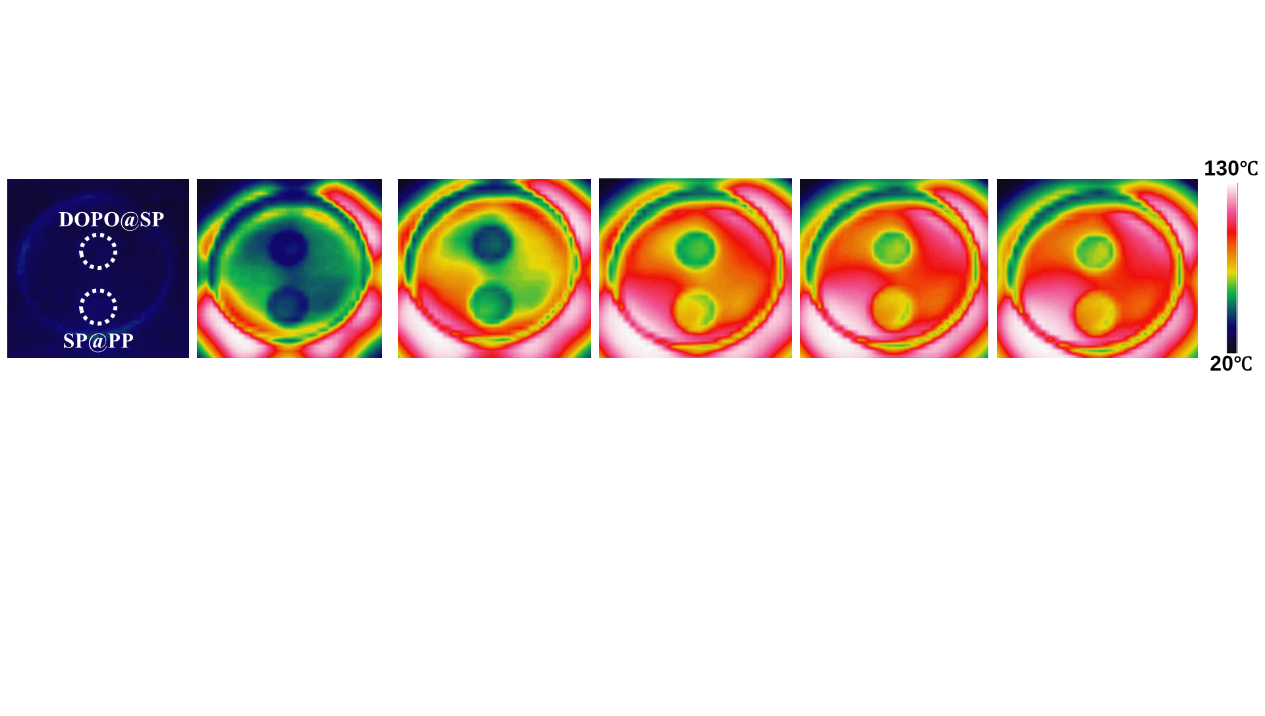
**

**Fig. S9.** Thermal imaging pictures of DOPO@SP and SP@PP.

**
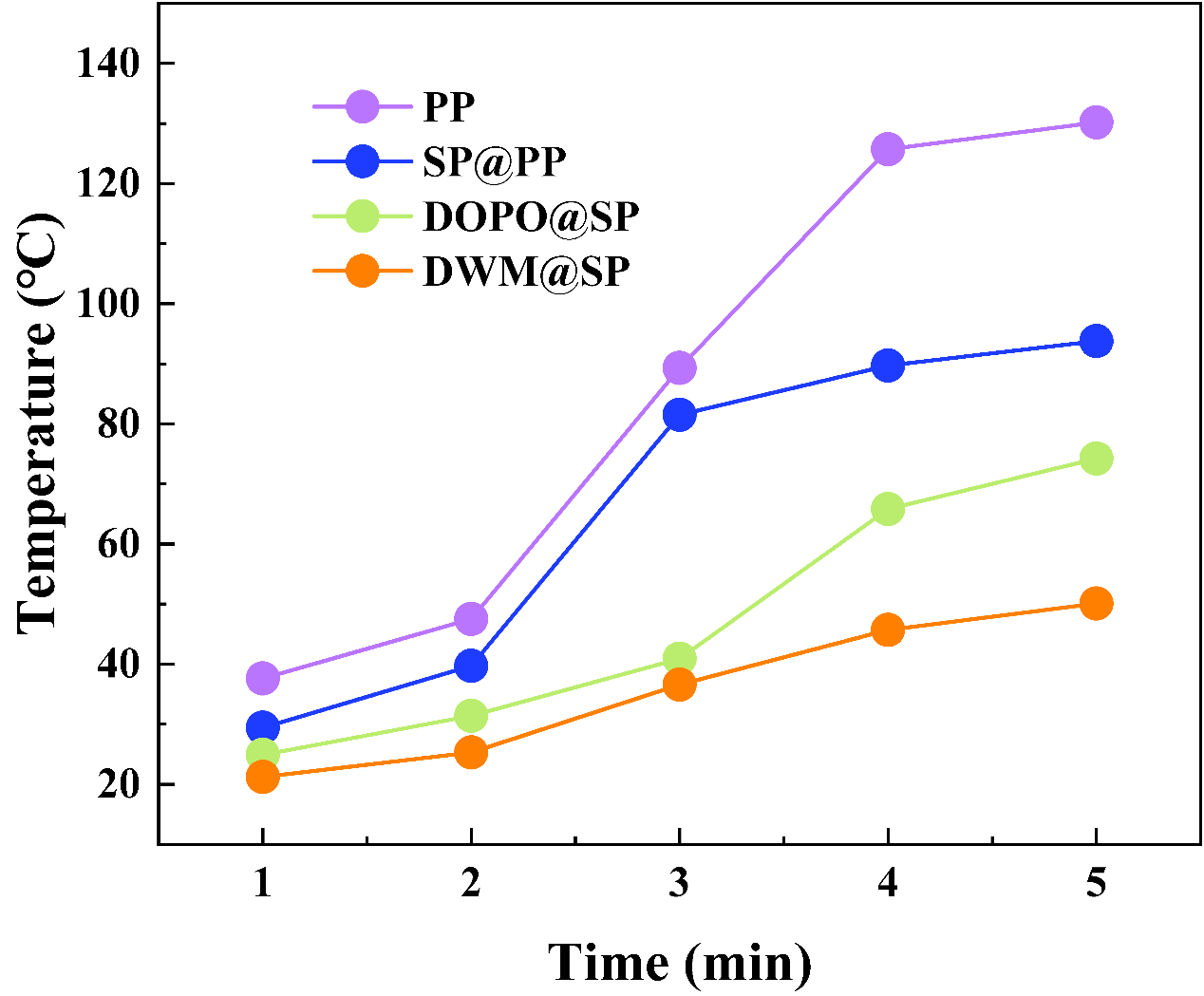
**

**Fig. S10.** Thermal imaging pictures of different membranes Heating process in temperature and time curves.


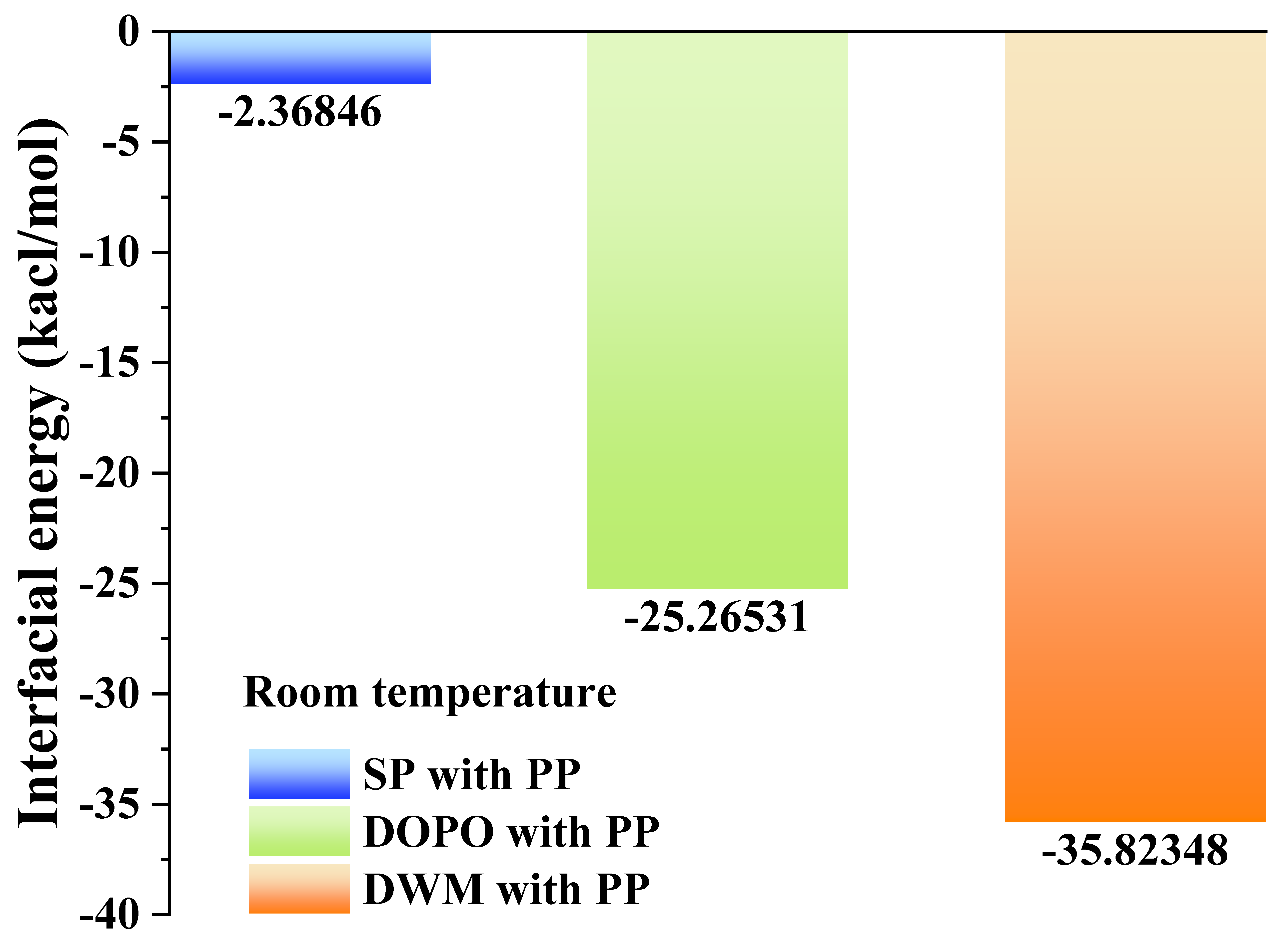


**Fig. S11.** Interfacial bonding energy between different coatings and PP.

**
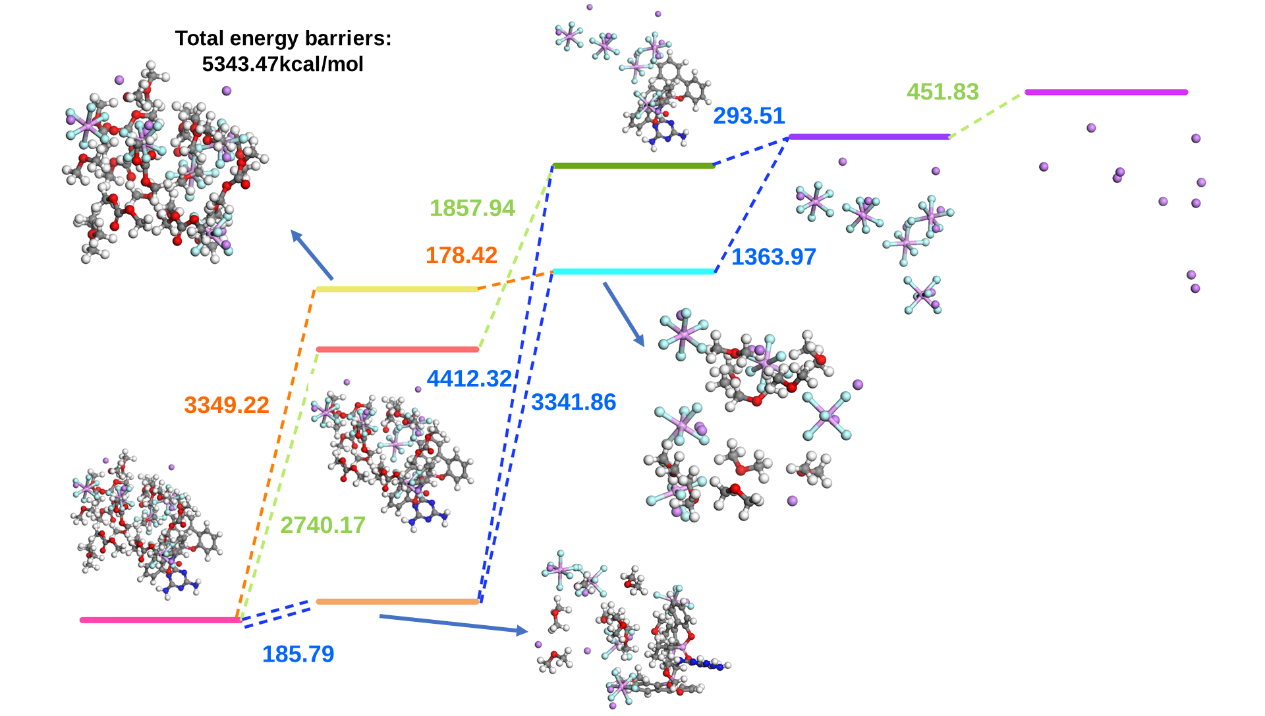
**

**Fig. S12.** Solubilisation free energy barrier diagram of DWM-1 material.


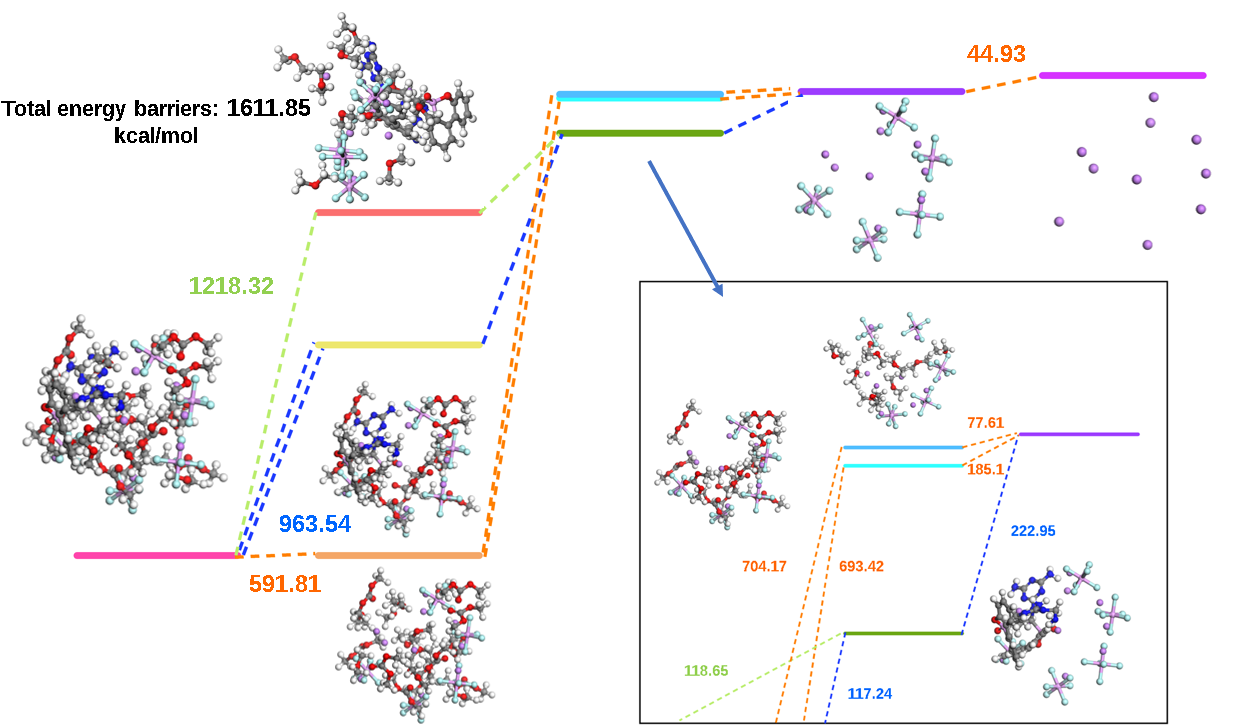


**Fig. S13.** Solubilisation free energy barrier diagram of DWM-3 material.


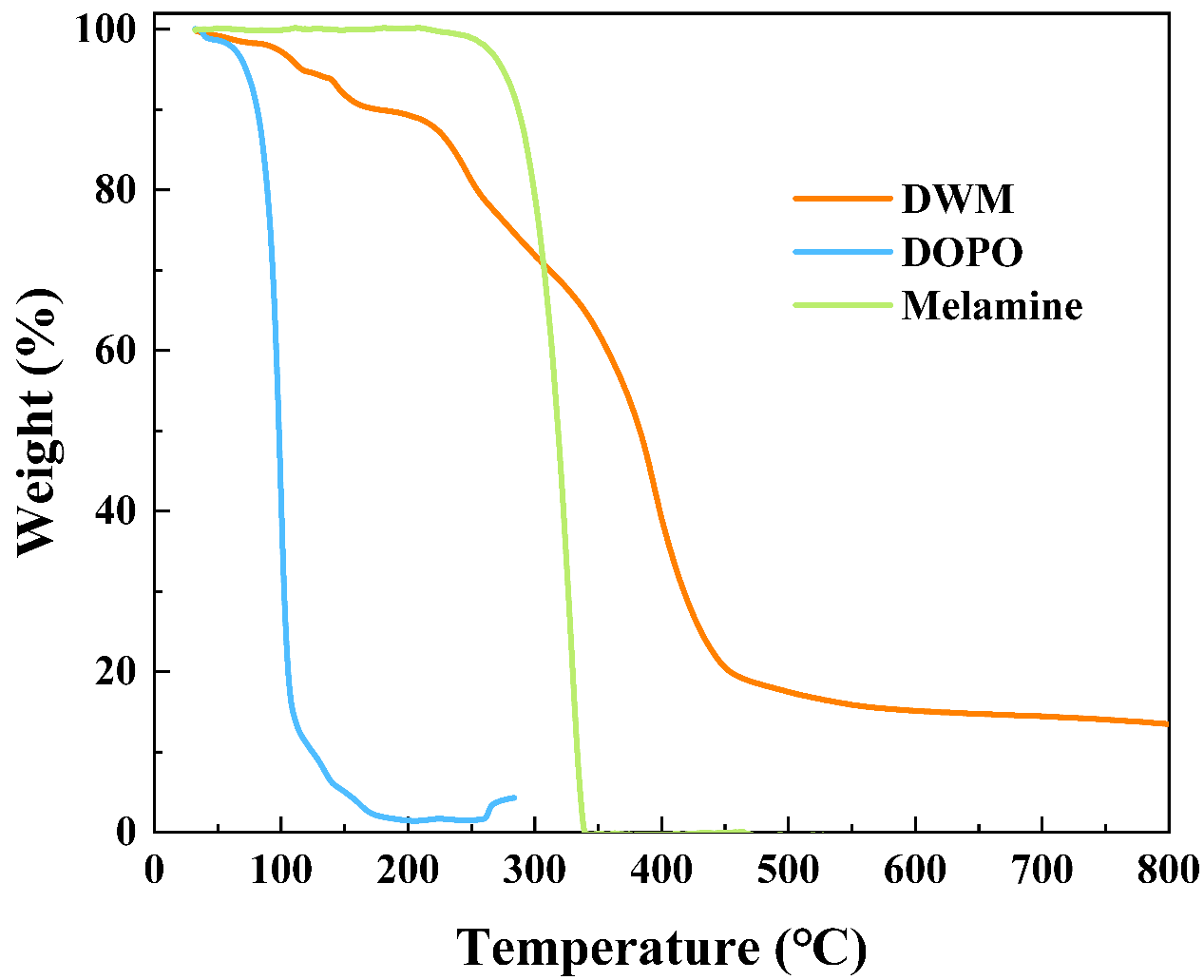


**Fig. S14.** TGA curves of materials


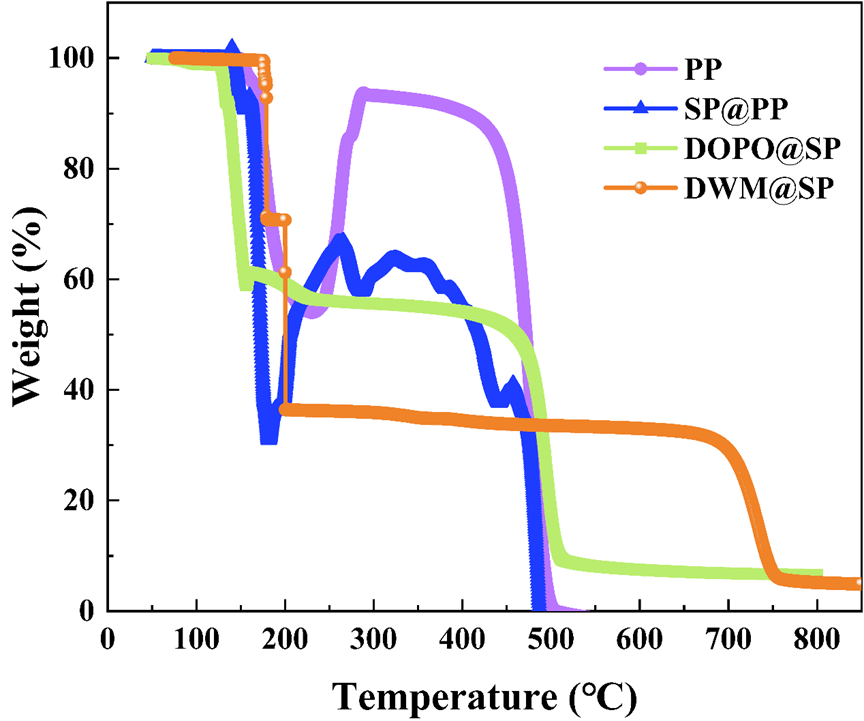


**Fig. S15.** TGA curves of different seperator.

**
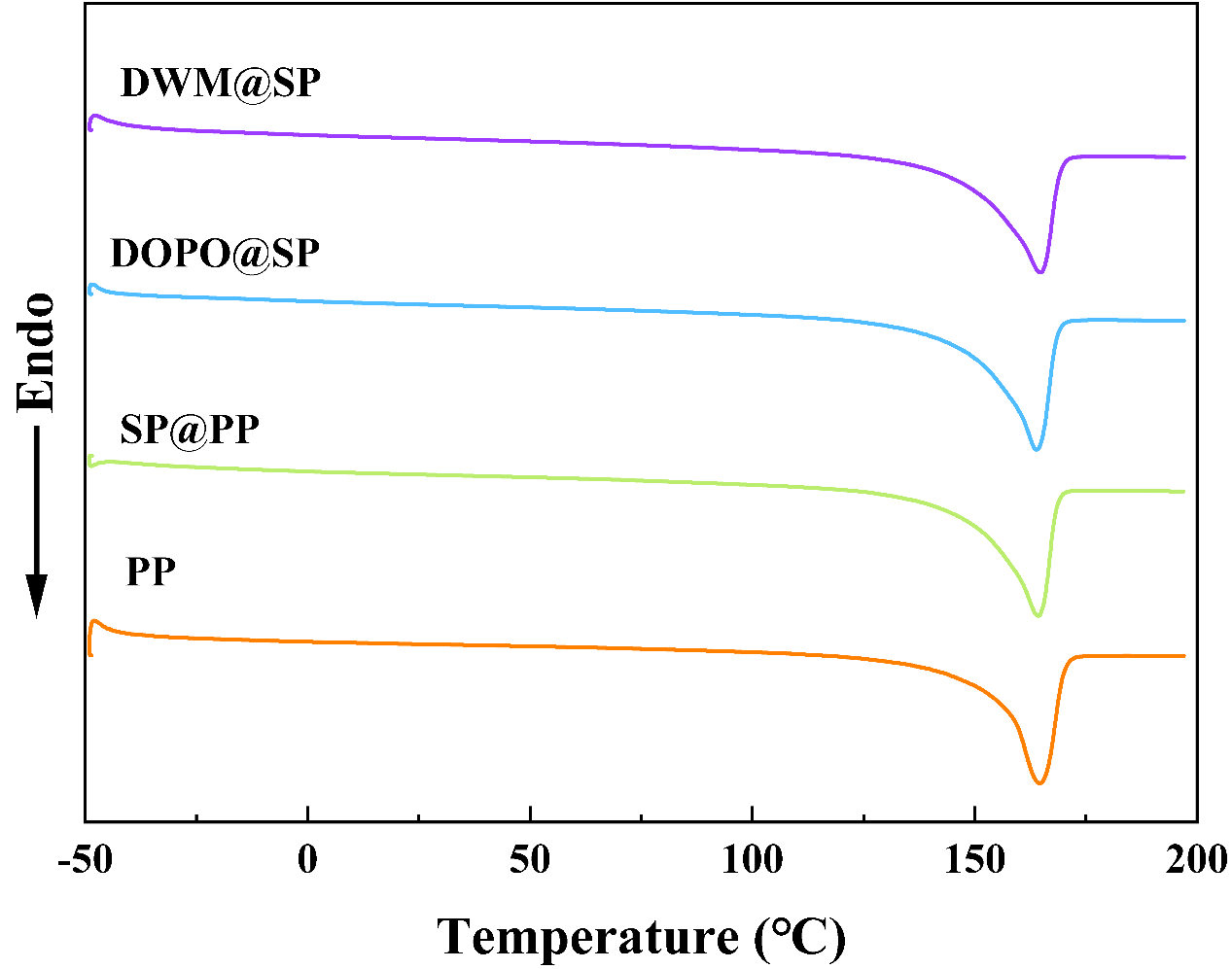
**

**Fig. S16.** DSC curves of different seperator.

**
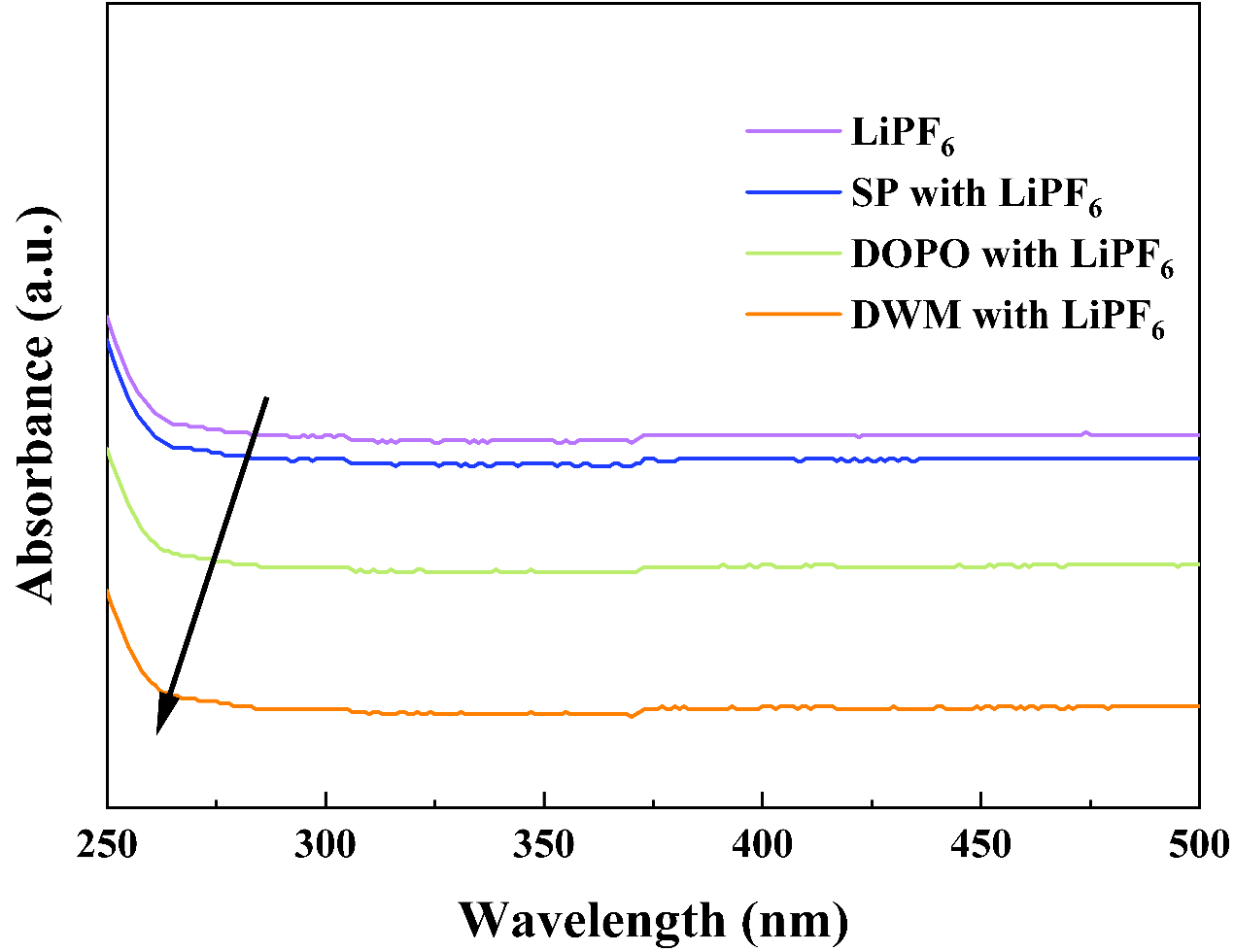
**

**Fig. S17.** Ultraviolet spectral absorption of different seperator.

**
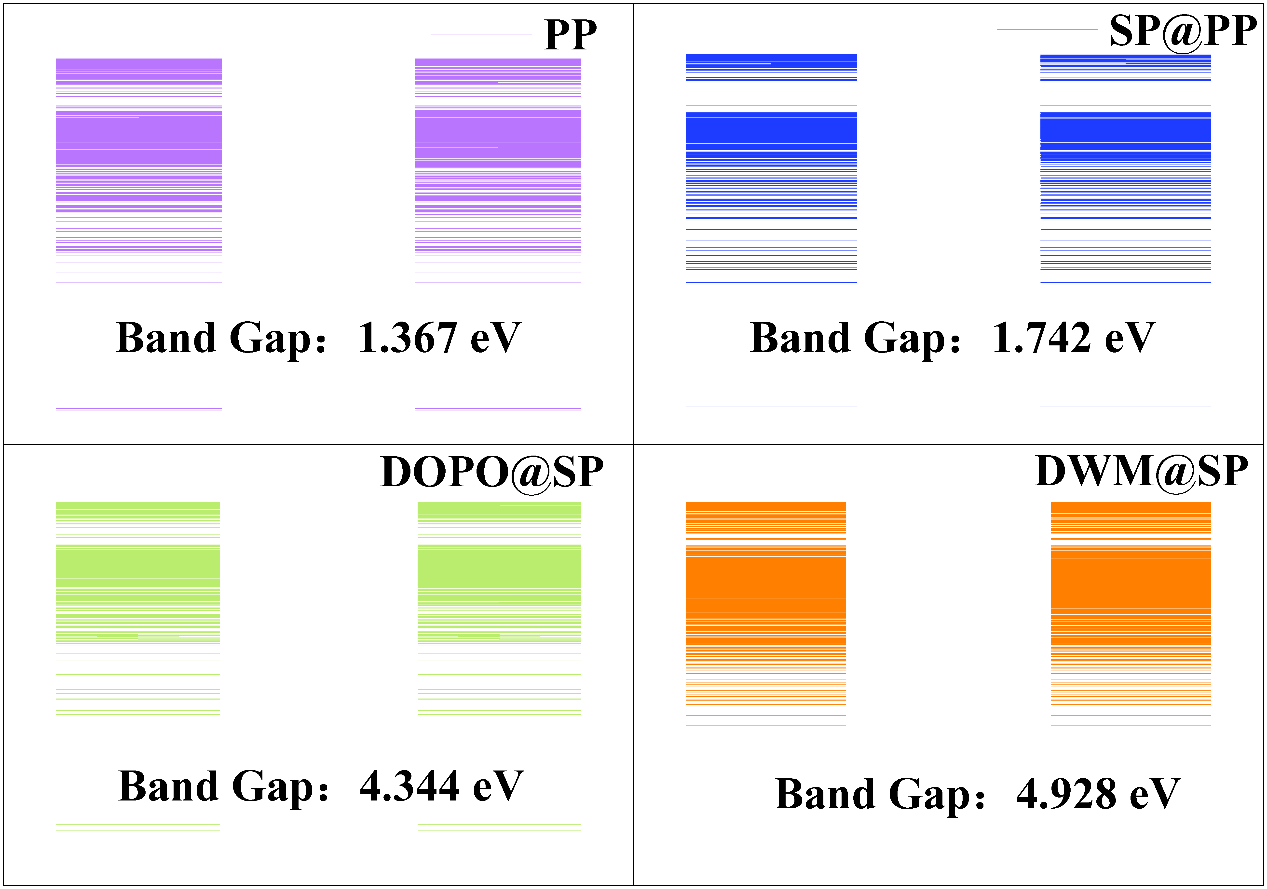
**

**Fig. S18.** Energy bands and band gaps of different seperator.

**
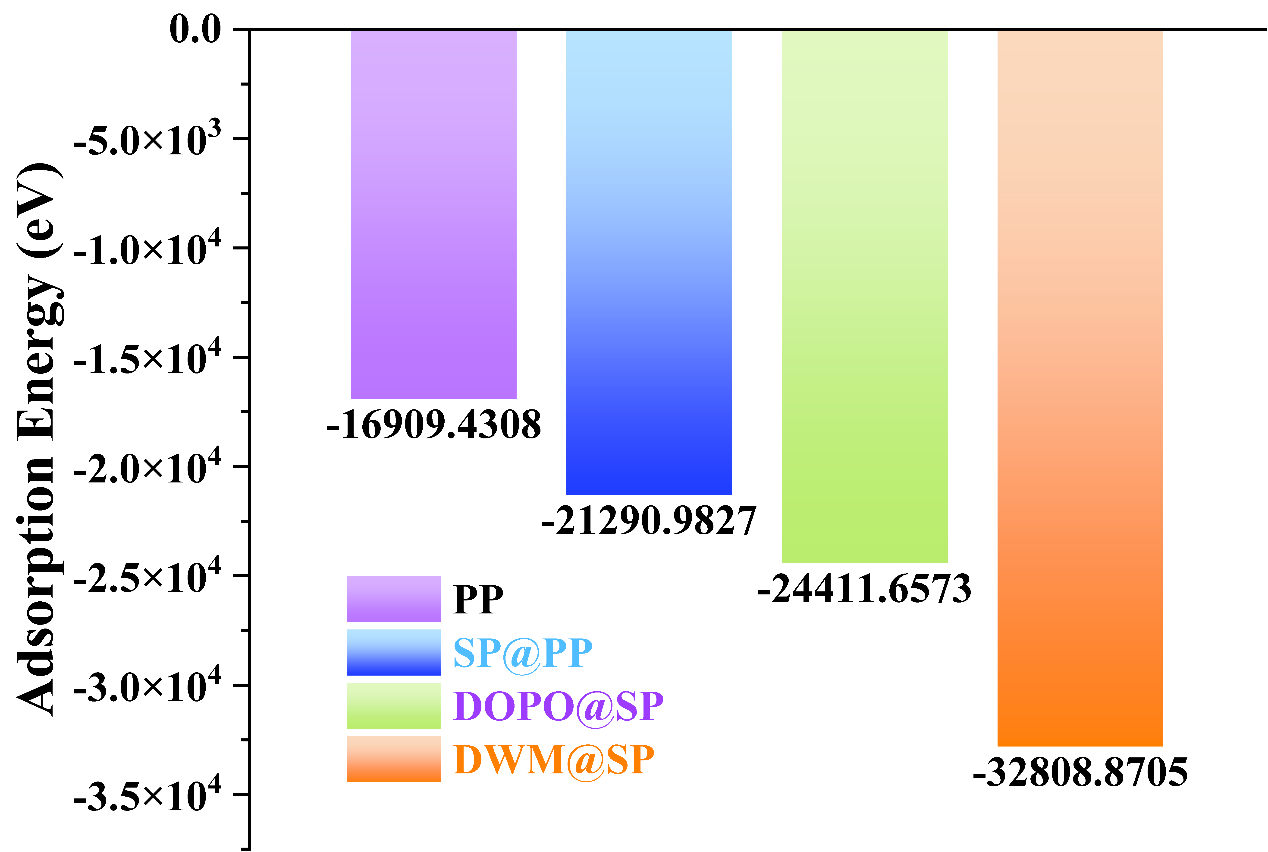
**

**Fig. S19.** Adsorption energyof different seperator.

**
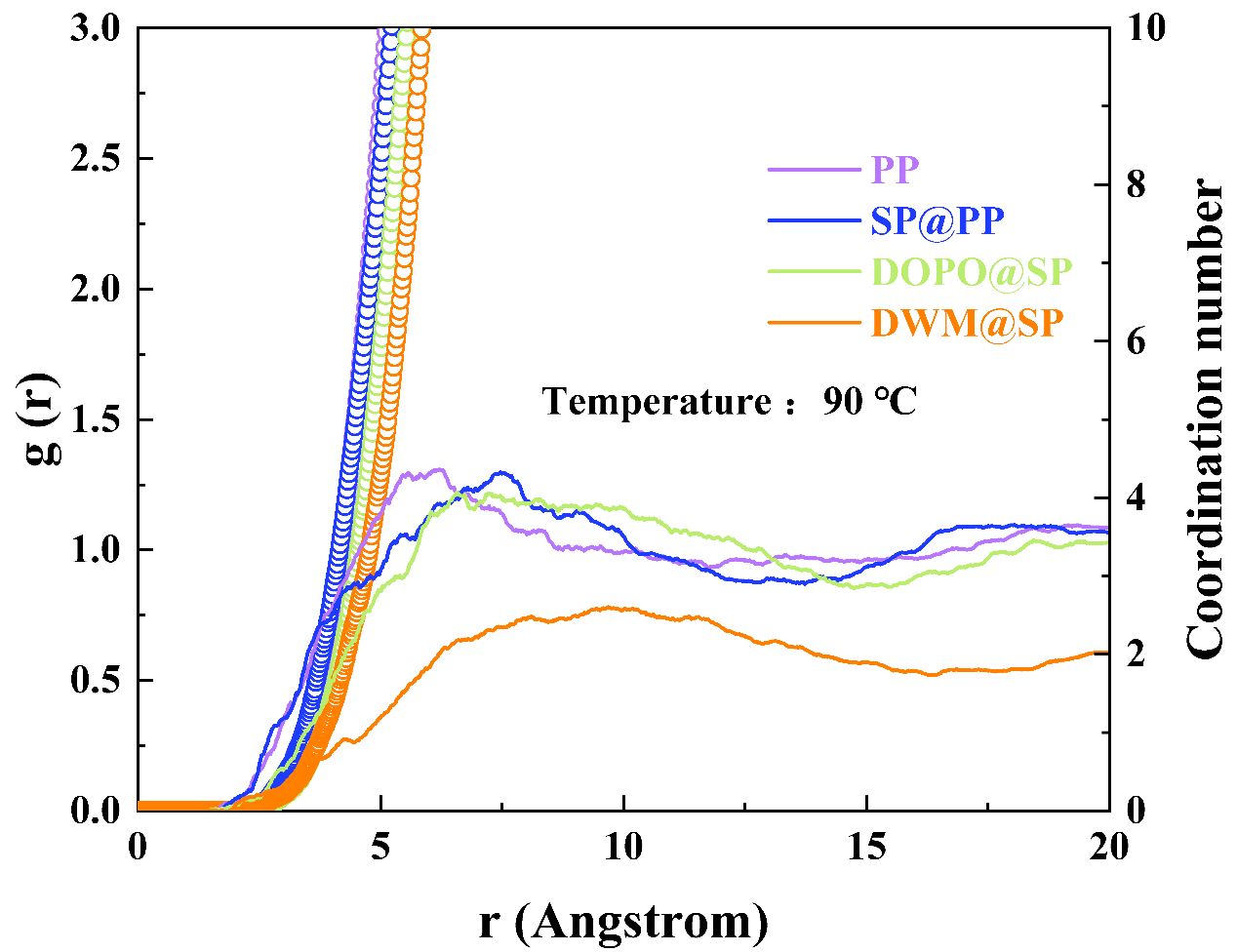
**

**Fig. S20.** Radial distribution diagram of different seperator at 90°C.**
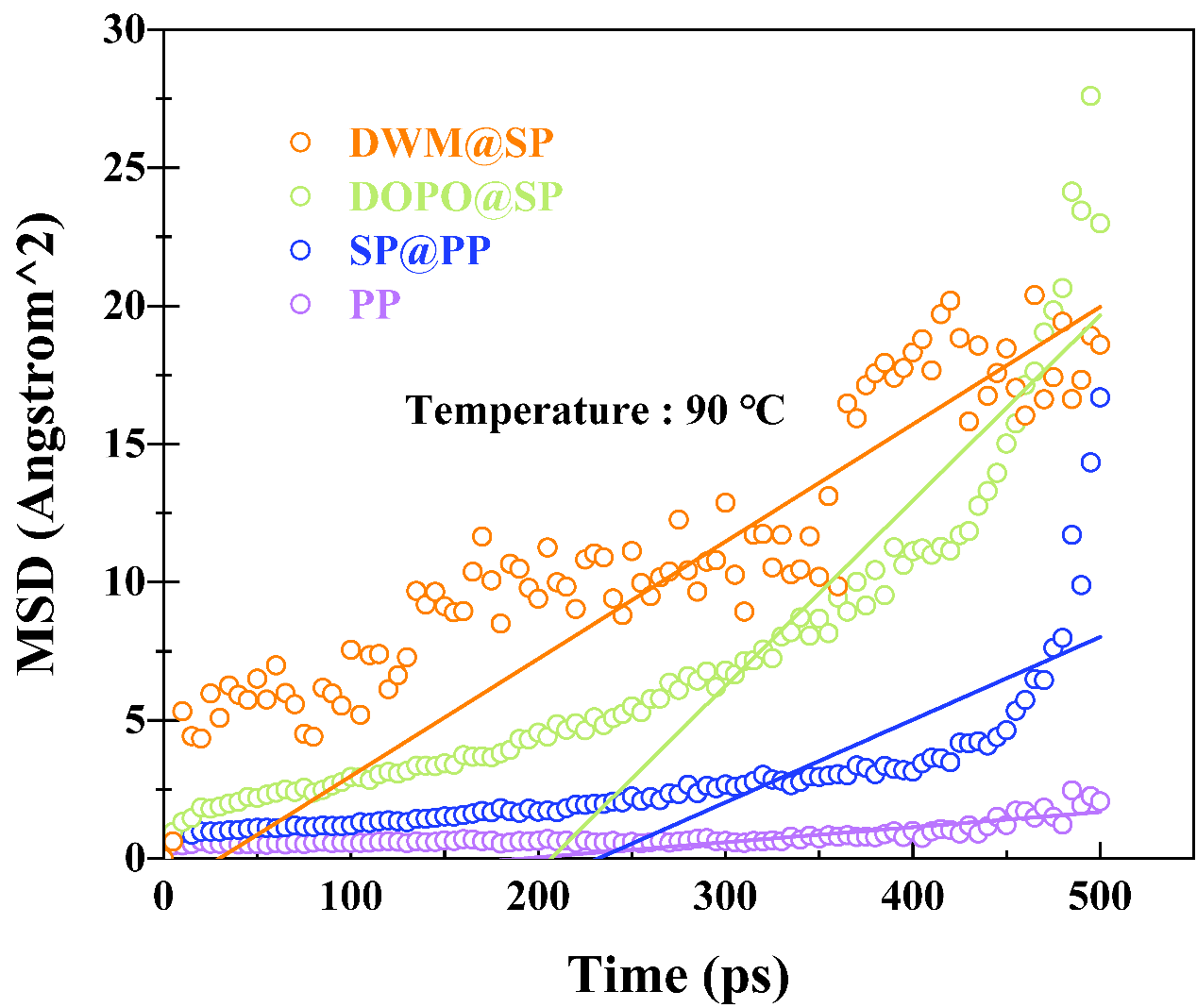
**

**Fig. S21.** Mean Square Displacement of different seperator at 90°C **.**

**
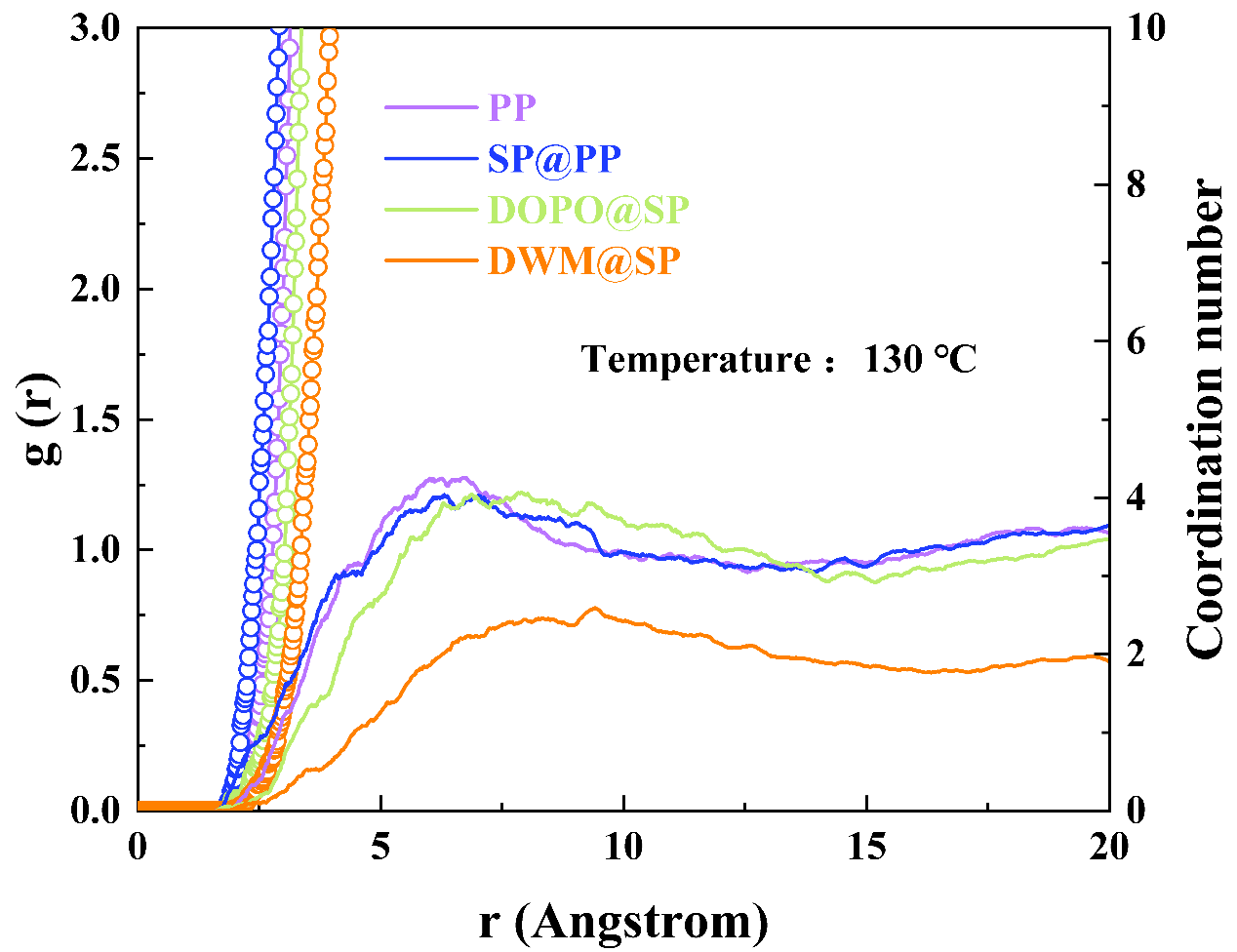
**

**Fig. S22.** Radial distribution diagram of different seperator at 130°C. **
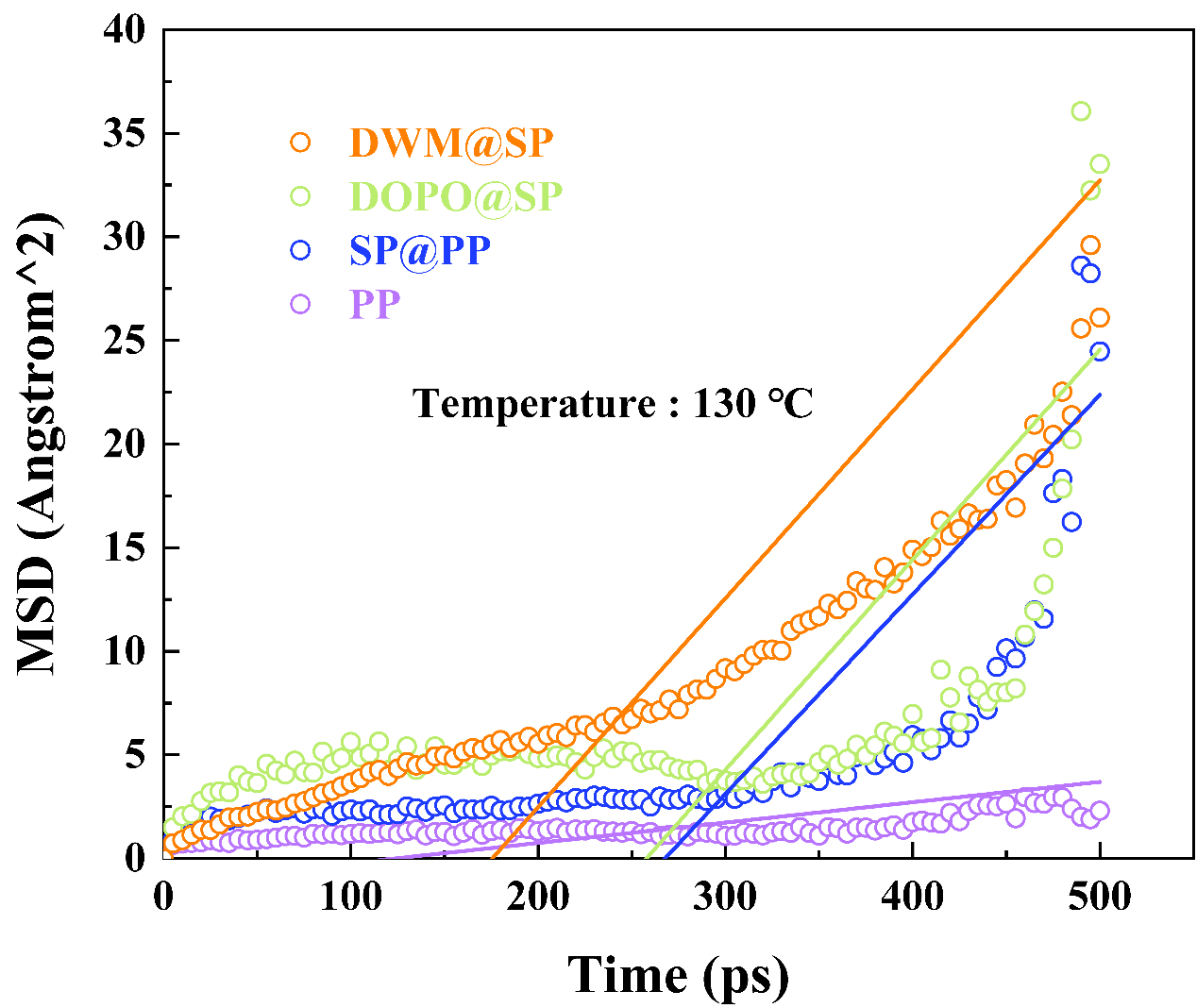
**

**Fig. S23.** Mean Square Displacement of different seperator at 130°C.

**
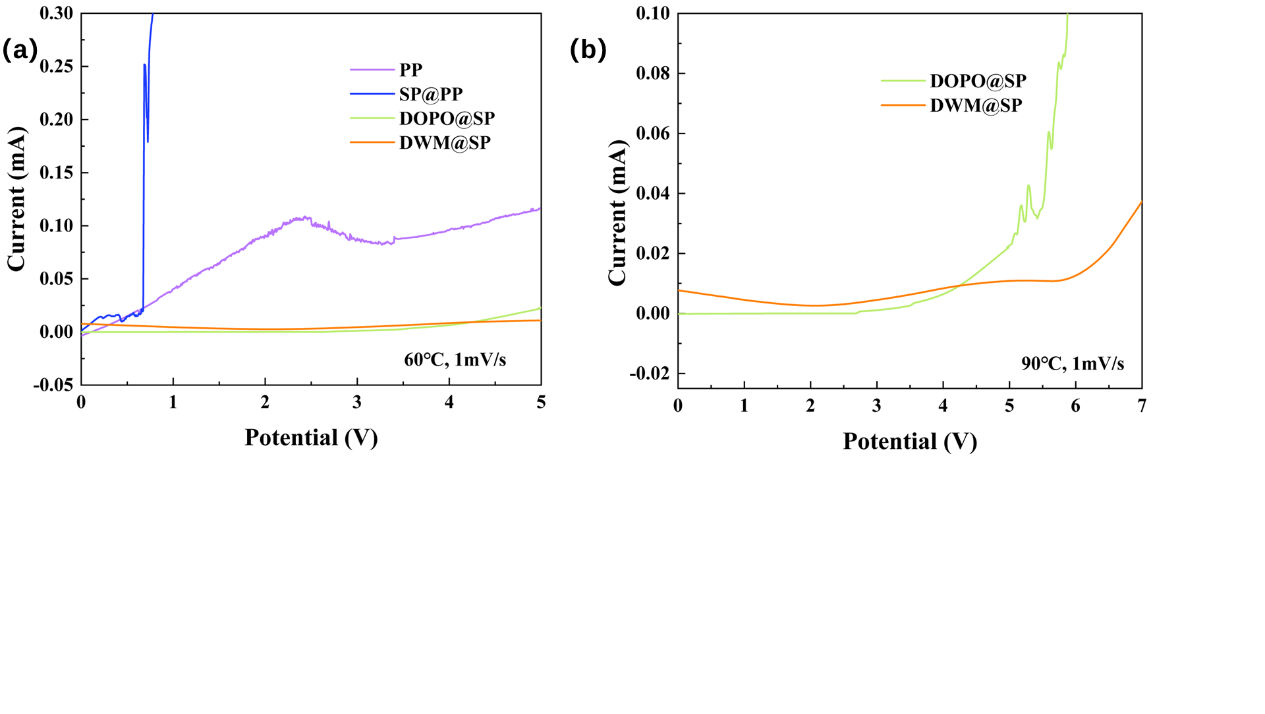
**

**Fig. S24.** Lsv linear scan curves at different temperatures.a at 60℃and b at 90℃.

**
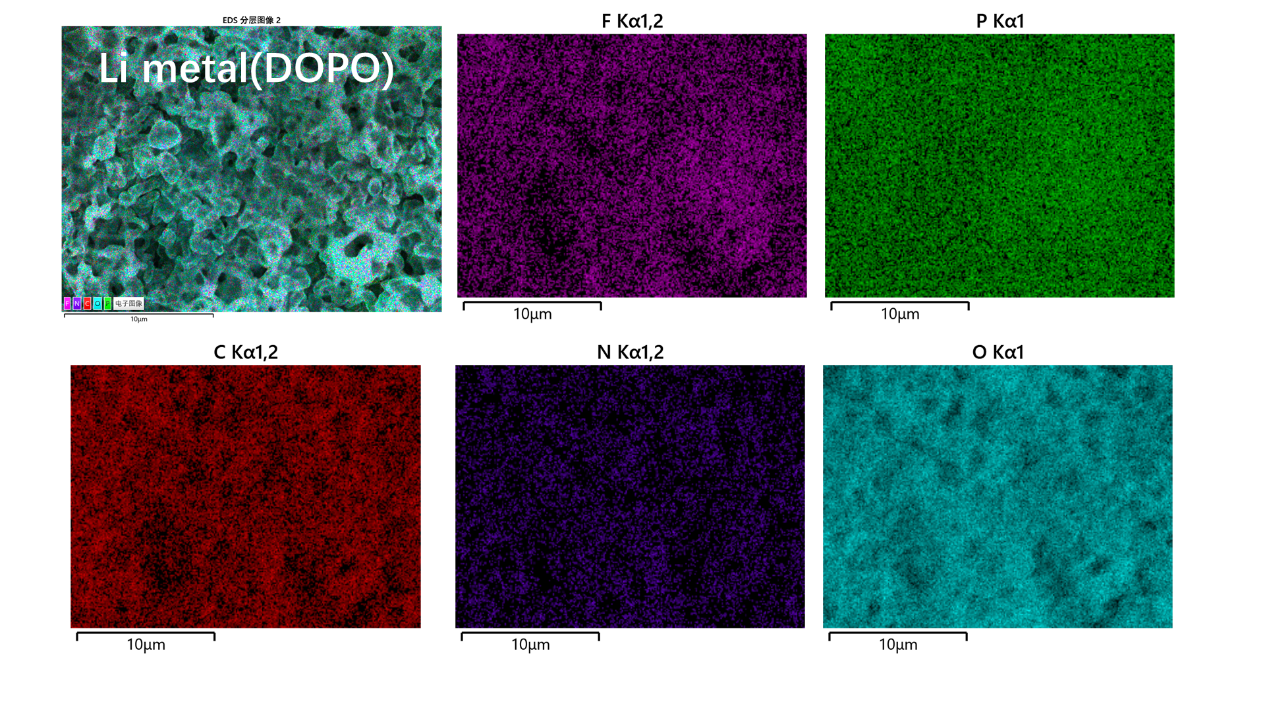
**

**Fig. S25.** SEM and EDS spectra of lithium anodes after high-temperature testing of DOPO@SP seperator.

**
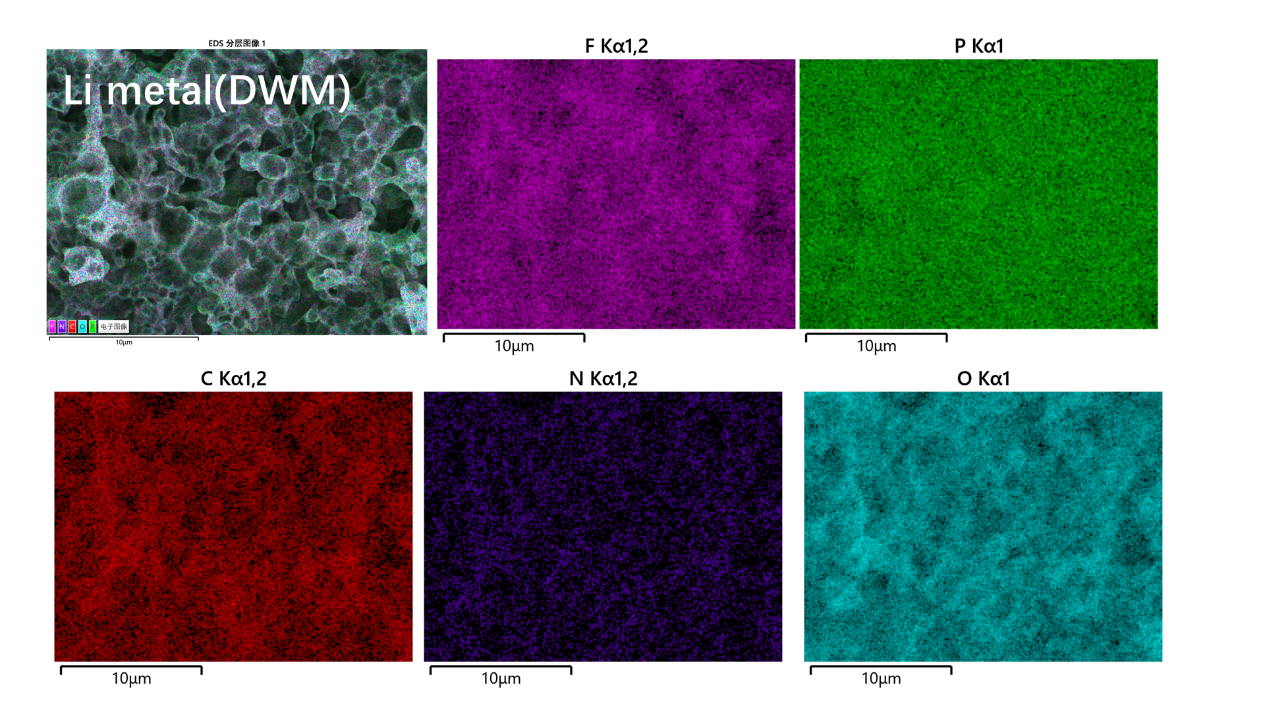
**

**Fig. S26.** SEM and EDS spectra of lithium anodes after high-temperature testing of DWM@SP seperator.


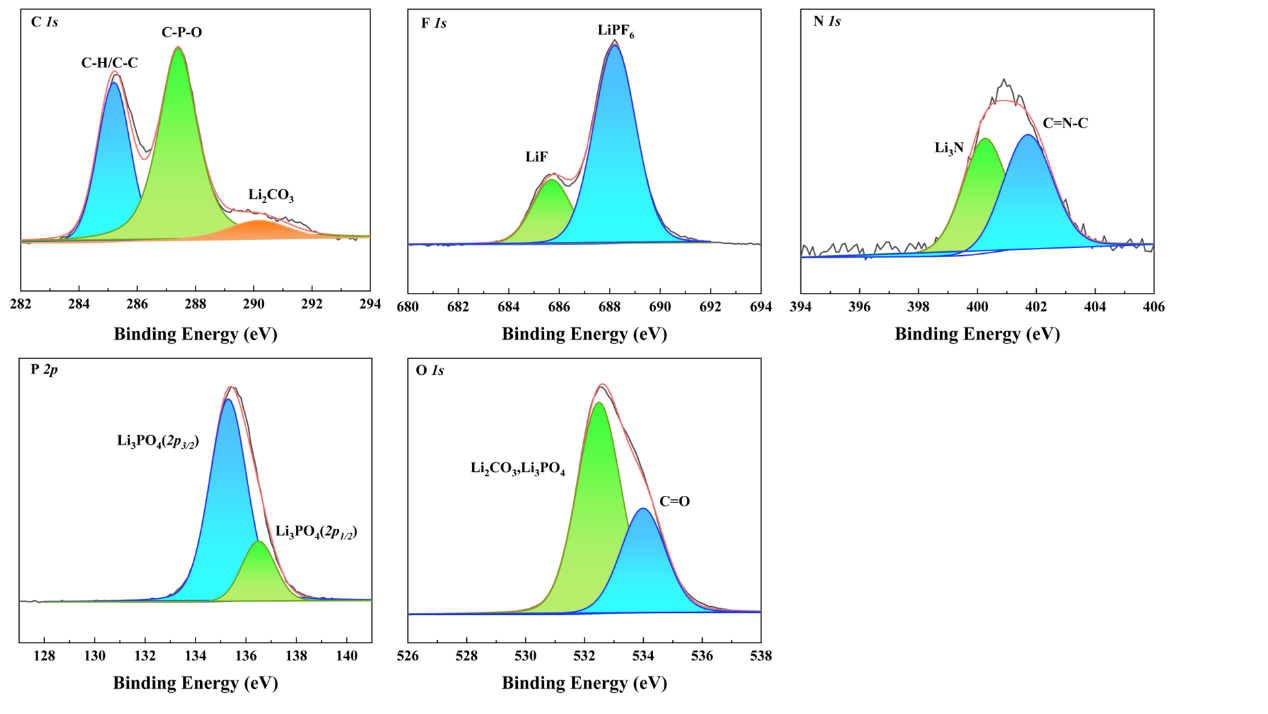


**Fig. S27.** XPS elemental spectrum of the DWM@SP seperator after high-temperature testing of 1000h.


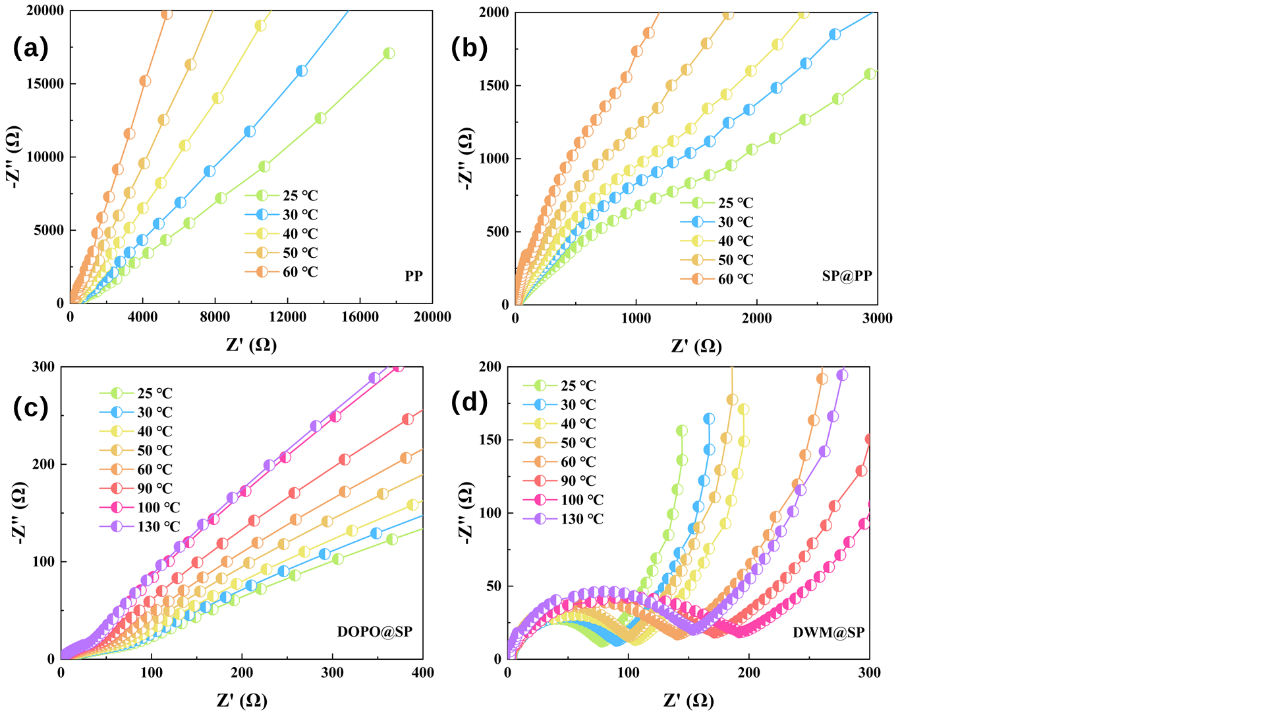


**Fig. S28.** Nyquist plots of the SS/seperator/SS symmetrical cell at various

temperatures with different LiPF_6_ contents: a PP, b SP@PP, c DOPO@SP, d DWM@SP.

**
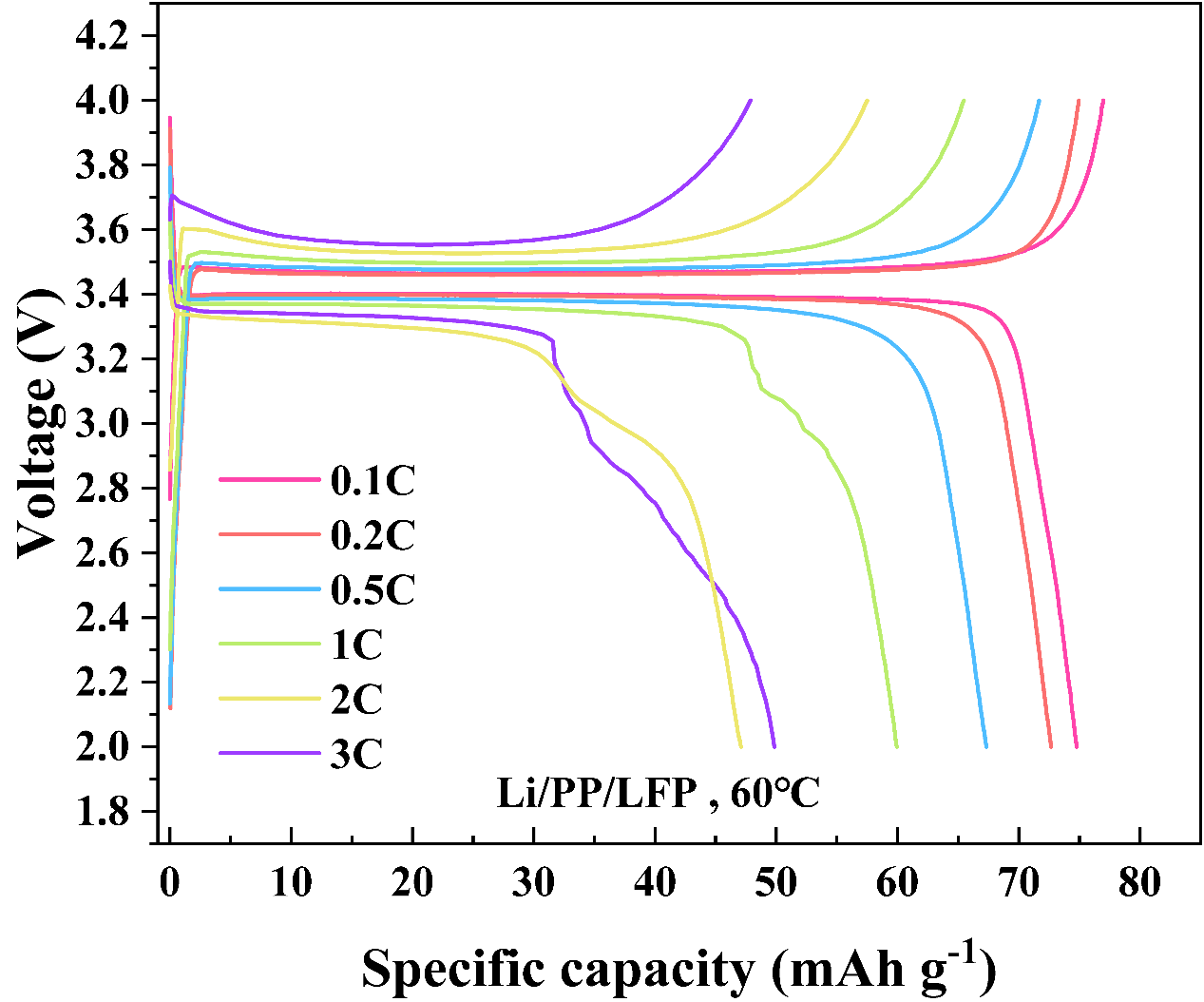
**

**Fig. S29.** Charge-discharge curve of PP seperator at 60°C.

**
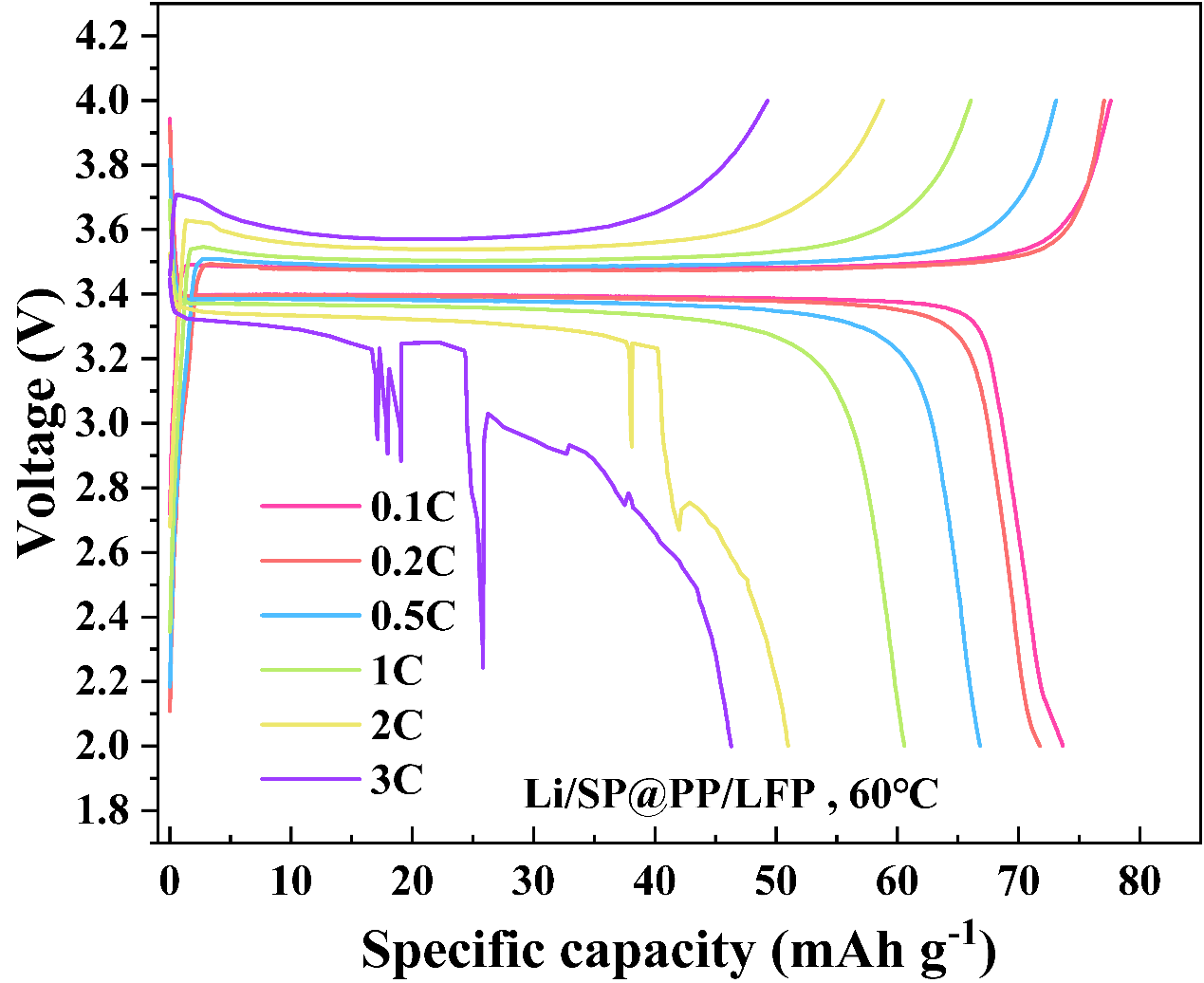
**

**Fig. S30.** Charge-discharge curve of SP@PP seperator at 60°C.

**
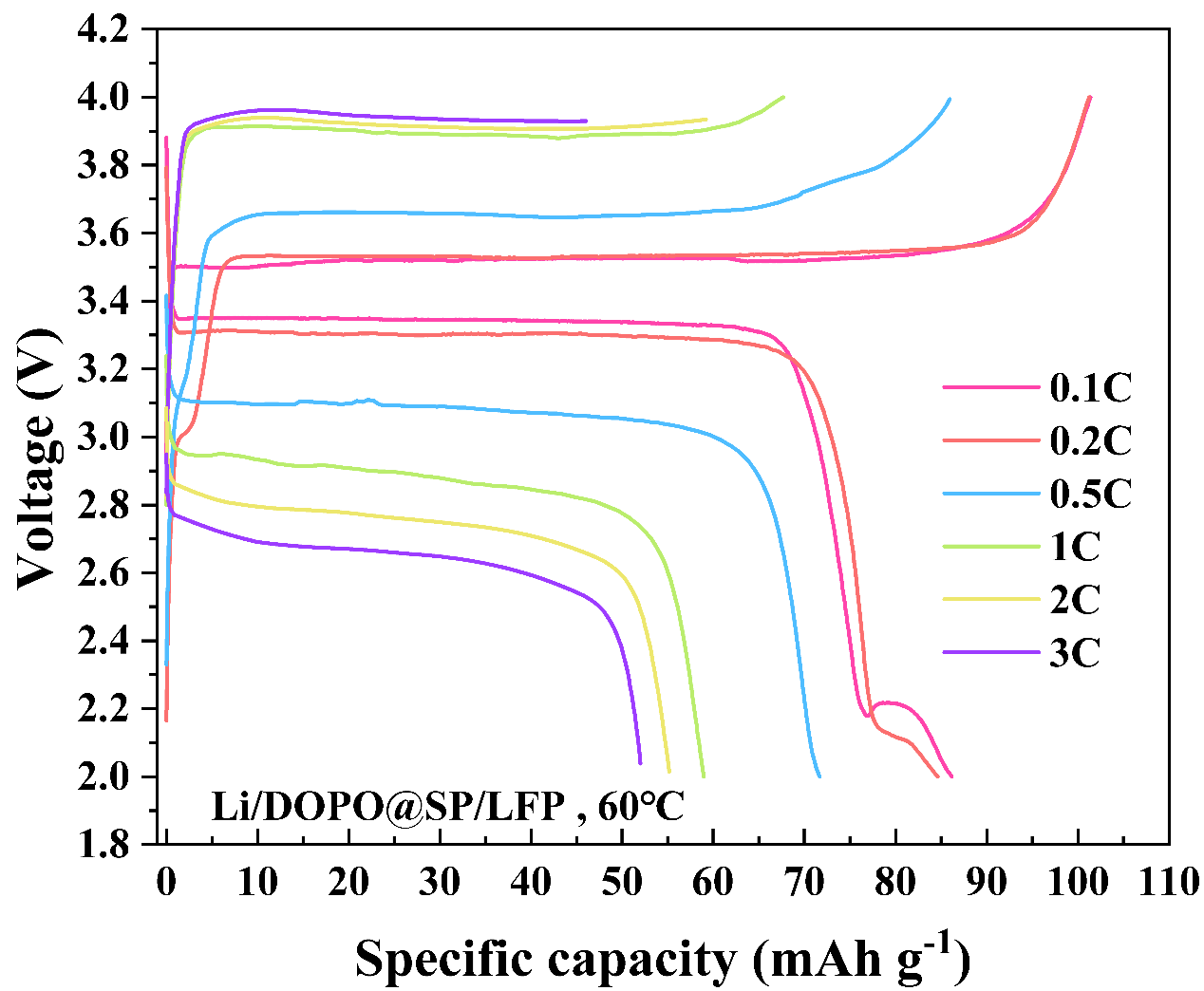
**

**Fig. S31.** Charge-discharge curve of DOPO@SP seperator at 60°C.

**
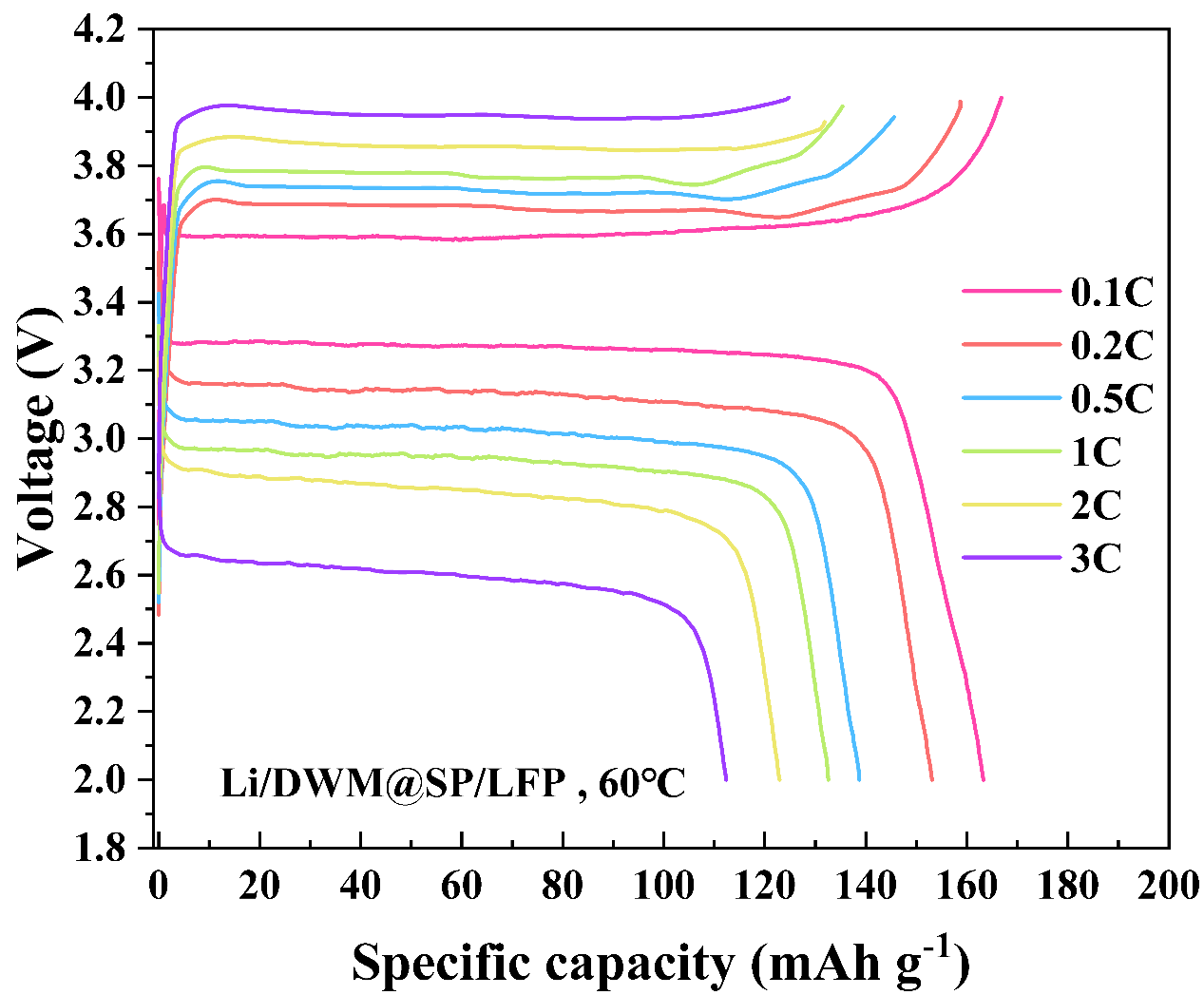
**

**Fig. S32.** Charge-discharge curve of DWM@SP seperator at 60°C.

**
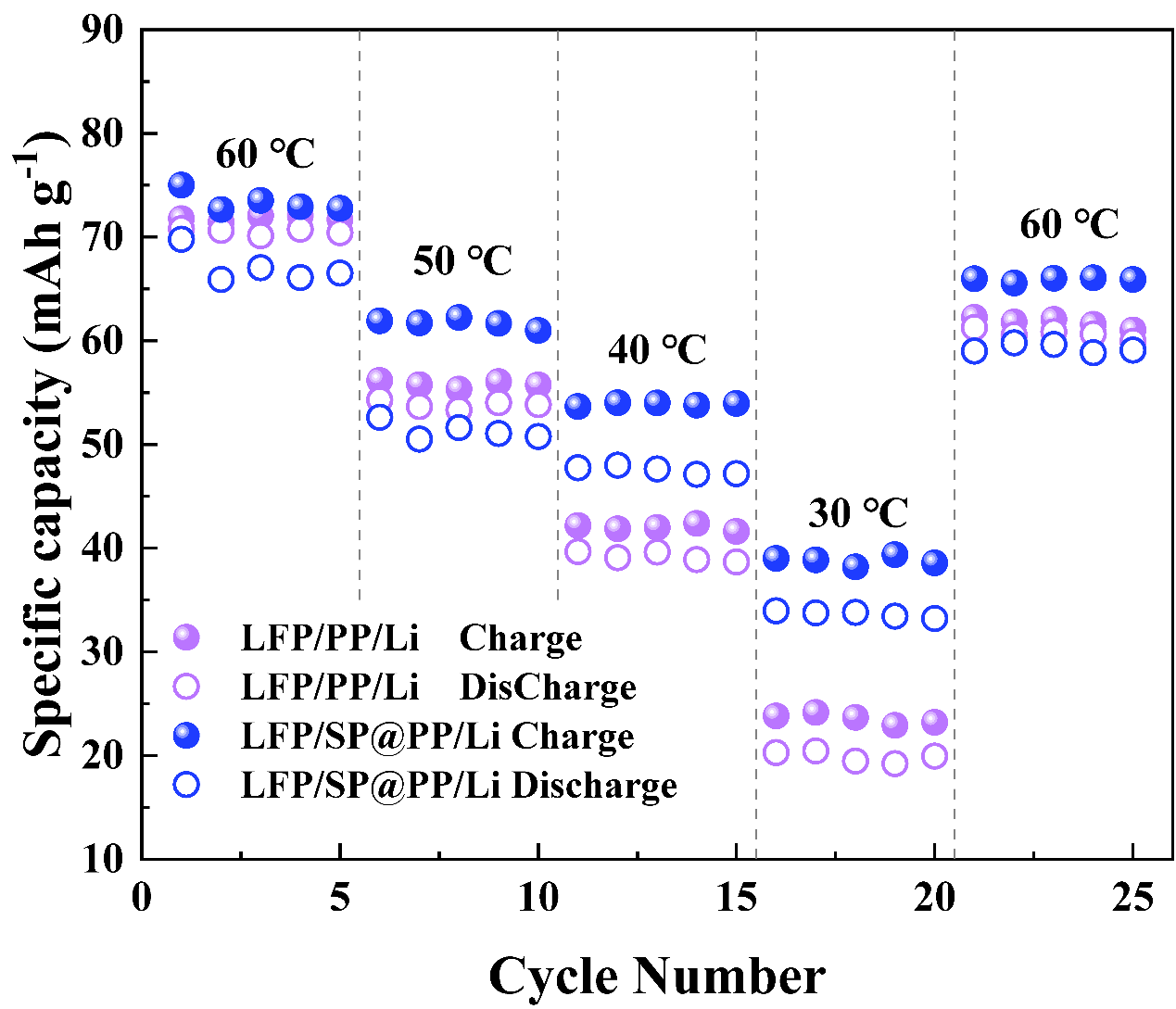
**

**Fig. S33.** PP seperator and SP@PP seperator rate under heating and cooling conditions.

**
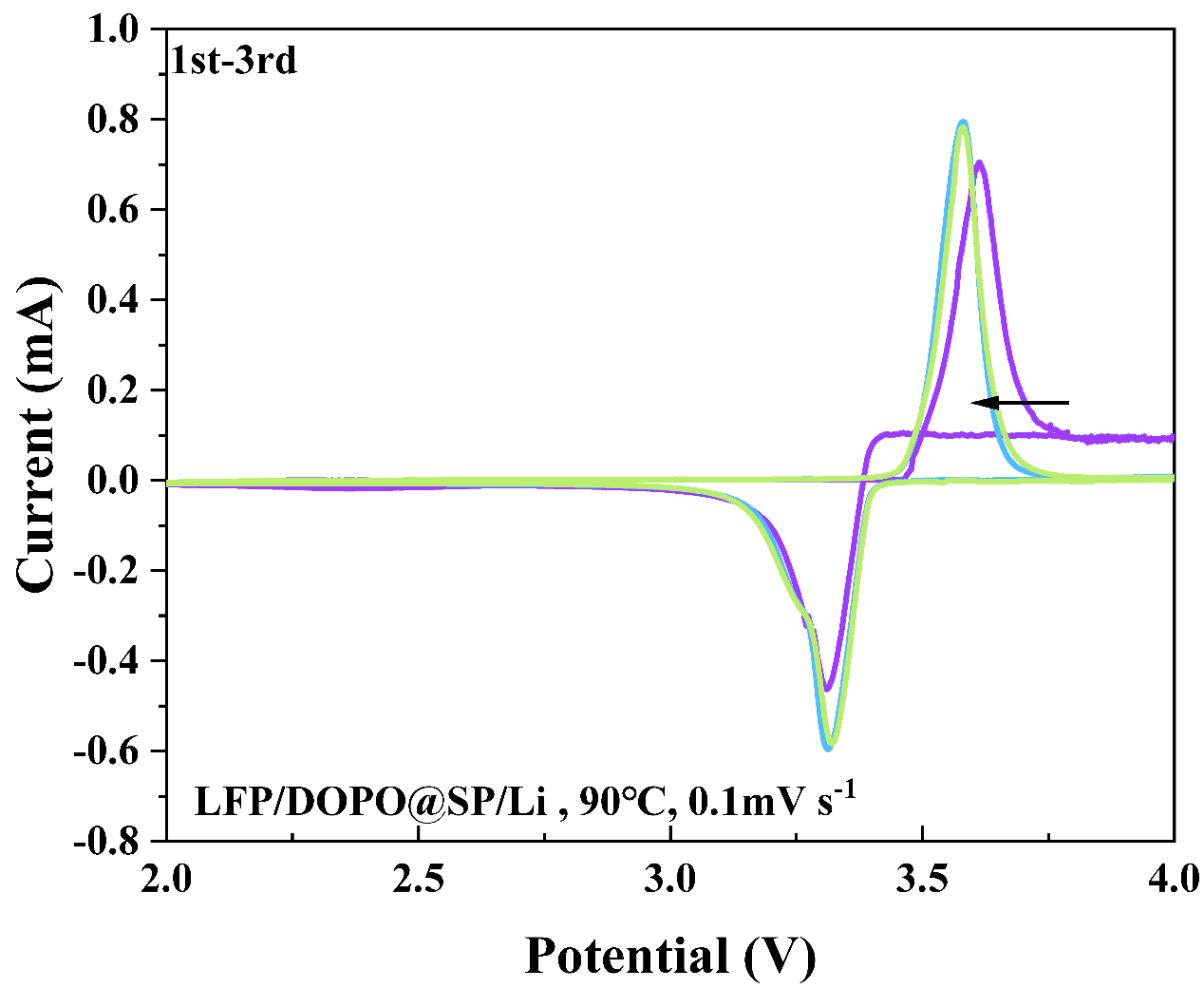
**

**Fig. S34.** CV cycle curve of DOPO@SP seperator at 90°C. **
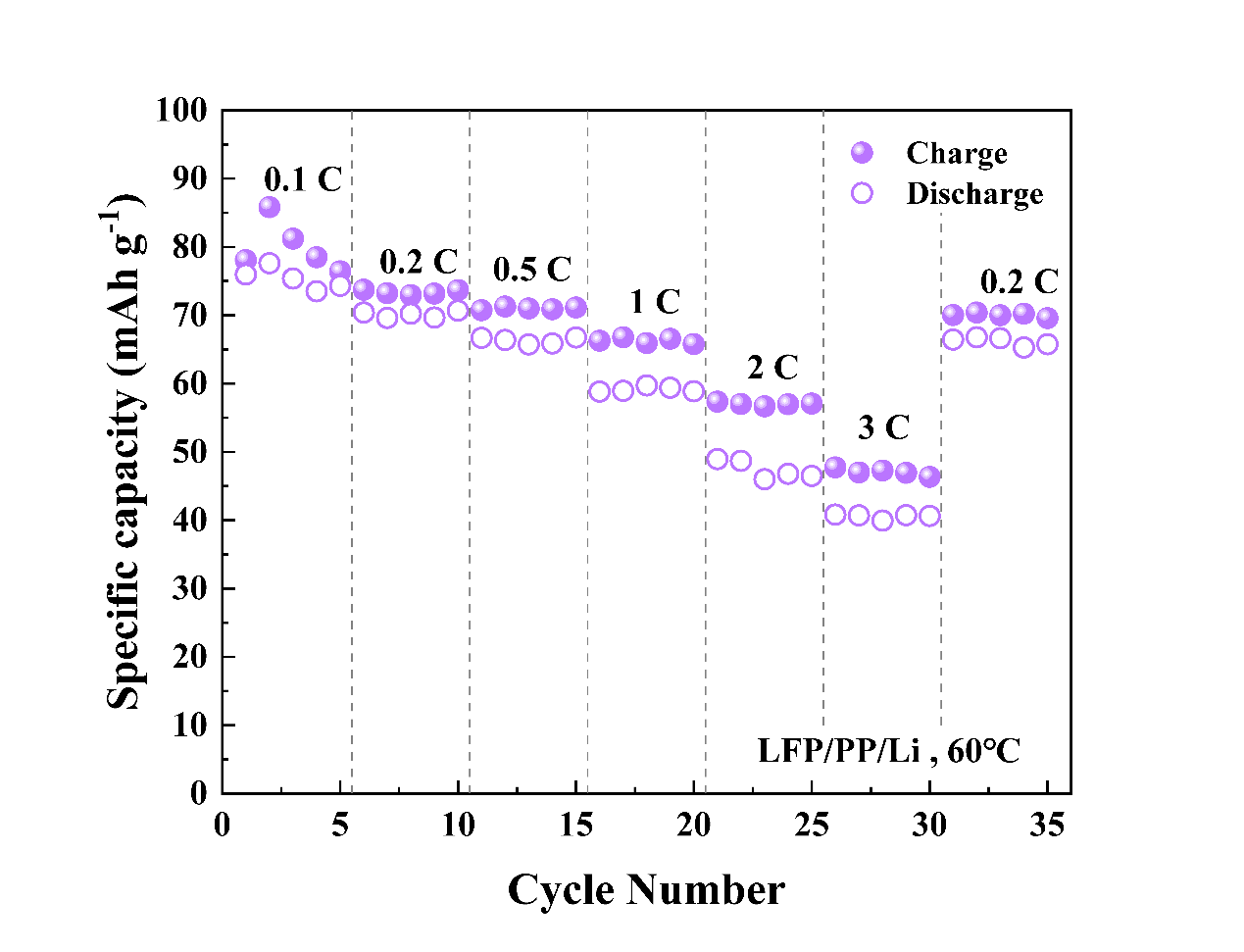
**

**Fig. S35.** Rating performance of PP seperator at 60°C.

**
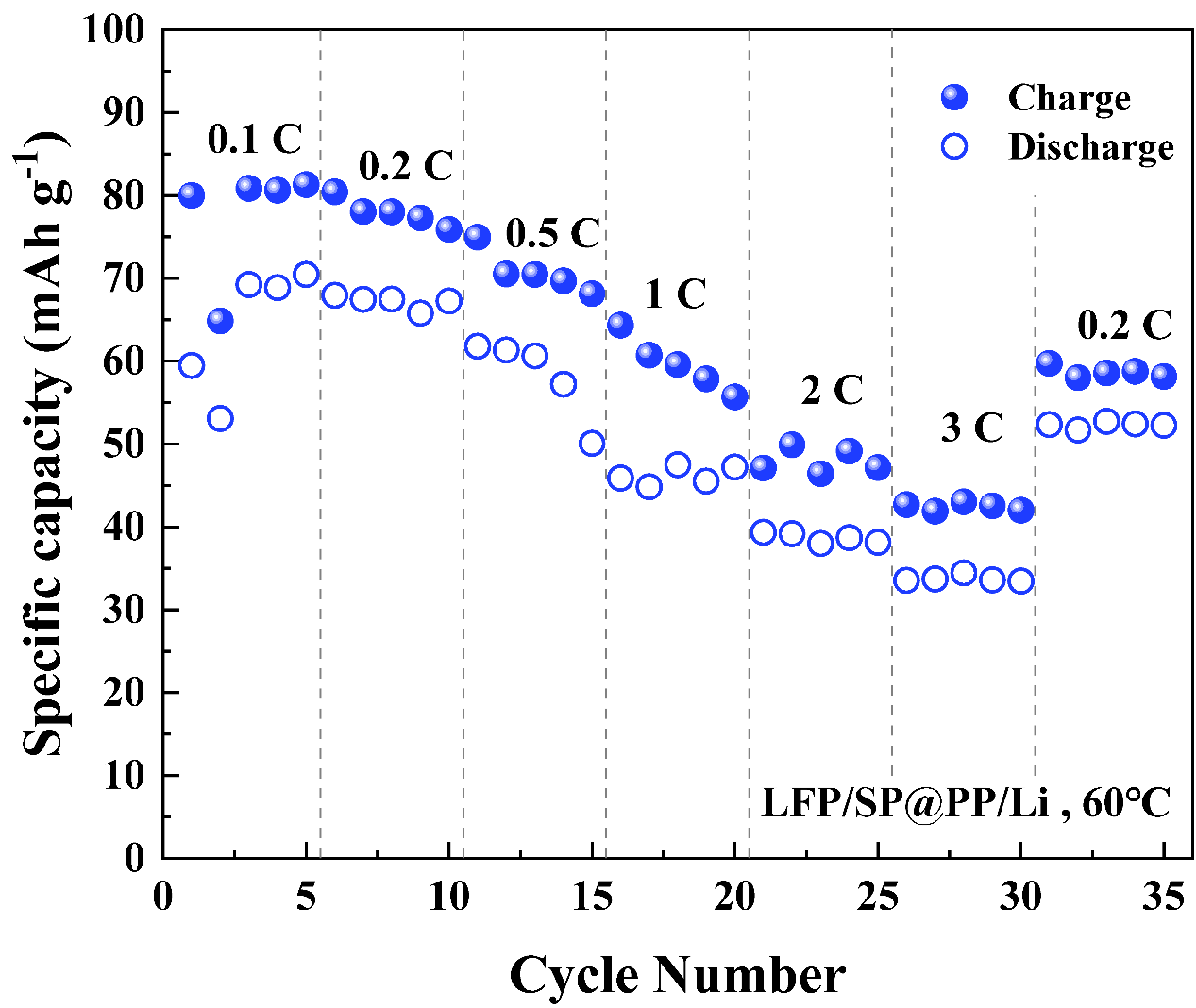
**

**Fig. S36.** Rating performance of SP@PP seperator at 60°C.

**
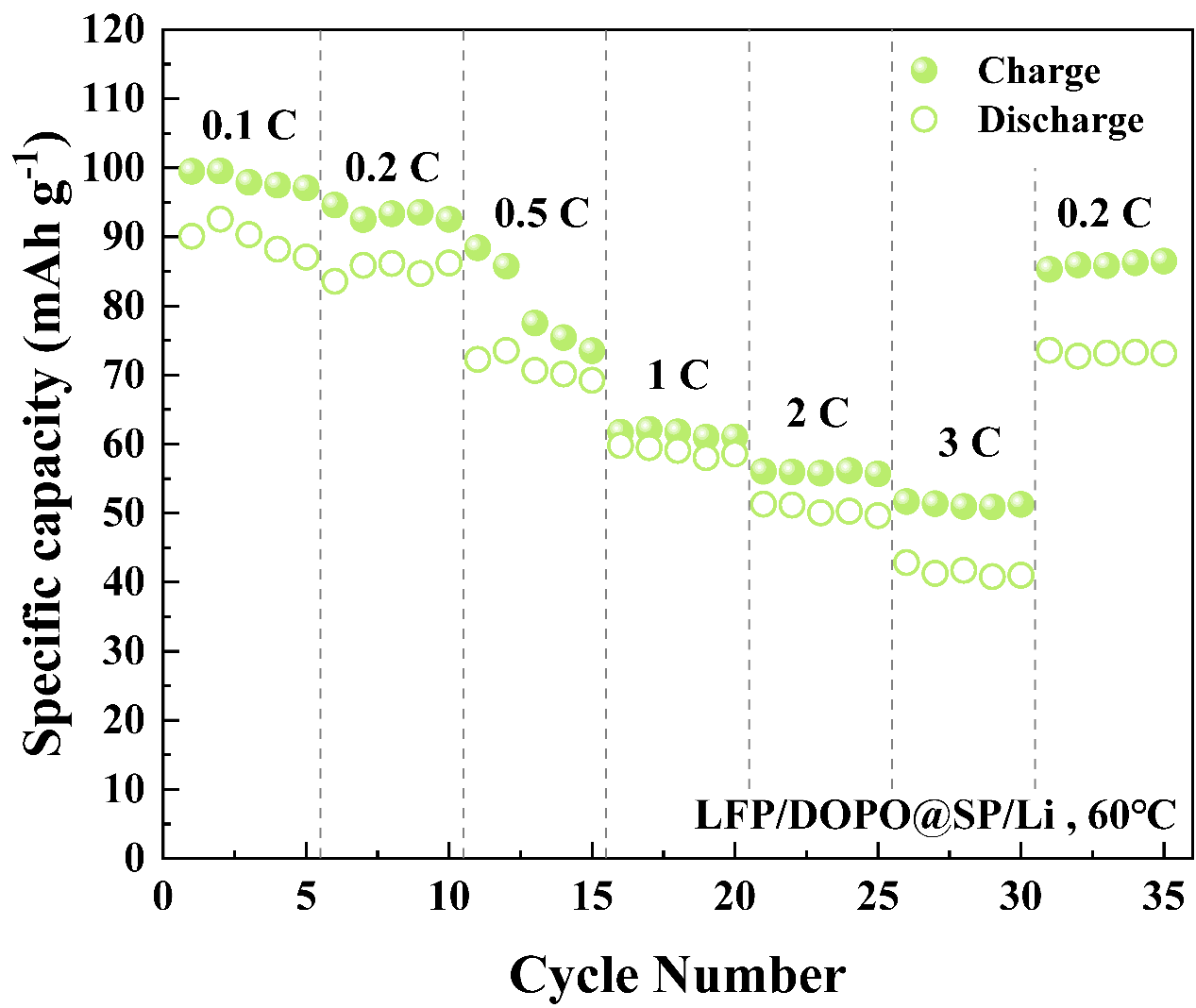
**

**Fig. S37.** Rating performance of DOPO@SP seperator at 60°C.

**
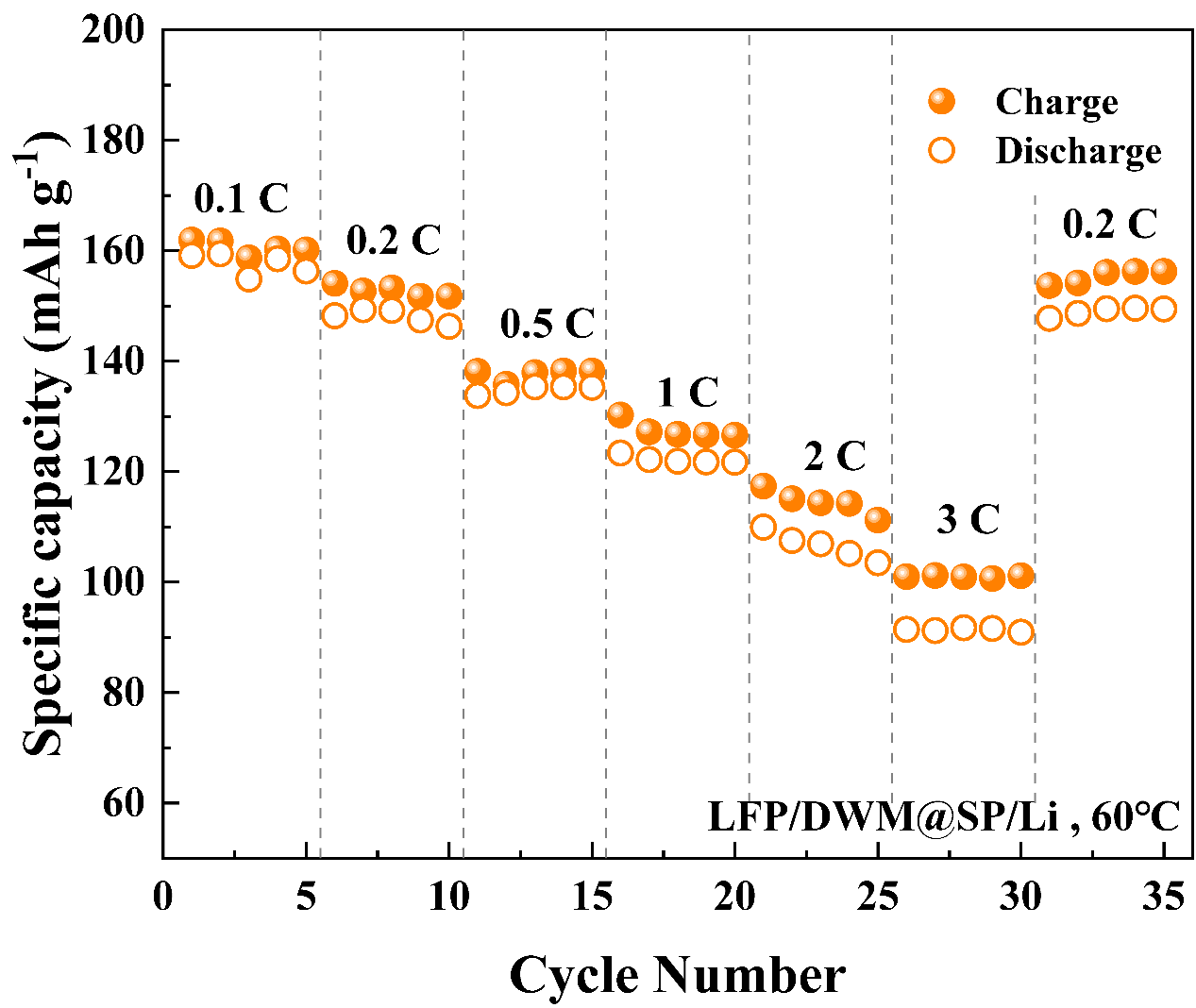
**

**Fig. S38.** Rating performance of DWM@SP seperator at 60°C.

**
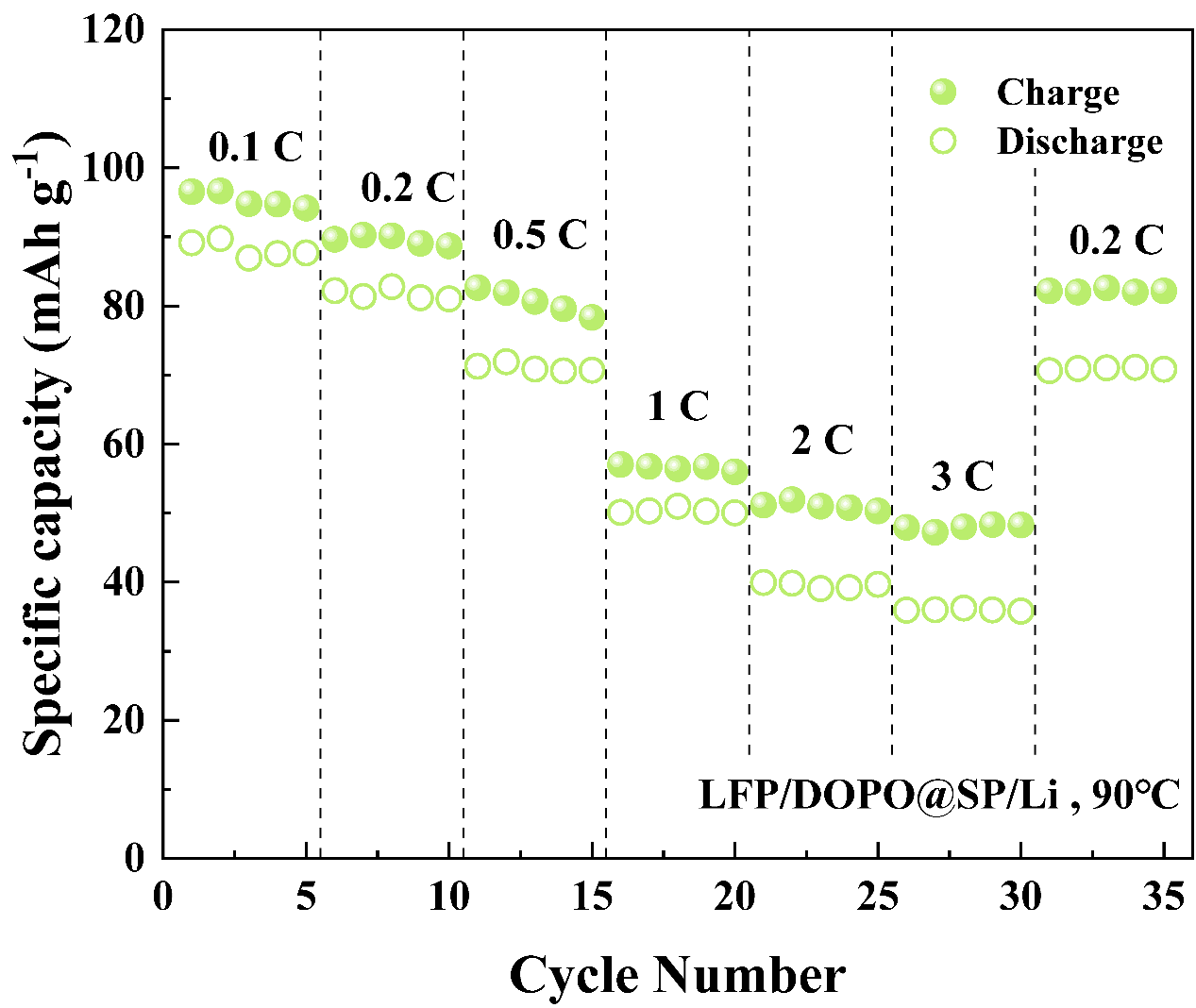
**

**Fig. S39.** Rating performance of DOPO@SP seperator at 90°C.

**
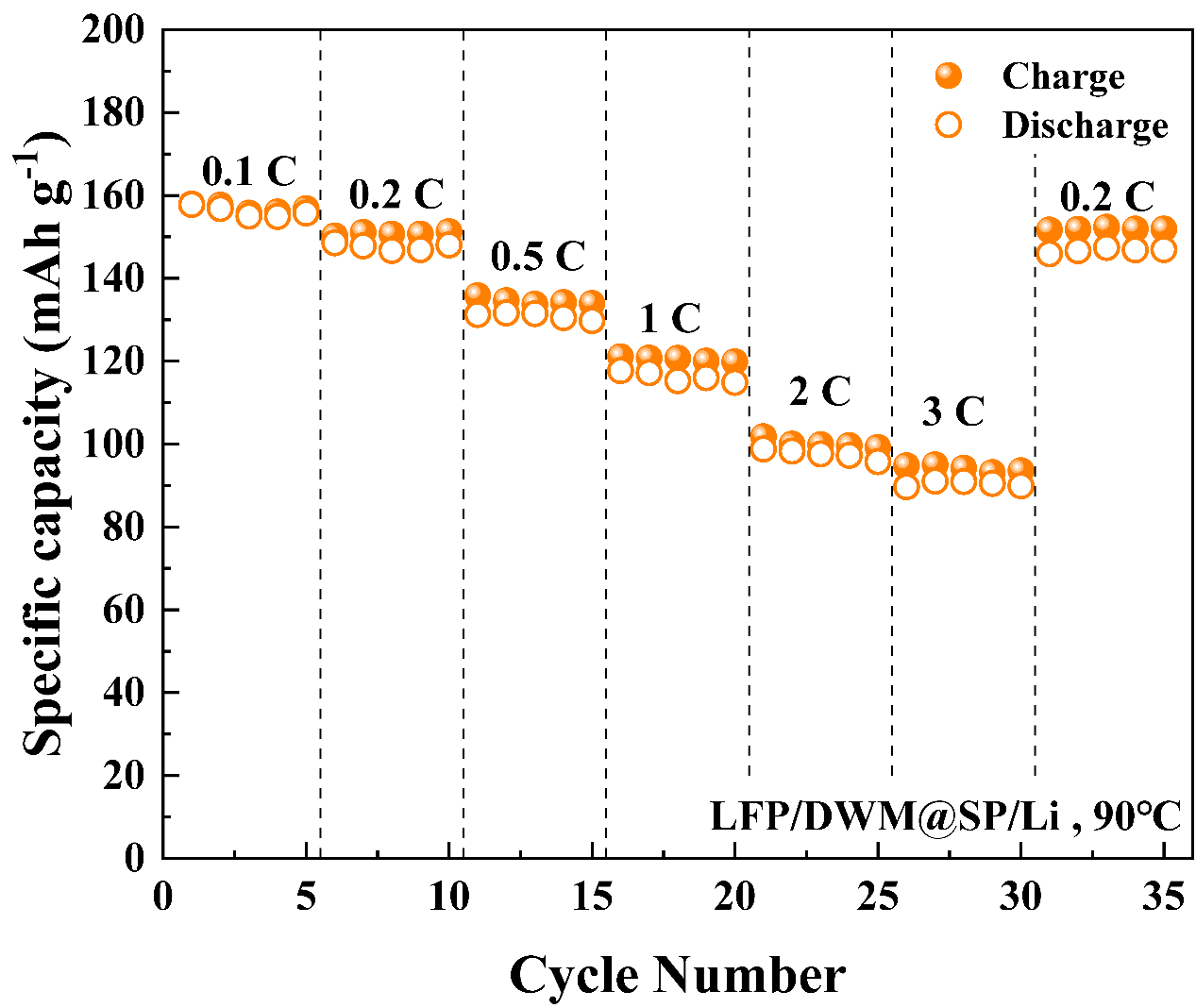
**

**Fig. S40.** Rating performance of DWM@SP seperator at 90°C.

**
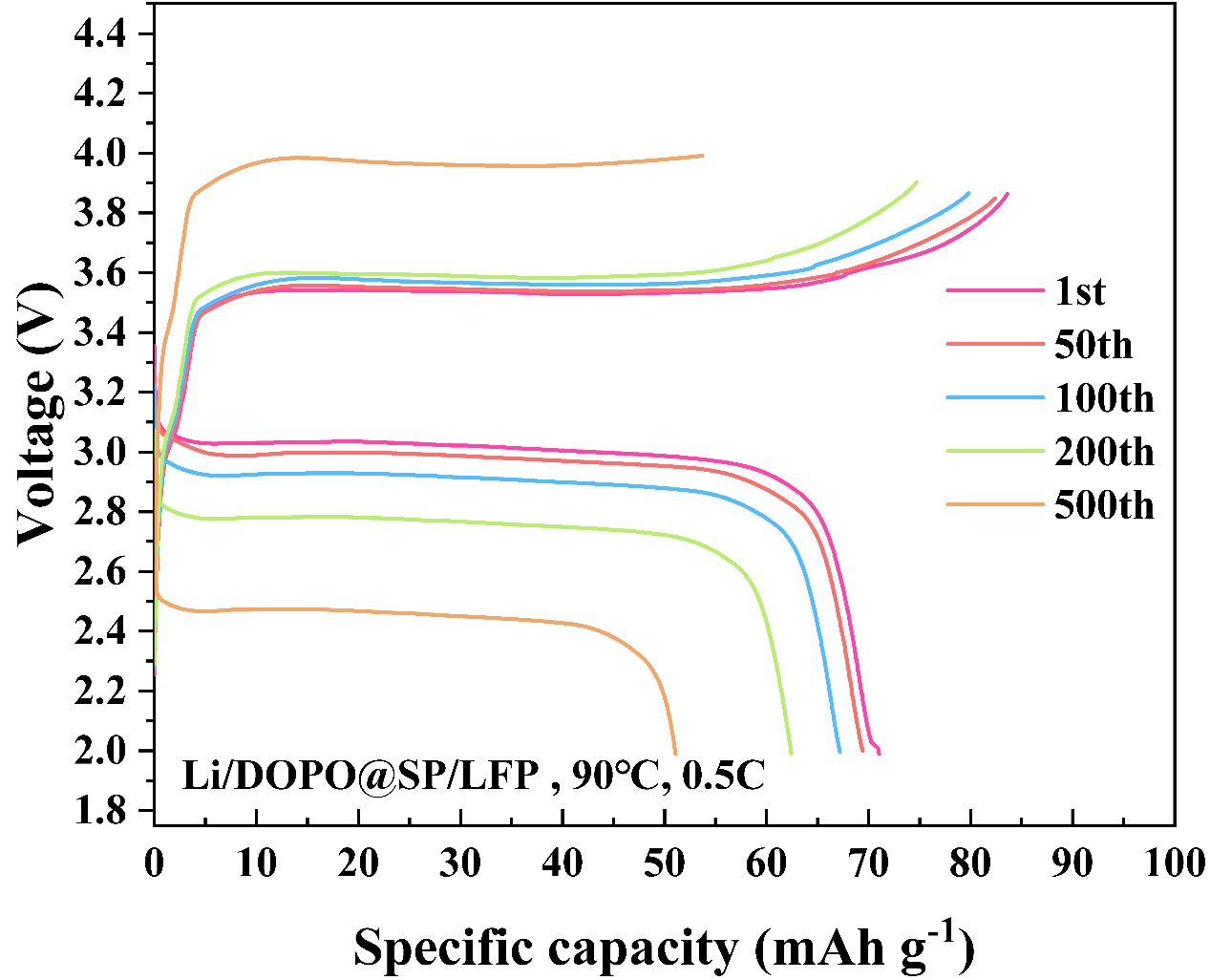
**

**Fig. S41.** The specific capacity-voltage curves of LFP/DOPO@SP/Li cell at 0.5C and 90 °C. **
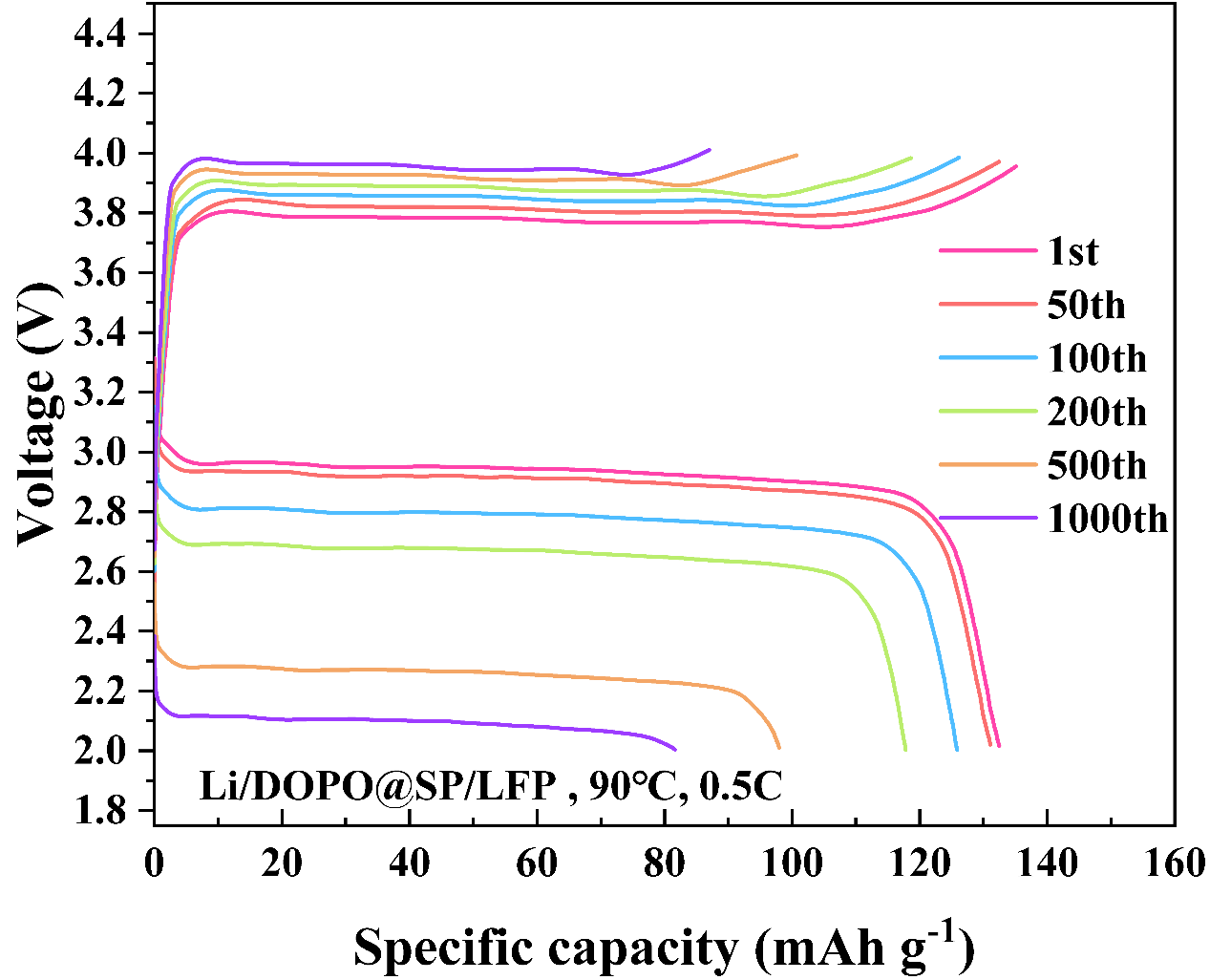
**

**Fig. S42.** The specific capacity-voltage curves of LFP/DWMO@SP/Li cell at 0.5C and 90 °C.


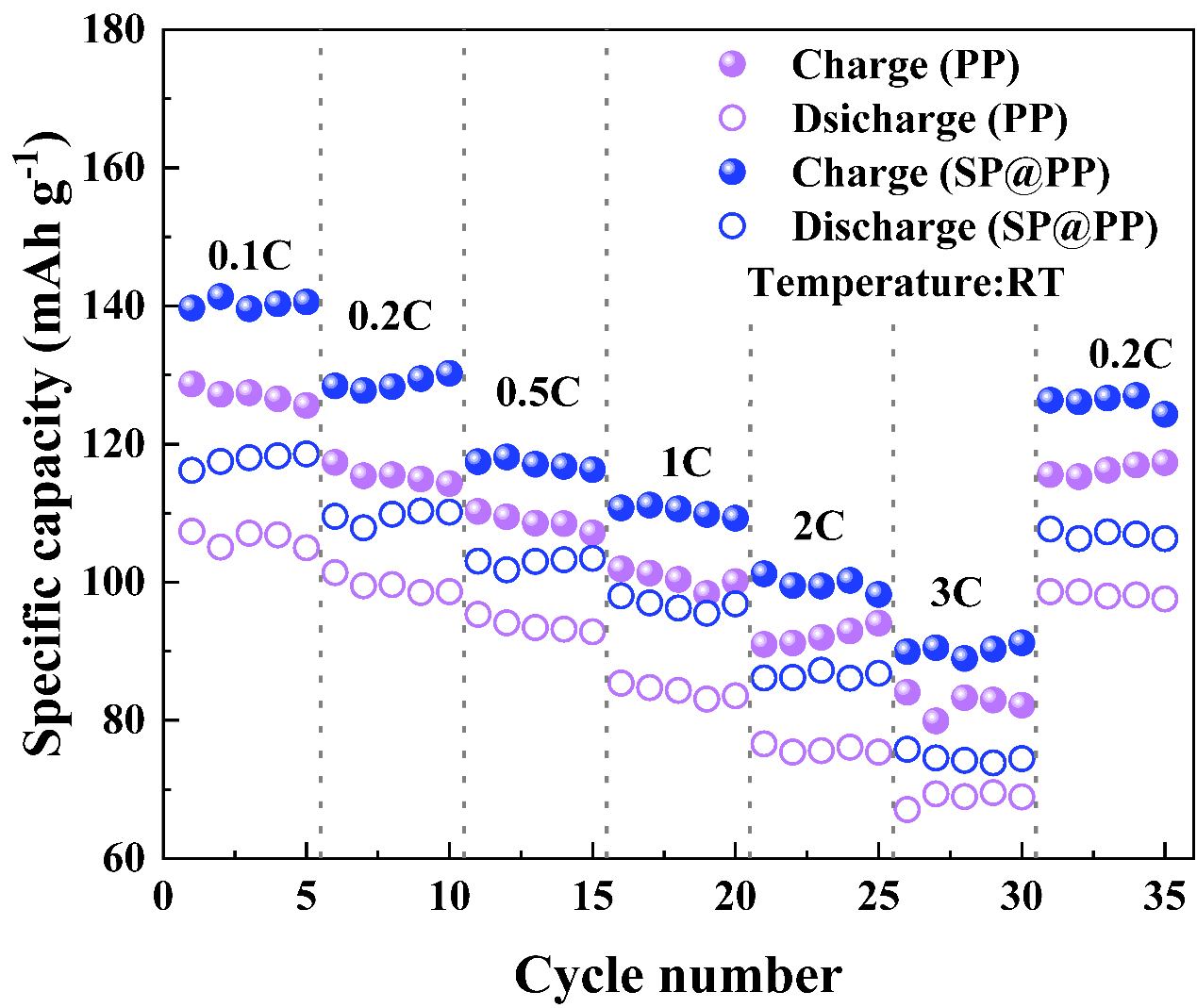


**Fig. S43.** Rate performance of batteries assembled with PP and SP@PP separators at ambient temperature.


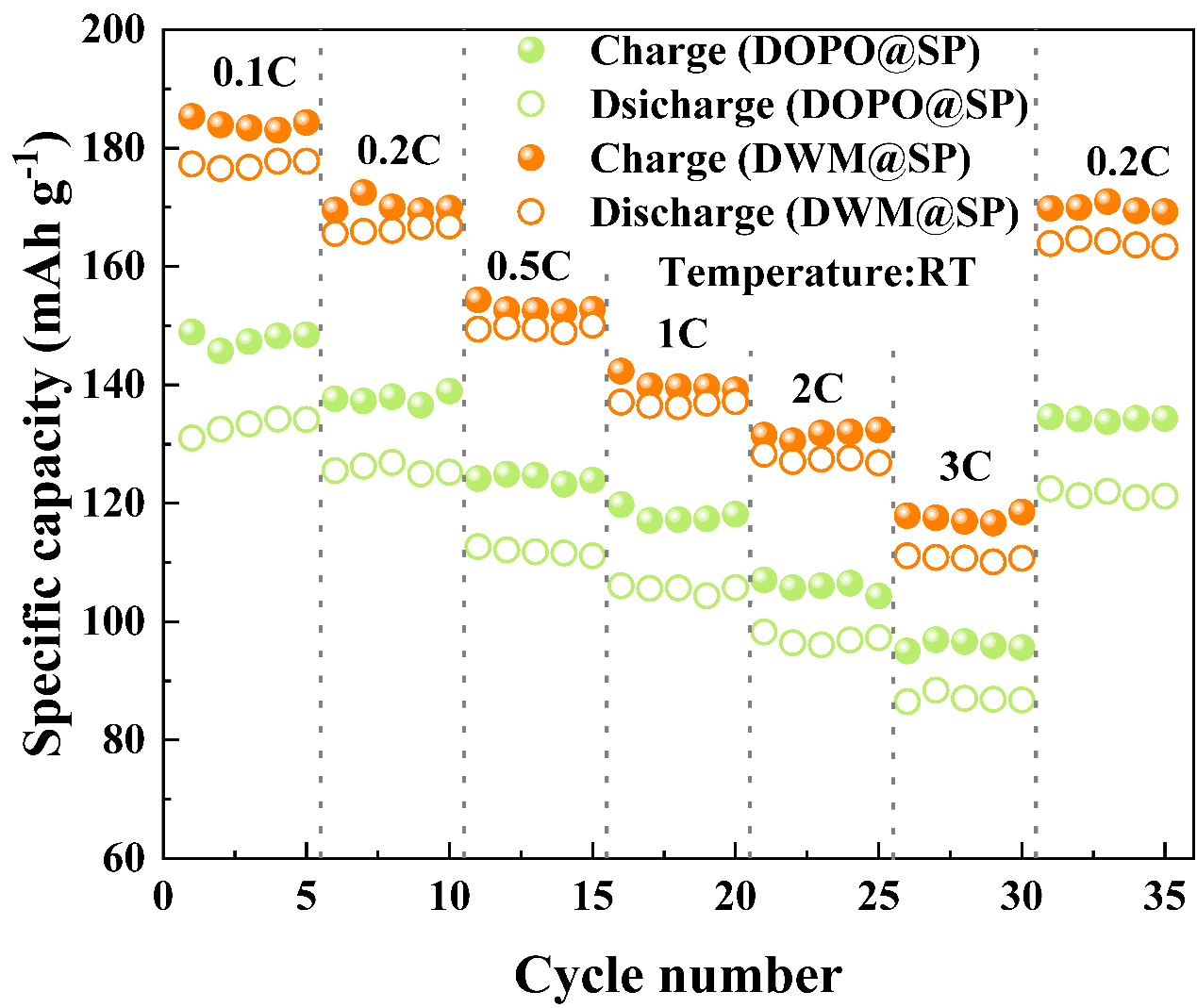


**Fig. S44.** Rate performance of batteries assembled with DOPO@SP and DWM@PP separators at ambient temperature.


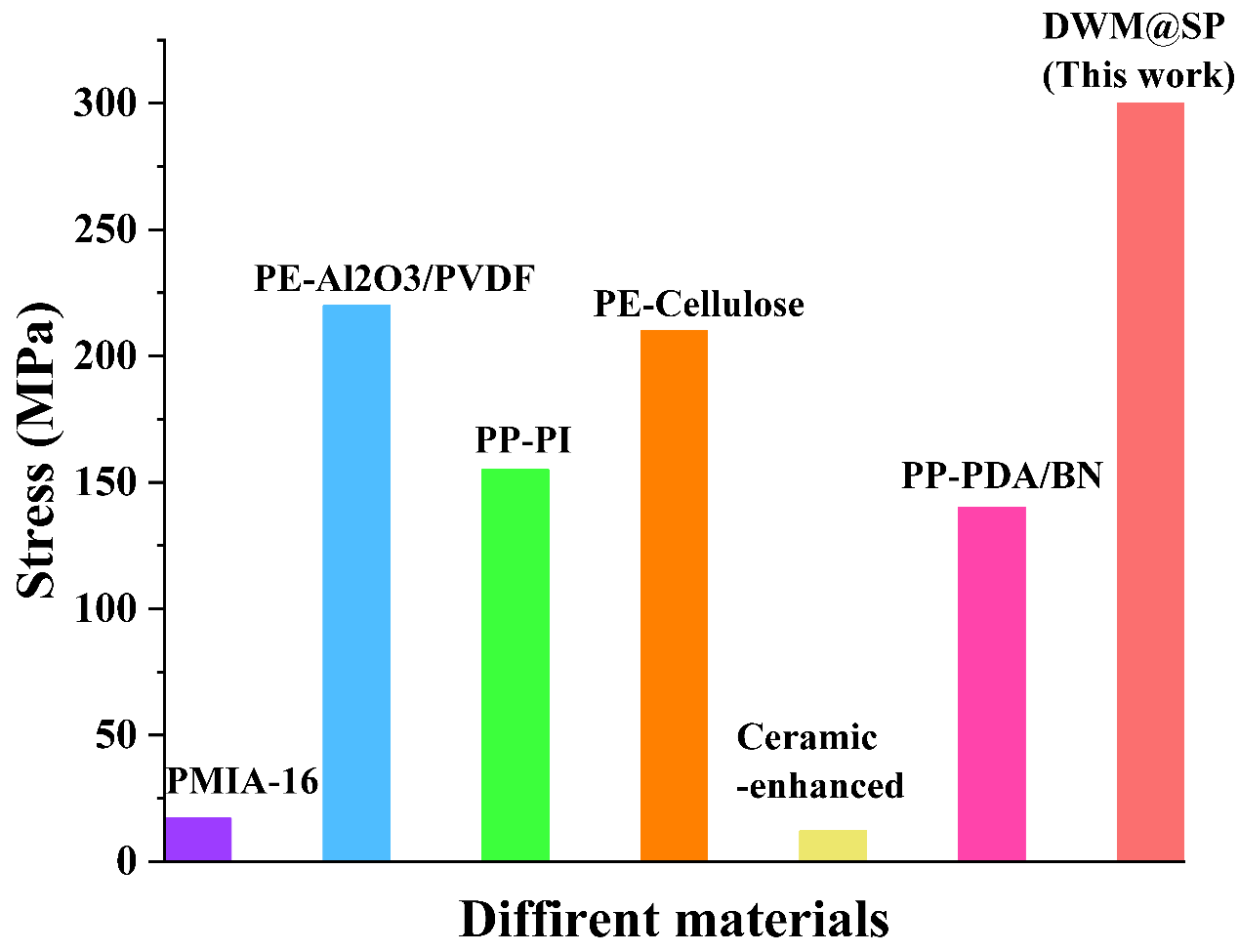


**Fig. S45.** Comparison of tensile stresses with other Seprators in operation.


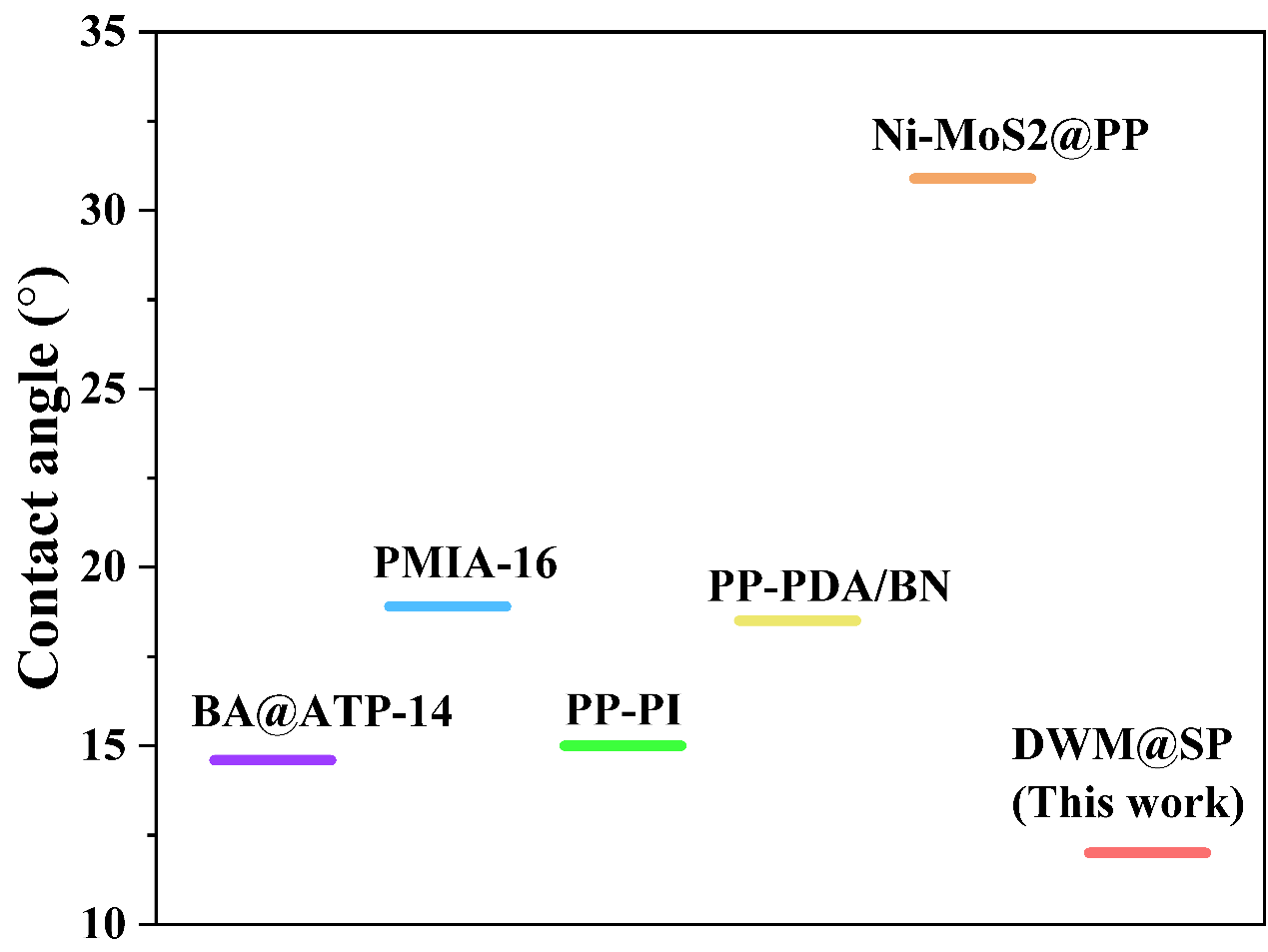


**Fig. S46.** Comparison of contact angles with other seperators in operation.

**
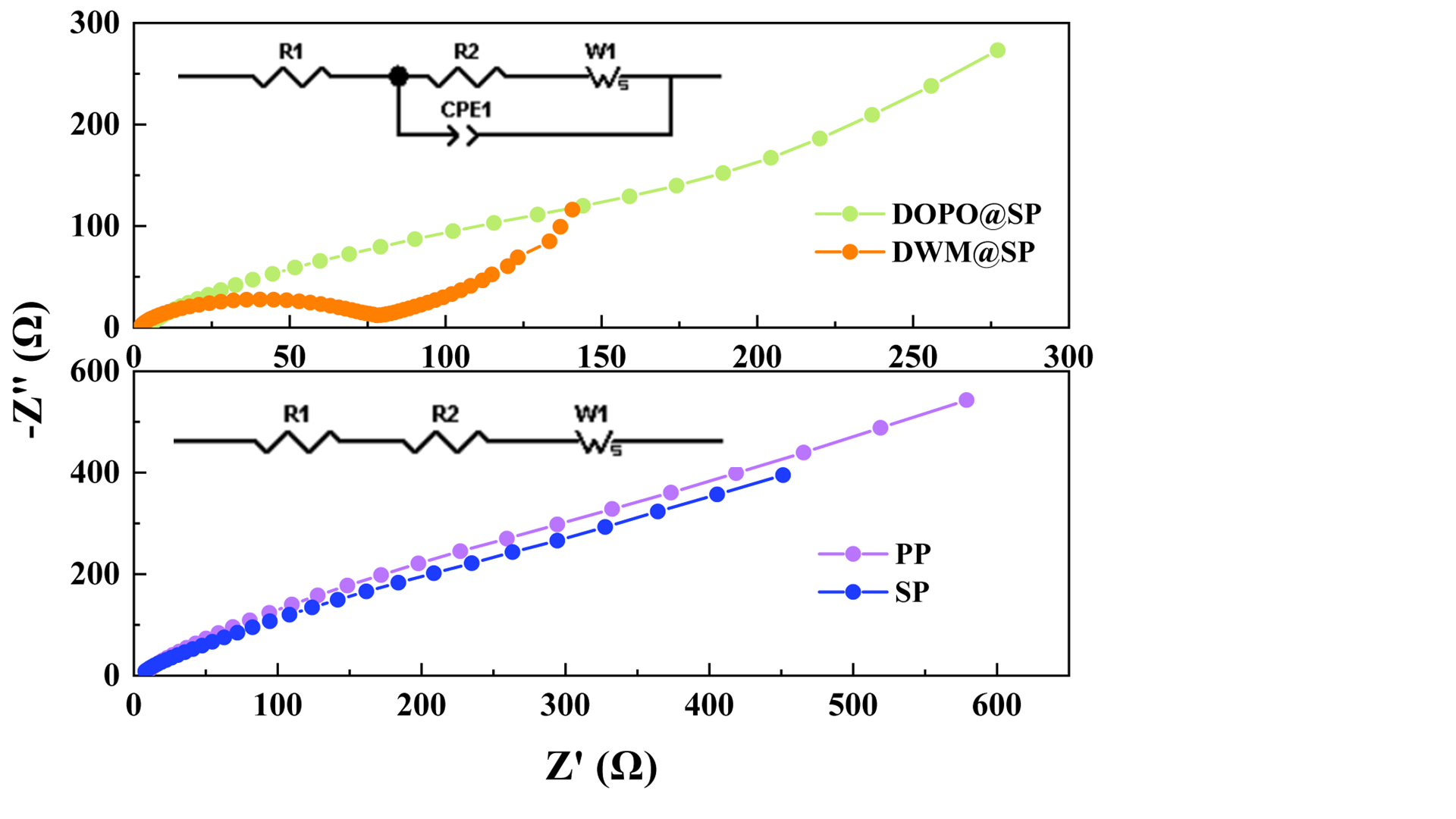
**

**Fig. S47.** EIS spectra of different seperator and their corresponding equivalent circuits.

**
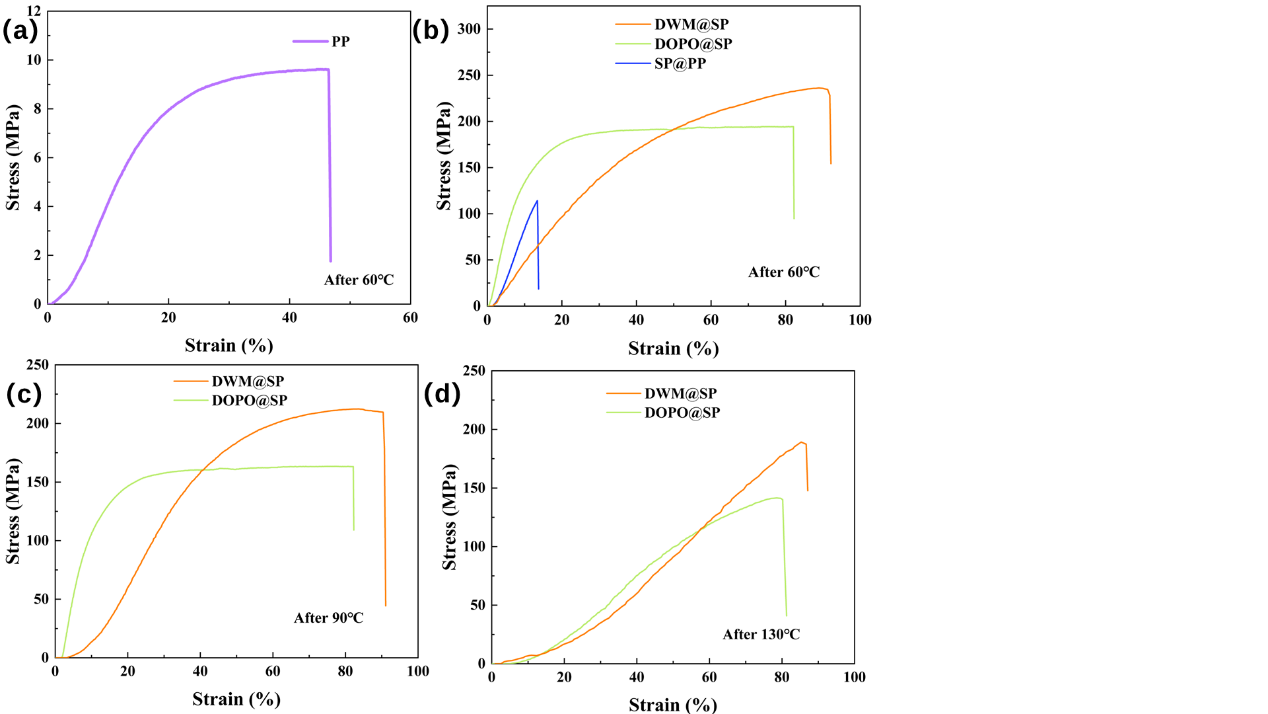
**

**Fig. S48.** Stress-strain curves of the diaphragm after testing at different temperatures. a , b at 60℃，b at 90℃and c at 130 ℃.


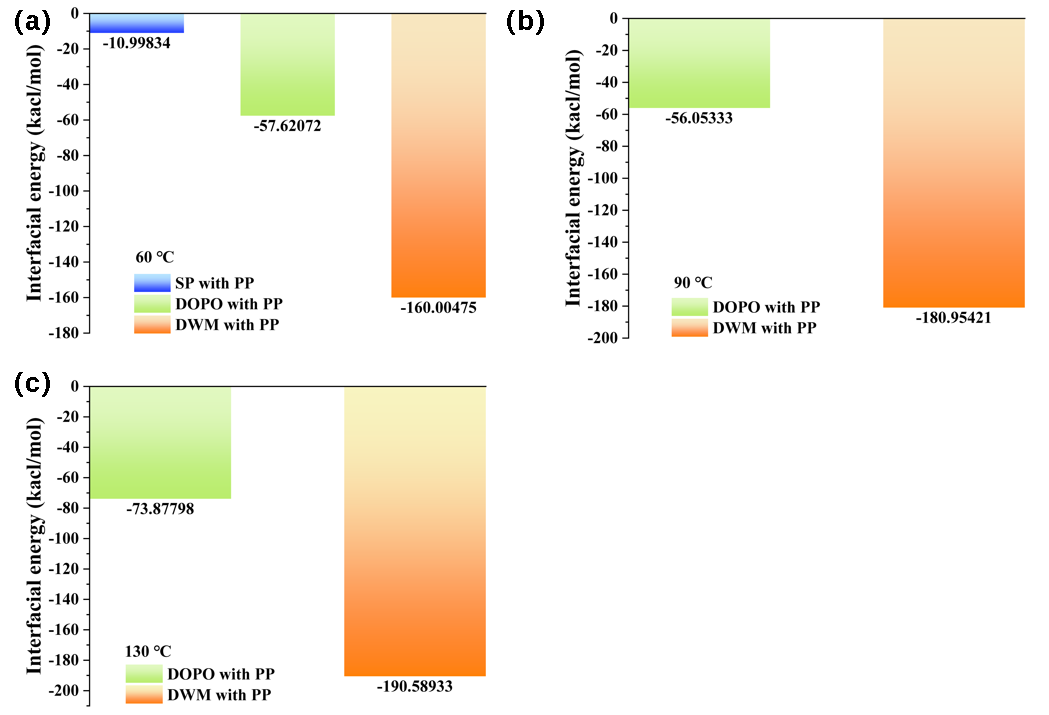


**Fig. S49.** Interfacial binding energy between different coatings and PP at 60°C(a), 90°C(b), and 130°C(c).

**
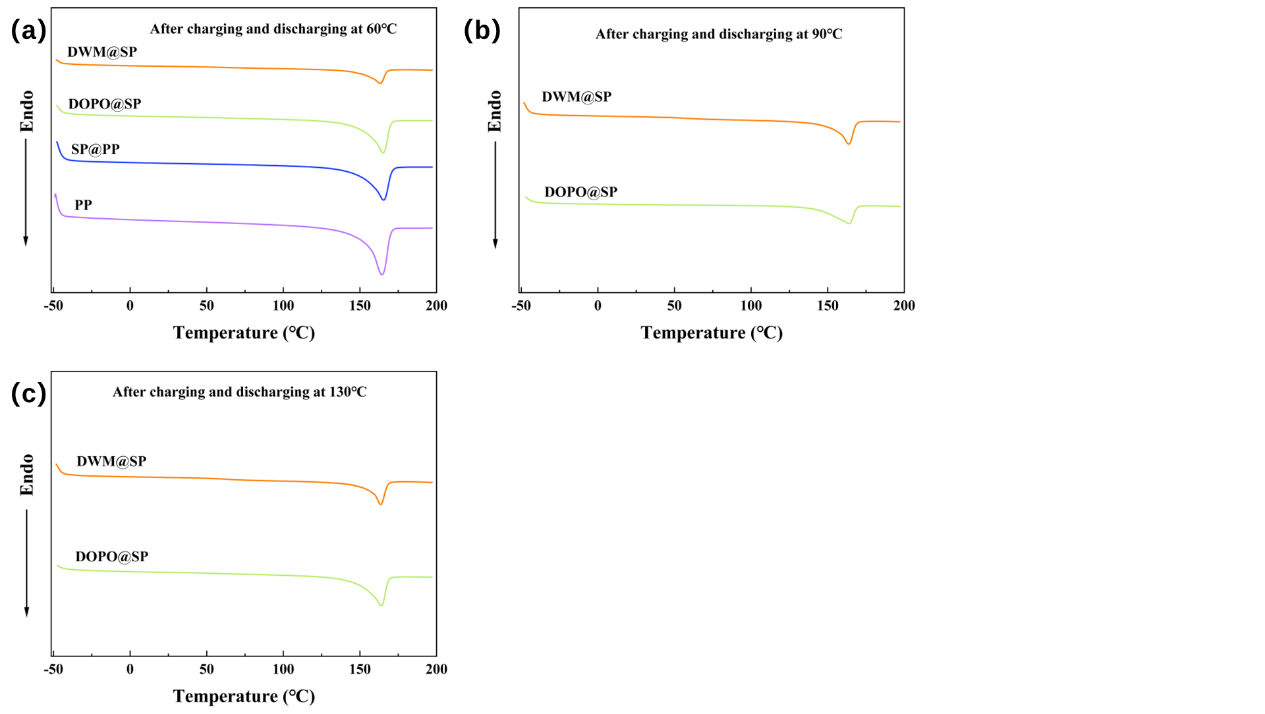
**

**Fig. S50.** DSC of seperator after testing at different temperatures.a at 60℃, b at 90℃and c at 130 ℃.

**
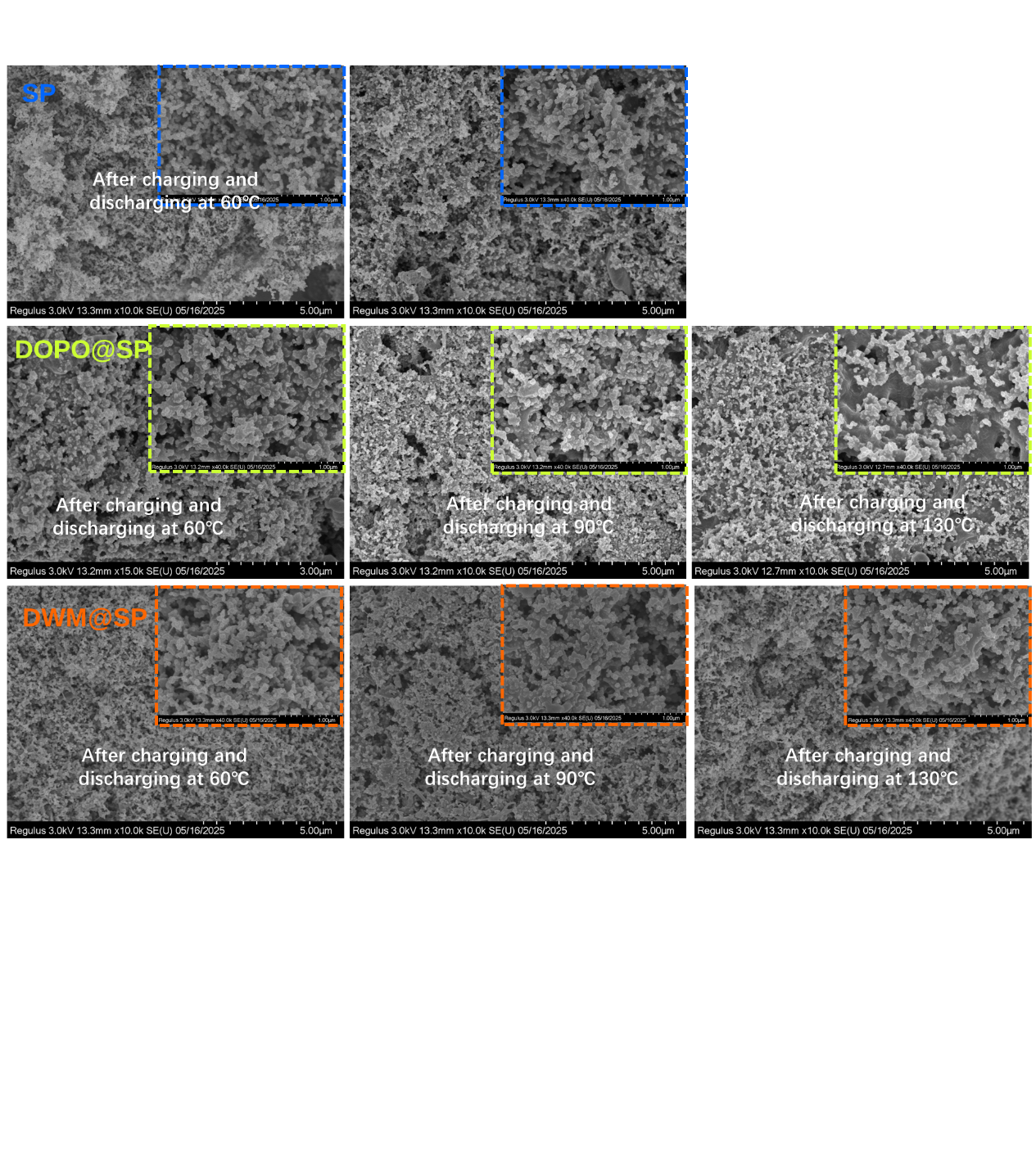
**

**Fig. S51.** SEM images of the coating after testing at different temperatures.

**
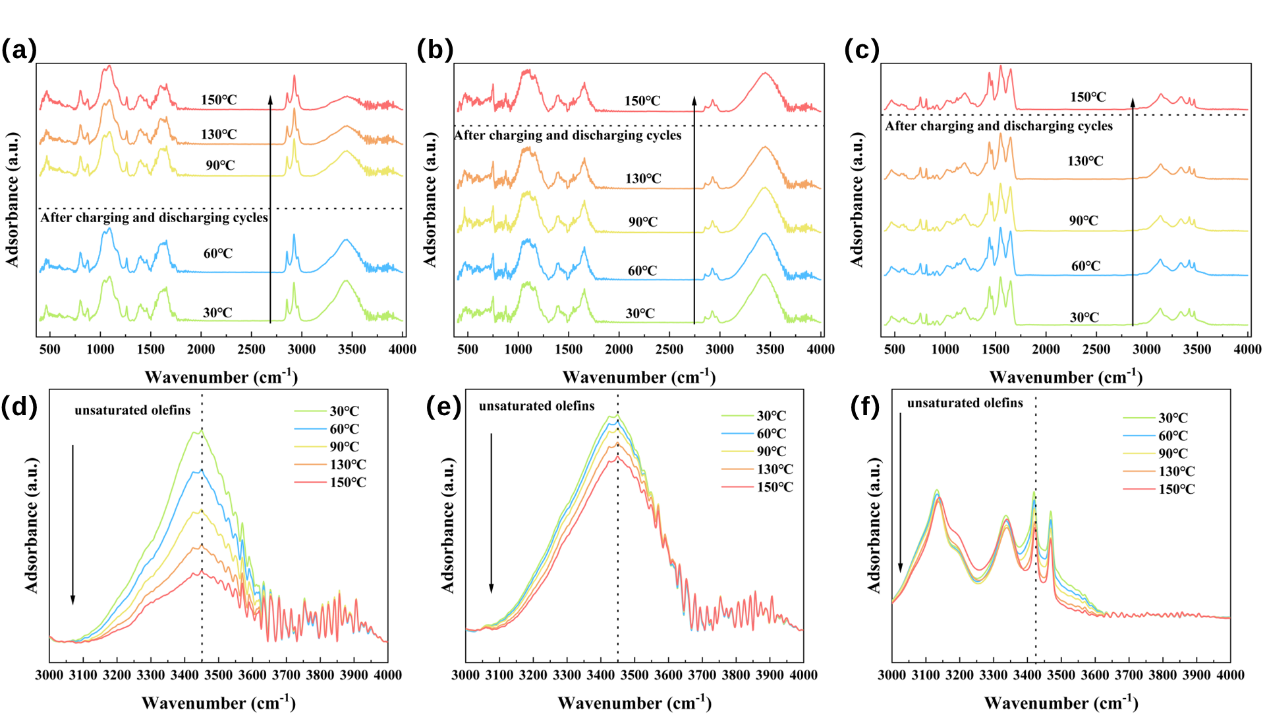
**

**Fig. S52.** FTIR spectra of the coating after testing at different temperatures. a , d SP@PP, b, e DOPO@SP and c, f DWM@SP.


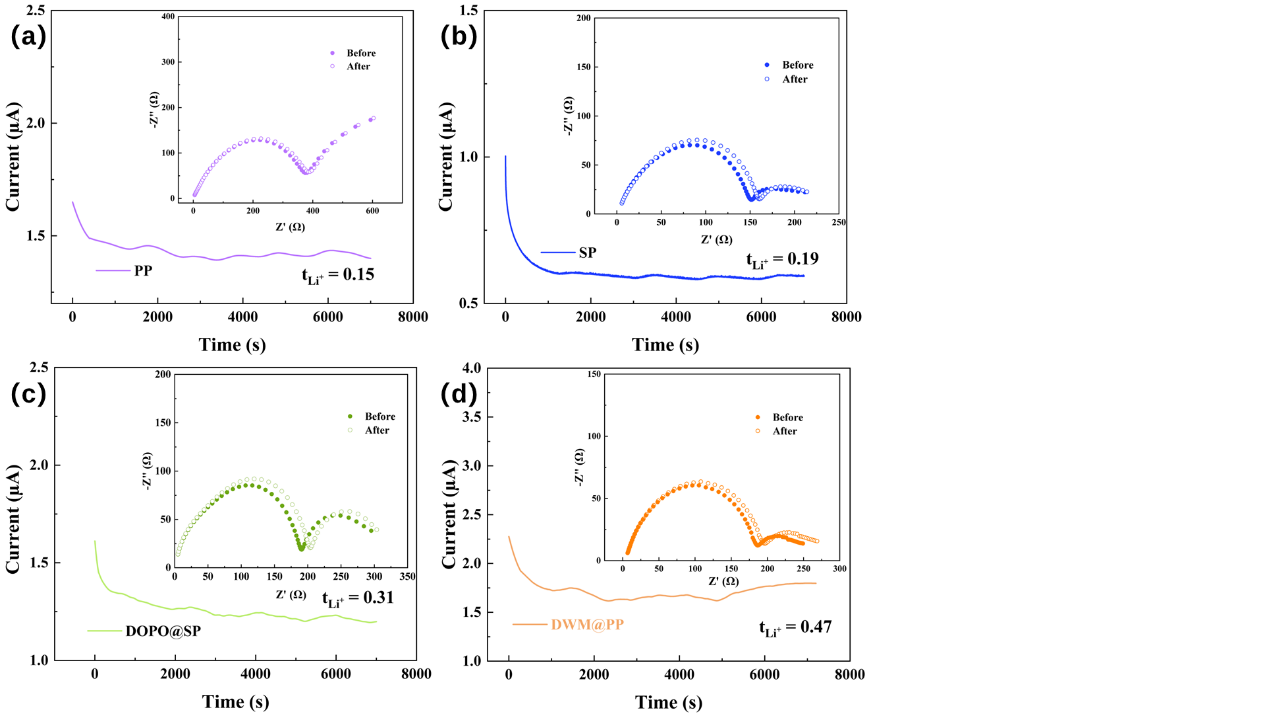


**Fig. S53.** The lithium-ion transference number test of seperator at 90ºC. a

d Chronoamperometry profiles and e-h AC impedance spectra (before and after

polarization) of PP, SP@PP, DOPO@SP and DWM@SP, respectively.

**
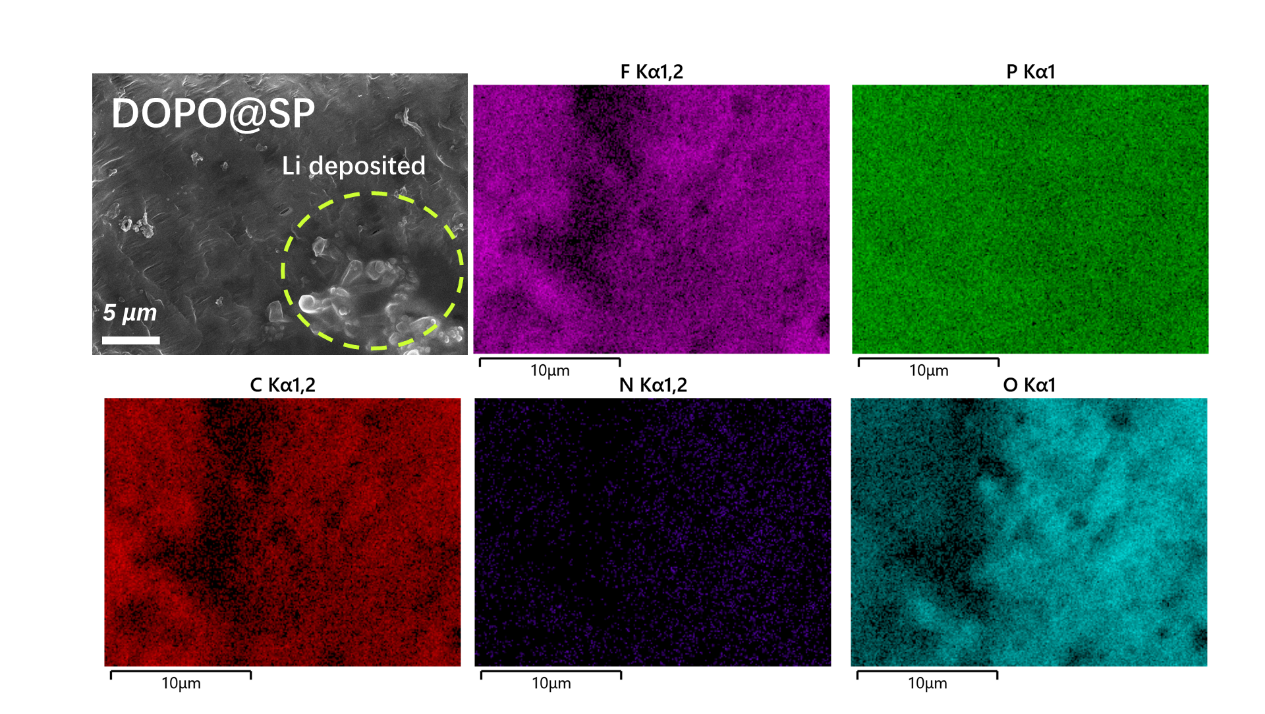
**

**Fig. S54.** SEM and EDS spectra of DOPO@SP membrane after high-temperature testing.

**
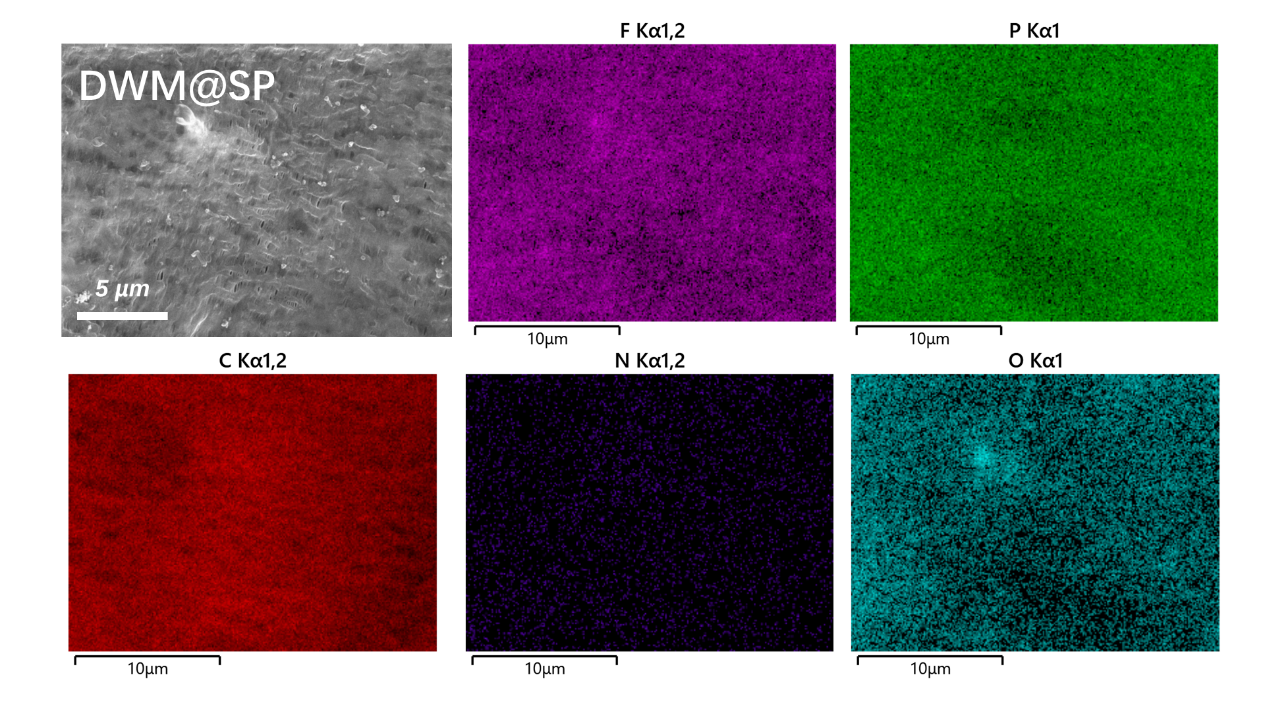
**

**Fig. S55.** SEM and EDS spectra of DWM@SP membrane after high-temperature testing.

**
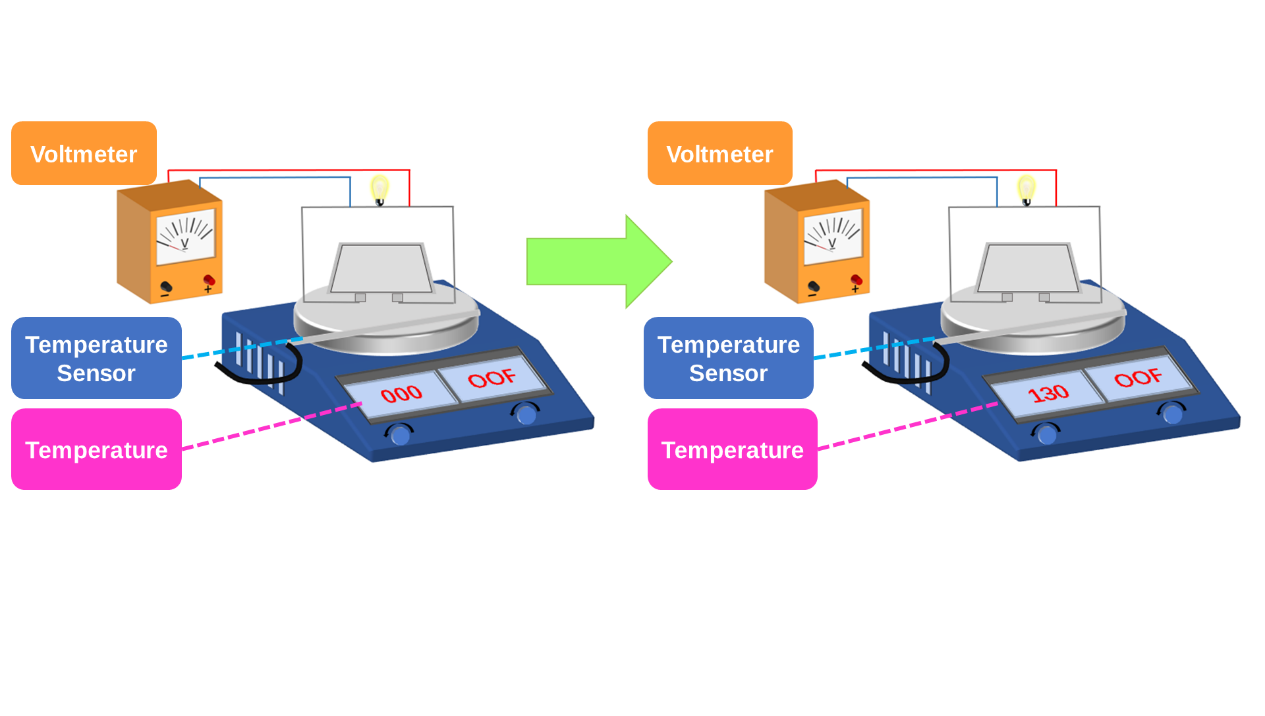
**

**Fig. S56.** Schematic diagram of the thermal abuse experiment device.

A flat heater was used as the heat source, and the pouch cell was monitored by a tightly

fitted temperature sensor. The voltmeter was directly connected to the positive and

negative poles of the battery to detect real-time changes in the voltage of the small bulb.

**
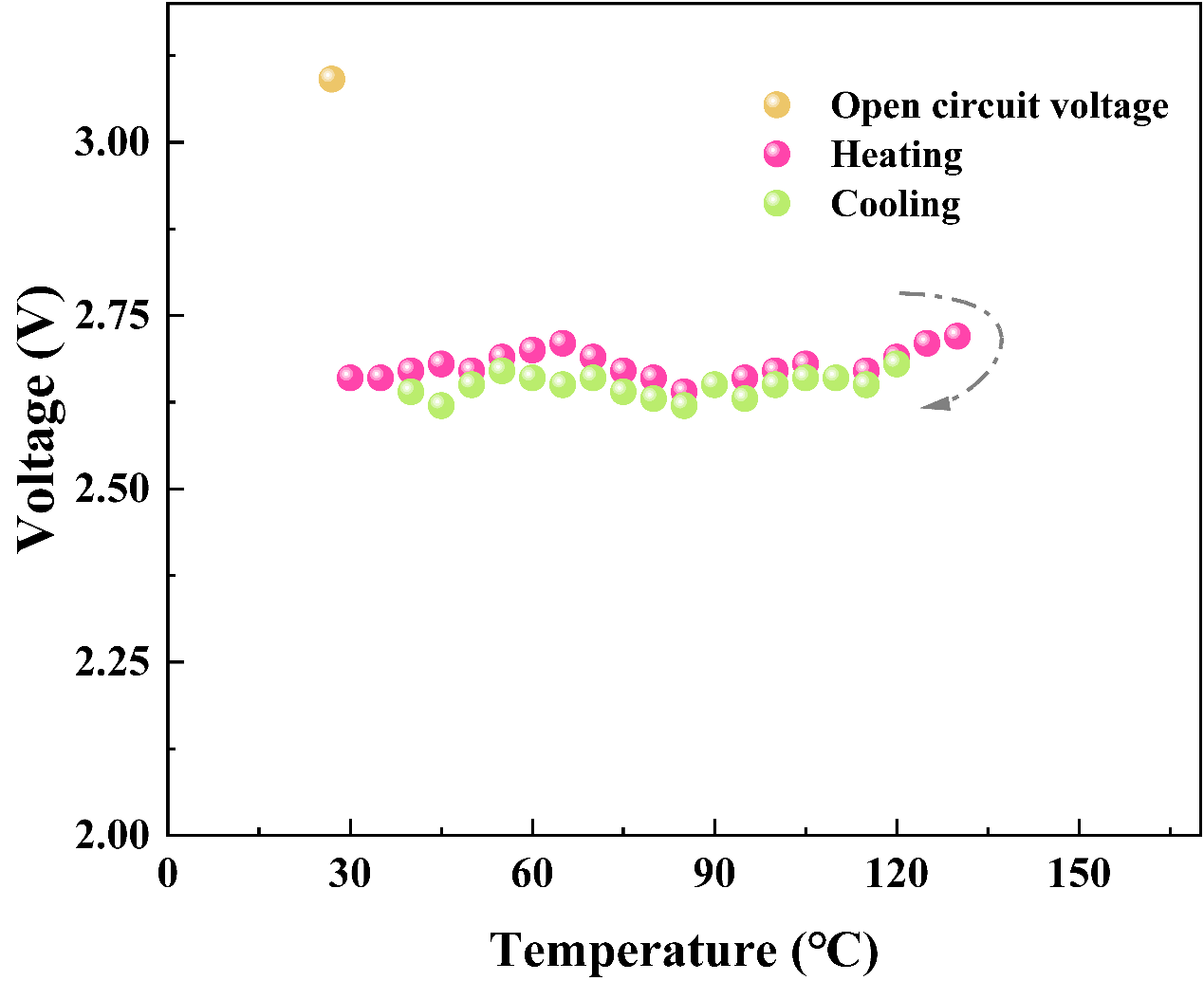
**

**Fig. S57.** Variation of the operating voltage of the pouch cell in thermal abuse test.

**
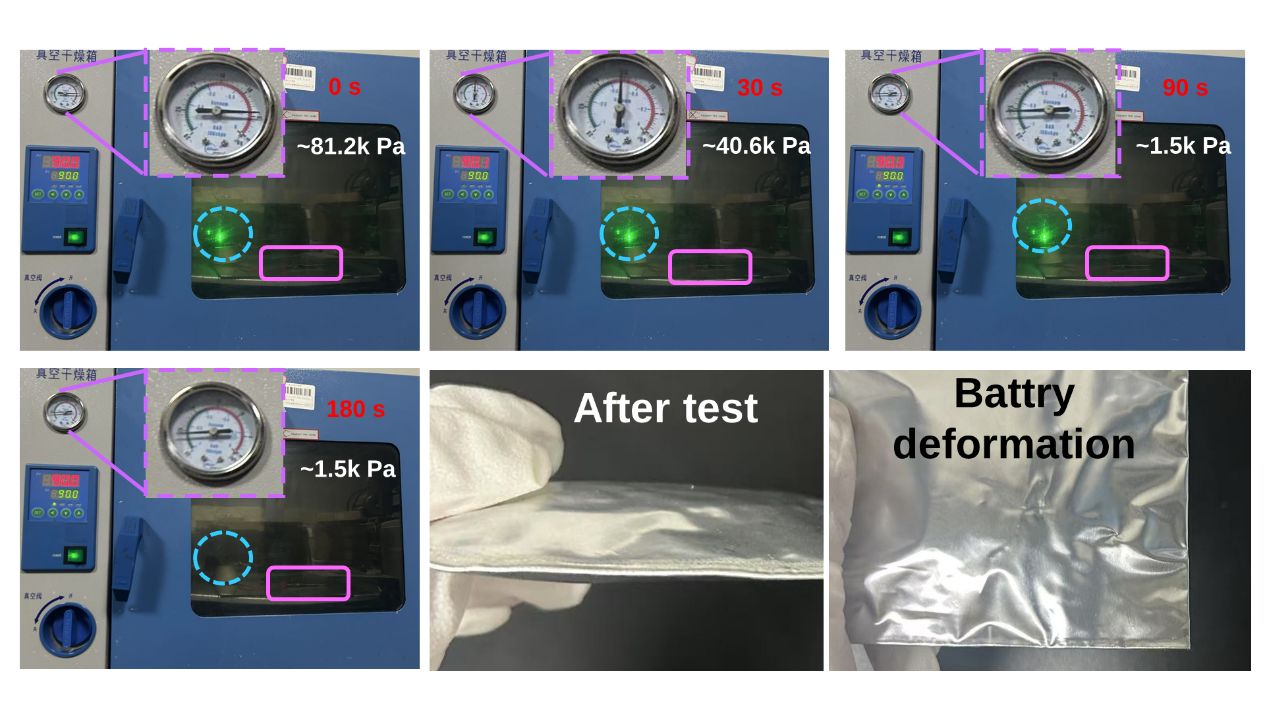
**

**Fig. S58.** PP seperator ignificant expansion (Red wireframe) and failure (LED bulb goes off in blue

circle) were observed at low vacuum. The battery after the test was significantly

deformed.

Table S1.Shrinkage rates in high-temperature ageing tests for different seperators.

| Samples | PP | SP@PP | DOPO@SP | DWM@SP |
| --- | --- | --- | --- | --- |
| Area shrinkage rate(before) | 100% | 100% | 100% | 100% |
| Area shrinkage rate(after) | 36% | 45% | 24% | 2% |

**Table. S2**. The ionic conductivity and activation energy ( *E_a_* )for different diaphragms at different temperatures and the activation energy ( *E_a_* ) values were calculated from the Arrhenius equation.

| Temperature  （℃） | | Ionic conductivity (σ,S·cm^-1^) | | | |
| --- | --- | --- | --- | --- | --- |
|  |  | PP | SP@PP | DOPO@SP | DWM@SP |
| 25 | | 1.18E-03 | 2.52E-03 | 1.82E-03 | 2.03E-03 |
| 30 | | 8.23E-04 | 1.60E-03 | 1.67E-03 | 2.15E-03 |
| 40 | | 5.32E-04 | 1.03E-03 | 1.62E-03 | 2.09E-03 |
| 50 | | 4.13E-04 | 6.59E-04 | 1.47E-03 | 2.16E-03 |
| 60 | | 2.85E-04 | 5.30E-04 | 1.38E-03 | 2.16E-03 |
| 70 | | — | — | 1.30E-03 | 2.23E-03 |
| 80 | | — | — | 1.21E-03 | 2.30E-03 |
| 90 | | — | — | 1.23E-03 | 2.39E-03 |
| 130 | | — | — | 1.27 E-03 | 2.46E-03 |
| Arrhenius Ea ( KJ mol^-1^ ) | 17.46 | | 12.83 | 8.16 | 6.49 |

Note: PP and SP@PP diaphragms will curl beyond 60 °C making accurate measurement of ionic conductivity below 60 °C impossible.

**Table. S3**. List of EIS fitting parameters for PP and SP diaphragms.

| **Sample** | **（R_1_）Rs/Ω**  **(Error/%)** | **（R_2­_）R_f_/Ω**  **(Error/%)** | **W_1_/Ω**  **(Error/%)** |
| --- | --- | --- | --- |
| PP | 38. 3 ( 0.97 ) | 191.01 ( 9.02 ) | 2.91 ( 2.71 ) |
| SP | 31.7 ( 4.73 ) | 170.36 ( 6.13 ) | 2.66 ( 0.18 ) |

**Table. S4**. List of EIS fitting parameters for DOPO@SP and DWM@SP diaphragms.

| **Sample** | **（R_1_）Rs/Ω**  **(Error/%)** | **（R_2­_）R_f_/Ω**  **(Error/%)** | **W_1_/Ω**  **(Error/%)** |
| --- | --- | --- | --- |
| DOPO@SP | 3.06 ( 1.97 ) | 93. 6 ( 11.76 ) | 0.816 ( 0.51 ) |
| DWM@SP | 1.392 ( 0.531 ) | 77.09 ( 0.4311 ) | 0.627 ( 0.18 ) |

1. ^1^ Contribute equally as the first author.

   *Corresponding author. [↑](#footnote-ref-1)
2. E-mail address: tangwf@huse.edu.cn (W. Tang).

   E-mail address: pengzhihan@dhu.edu.cn (Z. Peng). [↑](#footnote-ref-2)
3. E-mail address: [yaohq@nxmu.edu.cn](mailto:yaohq@nxmu.edu.cn) (H. Yao). [↑](#footnote-ref-3)
4. E-mail address: [Mujy@nxmu.edu.cn](mailto:Mujy@nxmu.edu.cn) (J.Mu). [↑](#footnote-ref-4)
